# Supplementary material for: Short-term multicomponent exercise training improves executive function in postmenopausal women
Source: PLoS One. 2024 Aug 14;19(8):e0307812. doi: 10.1371/journal.pone.0307812 (PMC11324113; doi:10.1371/journal.pone.0307812)
Supplement: S1 Table — (PDF) [file pone.0307812.s002.pdf]

S1 Table. Exercise intervention protocol

Component of exercise	Format	Exercises	Examples of exercises	Intensity	Time
**Week 1**					
Aerobics	Group	Brisk walking	Marching on the spot (with arm exercise), single knee lift, single side step, single tap, cross step, step forward/backward, kick	5-6/10 RPE	10 minutes
Strength	Group	All major upper, lower body and core muscle groups (using loop band)	Band biceps curl, Band triceps extension, Band chest press, Band shoulder press, Band bridge abs, Band Squat, Band leg press, lateral band walk	2 sets, 6 reps	10 minutes
Balance	Group	Static balance with two arms support	Stand with feet side by side, tandem walking, one leg stand, heel to toe	N/A	10 minutes
Flexibility and cool down	Group	Static stretch	Neck stretch, arm stretch, shoulder stretch, back stretch, hip stretch, leg stretch, butterfly stretch, cat and camel	2 sets, 6 count	10 minutes
				Total time	40 minutes
**Week 2**					
Aerobics	Group	Brisk walking	Marching on the spot (with arm exercise), single/double knee lift, single/double side step, single/double tap, cross step, step forward/backward, kick	5-6/10 RPE	20 minutes
Strength	Group	All major upper, lower body and core muscle groups (using loop band)	Band biceps curl, Band triceps extension, Band chest press, Band shoulder press, Band bridge abs, Band Squat, Band leg press, lateral band walk	2 sets 8 reps	15 minutes
Balance	Group	Static balance without support	Stand with feet side by side, tandem walking, one leg stand, heel to toe	N/A	10 minutes
Flexibility Stretching and cool down	Group	Static stretch	Neck stretch, arm stretch, shoulder stretch, back stretch, hip stretch, leg stretch, butterfly stretch, cat and camel	2 sets, 8 count	15 minutes
				Total time	60 minutes

.
