# Supplementary material for: Short-term multicomponent exercise training improves executive function in postmenopausal women
Source: PLoS One. 2024 Aug 14;19(8):e0307812. doi: 10.1371/journal.pone.0307812 (PMC11324113; doi:10.1371/journal.pone.0307812)
Supplement: S1 File — (PDF) [file pone.0307812.s003.pdf]

Protokol Etik Penelitian Kesehatan
Yang Mengikutsertakan Manusia Sebagai Subjek

Isilah form dibawah dengan uraian singkat dan berikan tanda contreng (X/V) pada kotak atau lingkari pada salah satu pilihan jawaban yang menggambarkan penelitian.
P: Nomor Urutan Protokol CIOMS 2016 – Lampiran 1;
S: Standar Kelaikan Etik (WHO-2011 dan Pedoman KEPPKN 2017);
C: Check List/Daftar Tilik
G: Guideline CIOMS 2016
IC: CIOMS 2016 – Lampiran 2

Daftar Isi:

A. Judul Penelitian (p-protokol no 1)*
B. Ringkasan usulan penelitian (p-protokol no 2)
C. Isu Etik yang mungkin dihadapi
D. Ringkasan Daftar Pustaka
E. Kondisi Lapangan
F. Desain Penelitian
G. Sampling
H. Intervensi
I. Monitor Hasil
J. Penghentian Penelitian dan Alasannya
K. Adverse Event dan Komplikasi (Kejadian Yang Tidak Diharapkan)
L. Penanganan Komplikasi
M. Manfaat
N. Jaminan Keberlanjutan Manfaat
O. Informed Consent
P. Wali
Q. Bujukan
R. Penjagaan Kerahasiaan
S. Rencana Analisis
T. Monitor Keamanan
U. Konflik Kepentingan
V. Manfaat Sosial
W. Hak atas Data
X. Publikasi
Y. Pendanaan
Z. Komitmen Etik
AA. Daftar Pustaka
AB. Lampiran
1. CV Peneliti Utama
2. Sampel Formulir Laporan kasus

Protokol Etik Penelitian Kesehatan
Yang Mengikutsertakan Manusia Sebagai Subjek

Isilah form dibawah dengan uraian singkat dan berikan tanda contreng (X/V) pada kotak atau lingkari pada salah satu pilihan jawaban yang menggambarkan penelitian.
P: Nomor Urutan Protokol CIOMS 2016 – Lampiran 1;
S: Standar Kelaikan Etik (WHO-2011 dan Pedoman KEPPKN 2017);
C: Check List/Daftar Tilik
G: Guideline CIOMS 2016
IC: CIOMS 2016 – Lampiran 2

A. Judul Penelitian (p-protokol no 1)*

Pengaruh latihan kombinasi aerobic (jalan cepat), kekuatan dan keseimbangan terhadapa fungsi kognitif dan kesehatan mental pada wanita menopause.

1. Lokasi Penelitian:
   Lapangan Thor, Surabaya
2. Waktu Penelitian direncanakan (mulai – selesai)
   Februari 2023- Februari 2024 2023

	Ya	Tidak
3. Apakah penelitian ini multi-senter	☐	√
4. Jika Multi senter apakah sudah mendapatkan persetujuan etik dari senter/institusi yang lain (lampirkan jika sudah)	☐	√

Identifikasi (p10)

1. Peneliti
   (Mohon CV Peneliti Utama dilampirkan)
   Peneliti Utama (PI) : Dani Rahmat Ramadhana., S.Pd
   Institusi : Fakultas Kedokteran, Universitas Airlangga
2. Anggota Peneliti : Raden Argarini dr., M.Kes., Ph.D
   Institusi : Fakultas Kedokteran, Universitas Airlangga
3. Sponsor (p9)
   Nama : -
   Alamat : -

B. Ringkasan usulan penelitian (p-protokol no 2)

1. Ringkasan dalam 200-300 kata (ditulis dalam bahasa yang mudah dipahami oleh “awam” bukan dokter/profesi)

   Menopause merupakan salah satu proses dalam siklus reproduksi alamiah yang akan dialami oleh wanita selain pubertas, kehamilan dan menstruasi. Hal ini ditandai dengan absennya menstruasi dan merupakan akhir dari masa fertilitas. Selama menopause, wanita mengalami tanda dan gejala akibat penurunan kadar estrogen seperti instabilitas vasomotor, atrofi genitalia, dan gangguan mood. Wanita pasca menopause juga mengalami gangguan kognitif, diantaranya penurunan daya ingat atau defisit memori, gangguan konsentrasi, perubahan mood dan perilaku. Gejala-gejala tersebut mengakibatkan penurunan kualitas hidup dan ketidaknyamanan dalam melakukan aktivitas sehari hari. Latihan fisik merupakan salah satu cara untuk dapat meningkatkan fungsi kognitif pada wanita pasca menopause. Latihan fisik yang mengkombinasikan beberapa jenis olahraga diduga berpotensi menghasilkan peningkatan fungsi kognitif yang lebih besar dibandingkan dengan latihan tunggal karena merangsang berbagai area otak yang berbeda. Penelitian ini bertujuan untuk menganalisis pengaruh program latihan kombinasi jangka pendek terhadap fungsi kognitif pada wanita pascamenopause. Subjek pada penelitian ini adalah wanita berusia 50-80 tahun, yang setidaknya 12 bulan pasca menopause dengan tingkat pendidikan minimal sekolah menengah pertama atau setara. Subjek penelitian akan direkrut dari kelompok program Pemberdayaan Kesejahteraan Keluarga (PKK). Subjek kemudian dialokasikan ke dalam kelompok kontrol dan kelompok intervensi olahraga. Subjek pada kelompok olahraga akan mendapatkan latihan kombinasi dengan intensitas sedang yang terdiri dari latihan aerobik, kekuatan, fleksibilitas, dan keseimbangan. Latihan dilakukan dengan frekuensi 5x/minggu selama 2 minggu. Pada minggu pertama, setiap sesi berlangsung selama 40 menit dan secara bertahap meningkat menjadi 60 menit. Penilaian fungsi kognitif dilakukan dengan tes Stroop untuk menilai fungsi eksekutif dan *Mini-Mental State Examination* (MMSE) untuk fungsi kognitif umum. Selain itu, akan dilakukan pengukuran stres psikologis pada subjek dengan menggunakan Depression Anxiety Stress Scale-21 (DASS-21).

2. Justifikasi penelitian (p3). Tuliskan mengapa penelitian ini harus dilakukan, manfaatnya untuk penduduk di wilayah penelitian ini dilakukan (Negara, wilayah, lokal)- Standar 2/A (Adil)

   Pada tahun 2030, jumlah wanita di seluruh dunia yang memasuki masa menopause diperkirakan mencapai 1,2 miliar (WHO, 2014). Seiring dengan peningkatan usia harapan hidup wanita di Indonesia, akan semakin banyak wanita yang menjalani masa kehidupannya pada periode menopause dan diperkirakan mencapai 60 juta pada tahun 2025 (WHO, 2014). Di Indonesia, pada umumnya wanita akan mengalami usia menopause antara 44 sampai 45 tahun, lebih muda dibandingkan dengan wanita di negara maju (>47 tahun). Hal ini disebabkan karena taraf sosial ekonomi, pendidikan, gizi, dan kesehatan di negara maju lebih baik dibandingkan negara berkembang seperti Indonesia. Seiring dengan peningkatan usia wanita menopause, maka terjadi peningkatan gangguan kesehatan terkait menopause (Sulisetyawati, 2011).

   Namun hingga saat ini belum ada penelitian yang meneliti pengaruh latihan kombinasi aerobic (jalan cepat), kekuatan, dan keseimbangan terhadap fungsi kognitif dan Kesehatan mental wanita menopause. Berdasarkan hal tersebut, penelitian ini akan mengeksplorasi pengaruh latihan fisik berupa kombinasi latihan aerobik (berjalan cepat), latihan penguatan otot dan latihan keseimbangan terhadap fungsi kognitif dan kesehata mentalpada wanita post-menopause.

B. Isu Etik yang mungkin dihadapi

1. Pendapat peneliti tentang isu etik yang mungkin dihadapi dalam penelitian ini, dan bagaimana cara menanganinya (p4) – sesuaikan dengan 7 butir standar kelaikan etik (S) dan G berapa

   Penelitian ini dilakukan terhadap subjek wanita meopause yang rentang usianya tergolong dalaam lansia. Lansia akan lebih rawan mengalami cedera baik musculoskeletal ataupun kardiorespirai, namun hal tersebut diantisipasi dengan penerapan intensitas yang tepat dan juga didampingi oleh presonal trainer serta dokter. Apabilas terjadi cedera, maka penangann pertama dialkukan dengan metode *Protection, Rest, Ice, Compression,* dan *Elevation* (PRICE).

C. Ringkasan Daftar Pustaka

1. Ringkasan hasil hasil studi sebelumnya sesuai topik penelitian, termasuk yang belum dipublikasi yang diketahui para peneliti dan sponsor, dan informasi penelitian yang sudah dipublikasi, termasuk jika ada kajian-kajian pada hewan. Maksimum 1 hal (p5)- G 4

   Menopause adalah berhentinya menstruasi secara alami yang terjadi pada wanita antara 45-55 tahun, namun dapat bervariasi dan sangat dipengaruhi oleh beberapa faktor baik faktor fisiologis maupun psikologis (Chaturvedi et al., 2016). Wanita menopause akan mengalami gangguan kognitif, diantaranya penurunan daya ingat atau defisit memori, gangguan konsentrasi, perubahan mood dan perilaku (Whitmer et al., 2011). Gejala gejala tersebut mengakibatkan penurunan kualitas hidup dan ketidaknyamanan dalam melakukan aktivitas sehari-hari (Thurston et al., 2011, Discigil et al., 2006). Hormon estrogen berfungsi sebagai neuroprotektif, jika terjadi penurunan hormon estrogen proses yang mengenai area ventral hipokampus dapat menyebabkan gangguan memroi dan kognitif pada wanita menopause (Cutter et al., 2003). Selain itu perubahan fungsi memori dan kognitif pada wanita menopause juga berhubungan dengan penurunan ekspresi *Brain-Derived Neurotrophic Factor* (BDNF) di hipokampus dan korteks serebri (Erickson et al., 2010). Latihan fisik mungkin merupakan cara yang efektif untuk mencegah atau mengurangi gangguan kognitif pada wanita menopause. Dalam studi observasional cross-sectional pada wanita menopause, menunjukkan bahwa latihan fisik yang berbentuk senam aerobik low impact berpengaruh terhadap pengurangan keluhan vasomotorik, keluhan psikis dan keluhan somatik pada menopause (Sasnitiari, 2018). Peningkatan kualitas hidup sangat dipengaruhi oleh peningkatan fungsi kognitif, oleh karena itu baik latihan aerobik dan kekuatan dikaitkan dengan peningkatan fungsi kognitif seperti fungsi eksekutif, kontrol penghambatan, dan memori episodik (Liu & Eden, 2008). Efek neuroprotektif dari olahraga dapat dimediasi oleh peningkatan faktor neurotropik yang diturunkan dari otak misalnya BDNF, faktor pertumbuhan seperti insulin tipe I (IGF-1), faktor pertumbuhan endotel vaskular (VEGF), dan homosistein yang diinduksi oleh olahraga (Vincent et al., 2003), sehingga mendorong perubahan struktural dan konektivitas di area otak yang penting untuk menigkatan fungsi memori dan kognitif, misalnya lobus frontal dan temporal dan hipokampus (Erickson et al., 2010). Namun hingga saat ini belum ada penelitian yang meneliti pengaruh latihan kombinasi aerobic (jalan cepat), kekuatan dan keseimbangan terhadap fungsi kognitif dan kualitas hidup pada wanita menopause

D. Kondisi Lapangan

1. Gambaran singkat tentang lokasi penelitian (p8) lihat G-2

   Penelitian akan dilakukan di Lapangan Thor Surabaya. Terdapat beberapa fasilitas lapangan indoor maupun outdoor serta ruangan-ruangan kecil yang dapat dipergunakan untuk melakukan tes maupun intervensi.

2. Informasi ketersediaan fasilitas yang layak untuk keamanan dan ketepatan penelitian,

   Pemeriksaan tekanan darah, denyut nadi, dan kuesioner kesehatan dilakukan di ruangan khusus pada tempat latihan. Alat yang digunakan telah terstandar dan tersedia di departemen Faal dan Biokimia Kedokteran Fakultas Kedokteran Universitas Airlangga. Pengukuran variabel yang dibutuhkan dilakukan oleh tenaga terlatih dengan jadwal yang telah ditentukan yaitu pada awal sebelum melakukan program intervensi latihan dan setelah 2 minggu menjalankan program intervensi latihan. Penelitian dilakukan sesuai dengan protokol kesehatan (PROKES) yang berlaku

3. Informasi demografis / epidemiologis yang relevan tentang daerah penelitian.
   Dengan jumlah penduduk yang mencapai sekitar 3.052.020 Orang di Tahun 2017, Kota Surabaya berkembang sebagai Kota Metropolitan. Posisi strategis Kota Surabaya sebagai pusat kegiatan ekonomi masyarakat membuatnya selalu dinamis. Surabaya memiliki iklim tropis seperti kota besar di Indonesia pada umumnya. Berdasarkan klasifikasi iklim Koppen, Kota Surabaya termasuk dalam kategori iklim tropis basah dan kering (*Aw*) dengan dua musim dalam setahun yaitu musim hujan dan musim kemarau. Curah hujan di Surabaya rata-rata 165,3 mm. Curah hujan tertinggi di atas 200 mm terjadi pada kurun Januari hingga Maret dan November hingga Desember. Suhu udara rata-rata di Surabaya berkisar antara

23,6 °C hingga 33,8 °C. Penelitian akan dilakukan luar ruangan yang aman dan nyaman agar suhu yang ekstrim tidak mempengaruhi performa subjek.

E. Desain Penelitian

1. Tujuan penelitian, hipotesis, pertanyaan penelitian, asumsi dan variabel penelitian (p11).

Pertanyaan penelitian:

- Apakah latihan kombinasi jangka pendek dapat meningkatkan fungsi kognitif pada wanita menopause?

Tujuan Penelitian:

- Membuktikan latihan kombinasi jangka pendek dapat meningkatkan fungsi kognitif dan kesehatan pada wanita menopause

Hipotesis:

- Latihan kombinasi jangka pendek dapat meningkatkan fungsi kognitif dan kesehatan mental pada wanita menopause

Variabel Penelitian:
Variabel bebas

- Latihan kombinasi jangka pendek

Variabel tergantung

- Fungsi Eksekutif
- Fungsi Kognitif global
- Kesehatan mental

Variabel Kontrol

- Wanita menopause yang sudah tidak mengalami menstruasi selama 12 bulan terakhir
- Kepatuhan terhadapa program penelitian

2. Deskipsi detil tentang desain penelitian (p12).
Penelitian ini menggunakan metode penelitian true experiment, dengan rancangan penelitian pretest-posttest control group design (Sugiyono, 2017) dalam skematis dapat digambarkan sebagai berikut :

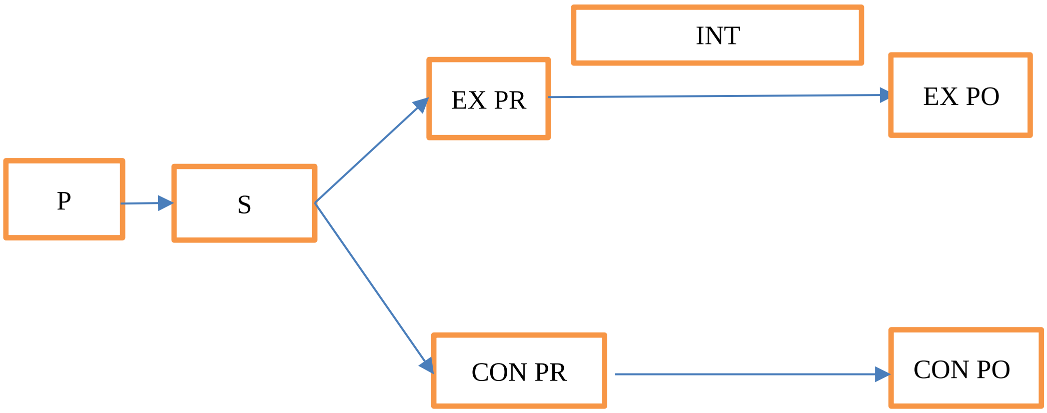

Keterangan:

P : Populasi.
S : Sampel.
EX PR : Pre-Test Kelompok Exercise.
EX PO : Post-Test Kelompok Exercise.
CON PR : Pre-Test Kelompok Kontrol.
CON PO : Post-Test Kelompok Kontrol.
INT : Intervensi Latihan Kombinasi 50-60 Menit, 5x/Minggu.

3. Bila uji coba klinis, deskripsi harus meliputi apakah kelompok *treatment* ditentukan secara random, (termasuk bagaimana metodenya), dan apakah *blinded* atau terbuka. (*Bila bukan uji coba klinis cukup tulis: tidak relevan) (p12)*

   Tidak relevan

F. Sampling

1. Penentuan besar sampel per kelompok dilakukan dengan menggunakan software G Power versi 3.1.9.7 dengan α=0.05, ß=0.8 berdasarkan data yang dipublikasikan Vaughan et al. (2014). Studi ini melaporkan ukuran efek 0,77 untuk tes Stroop (penamaan warna) sebagai hasil dari program latihan multimodal selama 4 minggu (terdiri dari latihan aerobik, kekuatan, keseimbangan, fleksibilitas, koordinasi dan kelincahan) terhadap kinerja kognitif pada wanita lansia. Analisis menunjukkan bahwa ukuran sampel yang diperlukan untuk menentukan signifikansi adalah minimal 14 subjek per kelompok.

2. Kriteria partisipan atau subjek dan justifikasi *exclude/include*. (*Guideline* 3) (p12)
   Kriteria inklusi dan eksklusi subjek pada penelitian ini adalah sebagai berikut:

   Kriteria inklusi subjek dalam penelitian ini adalah:
   - Berjenis kelamin perempuan.
   - Berusia minimal 50-80 tahun dan sudah menopause (≥12 bulan sejak menstruasi terakhir)
   - Pendidikan minimal Sekolah Menengah Pertama (SMP)

   Kriteria eksklusi subjek dalam penelitian ini adalah sebagai berikut:
   - Memiliki riwayat penyakit jantung koroner akut, stroke
   - Memiliki riwayat penyakit pendengaran dan penglihatan yang menganggu pelaksanaan test.
   - Memiliki riwayat penyakit muskuloskeletal yang menyebakan keterbatasan gerak.
   - Memiliki riwayat gangguan mental, dementia, atau gangguan kognitif sedang dan berat (skor MMSE < 21)

   Kriteria dropout subjek dalam penelitian ini adalah sebagai berikut:
   - Subjek mengikuti latihan ≤80% dari total latihan
   - Subjek tidak mampu melanjutkan proses pengambilan pos- test dan pre-test.

3. **Sampling kelompok rentan**: alasan melibatkan anak anak atau orang dewasa yang tidak mampu memberikan persetujuan setelah penjelasan, atau kelompok rentan, serta langkah langkah bagaimana meminimalisir bila terjadi risiko (*Guidelines* 15, 16 and 17) *(p15)*

   Tidak ada keterlibatan kelompok rentan dalam penelitian ini. Penelitian ini menggunakan subjek perempuan menopause 40-80 tahun.

G. Intervensi

(Pengguna data sekunder, kualitatif, cukup tulis tidak relevan, lanjut ke manfaat)

1. Deskripsi dan penjelasan semua intervensi (metode administrasi *treatment*, termasuk rute administrasi, dosis, interval dosis, dan masa *treatment* produk yang digunakan (investigasi dan komparator (p17).
   Latihan kombinasi dengan intensitas sedang yang terdiri atas latihan aerobik (briskwalking), resistance, dan balance dengan frekuensi 5x/minggu dengan durasi 60 menit/sesi selama 2 minggu. Penentuan intensitas latihan dengan menggunakan Rated Preceived Excertion (RPE) 5-6 pada skala Borg (1-10). Detail latihan terdapat pada lampiran

2. Rencana dan justifikasi untuk meneruskan atau menghentikan standar terapi selama penelitian (p 4 and 5) (p18)
   Intervensi latihan akan dihentikan apabila terdapat beberapa kriteria berikut ini dialami oleh subjek penelitian:
   Apabila subjek mengikuti latihan ≤80% dari total program latihan yang diberikan, subjek tidak dapat mengikuti proses pengukuran pretest dan posttest. Selain itu tim peneliti akan melakukan monitoring setiap minggu mengenai kondisi dan tanda vital subjek sehingga dapat menentukan apakah dapat diteruskan atau dihentikan.

3. *Treatment*/Pengobatan lain yang mungkin diberikan atau diperbolehkan, atau menjadi kontraindikasi, selama penelitian (p 6) (p19)
   Subjek dalam studi ini tidak melibatkan seseorang yang memiliki riwayat penyakit terentu sehingga tidak ada penggunaan obat lain yang digunakan atau diperbolehkan. Penggunaan vitamin, mineral diperbolehkan dan obat-obatan tertentu terkait penyakitnya diperbolehkan.

4. Tes klinis atau lab atau tes lain yang harus dilakukan (p20)
   Pada awal penelitian, individu yang berpotensi untuk menjadi partisipan pada penelitian ini akan diminta untuk mengisi kuesioer kesehatan. Setiap subjek akan menjalani pemeriksaan tekanan darah, denyut nadi sebanyak 2 kali, sebelum dan pada akhir penelitian.

H. Monitor Hasil

1. Sampel dari form laporan kasus yang sudah distandarisir, metode pencatatan respon terapeutik (deskripsi dan evaluasi metode dan frekuensi pengukuran), prosedur *follow-up*, dan, bila mungkin, ukuran yang diusulkan untuk menentukan tingkat kepatuhan subjek yang menerima *treatment* (lihat lampiran) (p17).

   Penelitian dilakukan intervensi selama 2 minggu dengan frekuensi latihan 5 kali seminggu. Pencatatan dilakukan pada awal dan akhir intervensi yang meliputih pengukuran fungsi kognitif dan stress psikologis, tensi, kuesioner kesehatan dan tingkat kepatuhan. Selama intervensi tim peneliti melakukan monitoring dengan melakukan pemeriksaan tanda vital dan pelaksanaan program agar subjek dapat menerima dan merasakan manfaat intervensi latihan.

I. Penghentian Penelitian dan Alasannya

1. Aturan atau kriteria kapan subjek bisa diberhentikan dari penelitian atau uji klinis, atau, dalam hal studi multi senter, kapan sebuah pusat/lembaga di non aktifkan, dan kapan penelitian bisa dihentikan (tidak lagi dilanjutkan) (p22)

   Intervensi latihan akan dihentikan apabila terdapat beberapa kriteria berikut ini dialami oleh subjek penelitian:
   Subjek penelitian dihentikan dari penelitian ini bila mengalami adverse event baik berhubungan dengan intervensi penelitian secara langsung maupun diluar intervensi.

J. *Adverse Event* dan Komplikasi (Kejadian Yang Tidak Diharapkan)

1. Metode pencatatan dan pelaporan *adverse events* atau reaksi, dan syarat penanganan komplikasi (*Guideline* 4 dan 23) (p.23)

   Pencatatan semua adverse event dilakukan di form terstandar di CRF (Case Report Form) masing-masing subjek penelitian. Peneliti memberikan nomor kontak untuk dapat dihubungi kapan saja bila muncul efek samping. Kejadian yang tidak diharapkan dilaporkan kepada asisten peneliti yang memonitor kondisi sampel penelitian secara kontinu, bila ditemukan kejadian yang tidak diharapkan peneliti bersama tenaga kesehatan (dokter) segara memberikan bantuan medis. Jika diperlukan perawatan lebih lanjut sampel dapat dirujuk ke rumah sakit.

2. Risiko risiko yang diketahui dari *adverse events*, termasuk risiko yang terkait dengan masing masing rencana intervensi, dan terkait dengan obat, vaksin, atau terhadap prosudur yang akan diuji cobakan (*Guideline* 4) (p24)

   Penelitian ini merupakan penelitian eksperimental dengan memberikan intervensi latihan. Risiko yang mungkin terjadi adalah reaksi kelelahan berlebihan. Risiko ini diminimalisir terjadi dengan melakukan monitoring rutin terhadap sample yang dilakukan pemeriksaan tanda vital dan kondisi subjek. Resiko yang mungkin dapat terjadi adalah timbulnya cedera akibat usia subjek yang tergolong dalam lansia sehingga harus dilakukan dengan hati hati dan sesuai prosedur yang tepat. Nmun hal ini sudah diantisipasi dengan penerapan intesitas yang tepat serta didampingi oleh personal trainer serta dokter.

K. Penanganan Komplikasi (p27)

Jika terjadi cedera saat melakukan intervensi latihan maka penanganan pertama cedera dilakukan dengan metode *Protection, Rest, Ice, Compression, dan Elevation* (PRICE). Jika kondisi komplikasi perlu adanya penanganan medis lebih lanjut ke rumah sakit, maka seluruh biaya perawatan akan ditanggung oleh peneliti, serta kompensasi terkait dengan disabilitas atau kematian akibat intervensi akan diberikan secara penuh oleh peneliti,

L. Manfaat

1. Manfaat penelitian secara pribadi bagi subjek dan bagi yang lainnya (*Guideline* 4) (p25)

   Dengan ini subjek bisa mengetahui efke baik dari intervensi yang diberikan dalam peningkatan fungsi kognitif sehingga kesehatan mental dari subjek akan membaik.

2. Manfaat penelitian bagi penduduk, termasuk pengetahuan baru yang kemungkinan dihasilkan oleh penelitian (*Guidelines* 1 and 4) (p26)

   Hasil penelitian ini diharapkan memberikan informasi kepada masyarakat bahwa latihan kombinasi mampu meningkatkan fungsi kognitif dan Kesehatan mental pada wanita menopause.

M. Jaminan Keberlanjutan Manfaat (p28)

Tidak ada jaminan keberlanjutan manfaat pada penelitian ini. Namun jika keberlangsungan intervensi latihan kombinasi ini memberi manfaat yang signifikan, maka olahraga ini dapat direkomendasikan bagi masyarakat khuusnya wanita yang memasuki masa menopause.

N. *Informed Consent*

1. Cara yang diusulkan untuk mendapatkan *informed consent* dan prosedur yang direncanakan untuk mengkomunikasikan informasi penelitian kepada calon subjek, termasuk nama dan posisi wali bagi yang tidak bisa memberikannya. (*Guideline* 9) (p30)

   Informed consent akan diberikan secara personal (tatap muka) ketika calon subjek telah mengisi data diri dan bersedia mengikuti penjelasan prosedur penelitian akan diberikan secara tertulis dan akan dijelaskan ulang apabila masih ada pertanyaan terkait dengan poin-poin yang belum dimengerti oleh calon subjek. Contoh *informed consent* dapat dilihat pada lampiran 1

2. Khusus Ibu Hamil: adanya perencanaan untuk memonitor kesehatan ibu dan kesehatan anak jangka pendek maupun jangka panjang (*Guideline* 19) (p29)

   Penelitian ini tidak mengikut sertakan ibu hamil sebagai subjek penelitian, melainkan hanya perempuan menopause yang digunakan sebagai subjek penelitian

O. Wali (p31)

1. Adanya wali yang berhak bila calon subjek tidak bisa memberikan *informed consent* (*Guidelines* 16 and 17)

   Tidak ada wali yang berhak memberikan informed consent dalam penelitian ini, karena subjek yang digunakan sudah dirasa dewasa serta bisa untuk memberikan keputusan sendiri berdasarkan hukum.

2. Adanya orang tua atau wali yang berhak bila anak paham tentang *informed consent* tapi belum cukup umur (*Guidelines* 16 and 17)

   Penelitian ini tidak mengikut sertakan anak-anak sebagai subjek penelitian.

P. Bujukan

1. Deskripsi bujukan atau insentif pada calon subjek untuk ikut berpartisipasi, seperti uang, hadiah, layanan gratis, atau yang lainnya (p32)

   Setiap subjek akan mendapatkan benefit pada akhir dari penelitian sejumlah uang dan cek kesehatan gratis. Subjek juga tidak dikenakan biaya apapun dalam pemeriksaan fisik dan kesehatan sehingga hasil dan konsultasi mengenai kondisi subjek diterima secara gratis.

2. Rencana dan prosedur, dan orang yang betanggung jawab untuk menginformasikan bahaya atau keuntungan peserta, atau tentang riset lain tentang topik yang sama, yang bisa mempengaruhi keberlansungan keterlibatan subjek dalam penelitian (*Guideline* 9) (p33)

   Semua informasi terkait prosedur penelitian, intervensi yang diberikan, bahaya atau keuntungan yang diperoleh akan disampaikan kepada subjek melalui form informed consent yang diberikan secara personal.

3. Perencanaan untuk menginformasikan hasil penelitian pada subjek atau partisipan (p34)

   Semua infromasi atau hasil yang telah diperoleh pada proses penelitian akan diberikan kepada subjek secara personal setelah semua data telah dilakukan analisa.

Q. Penjagaan Kerahasiaan

1. Proses rekrutmen (misalnya lewat iklan), serta langkah langkah untuk menjaga privasi dan kerahasiaan selama rekrutmen (*Guideline* 3) (p16)

   Proses rekrutment dilakukan melalui kelompok perkumpulan olahraga yang terdiri dari banyak wanita menopause dengan melakukan *screening* sesuai dengan kriteria inklusi penelitian. Semua data dalam penelitian ini akan dijaga kerahasiaannya dan hanya dipergunakan hanya untuk kepentingan penelitian semata.

2. Langkah langkah proteksi kerahasiaan data pribadi, dan penghormatan privasi orang, termasuk kehati-hatian untuk mencegah bocornya rahasia hasil test genetik pada keluarga kecuali atas izin dari yang bersangkutan (*Guidelines* 4, 11, 12 and 24) (p 35)

   Semua informasi yang ada dalam penelitian ini bersifat rahasia yang hanya diketahui oleh peneliti dan subjek dalam bentuk anonim

3. Informasi tentang bagaimana kode; bila ada, untuk identitas subjek dibuat, di mana di simpan dan kapan, bagaimana dan oleh siapa bisa dibuka bila terjadi emergensi (*Guidelines* 11 and 12) (p36)

Kode subjek berupa huruf (berdasarkan kelompok) dan angka (berdasarkan urutan subjek dalam kelompok). Kode identitas subjek hanya diketahui oleh peneliti dan disimpan pada device yang sudah dipersiapkan, serta akan dibuka apabila oleh salah satu tim peneliti yang diberikan tugas tersebut.

R. Kemungkinan penggunaan lebih jauh dari data personal atau material biologis (p37)

Belum ada rencana penggunaan data penelitian lebih jauh, tetapi jika data diperlukan peneliti akan meminta persetujuan sampel penelitian sebelum penggunaan

S. Rencana Analisis

1. Deskripsi tentang rencana tencana analisis statistik, termasuk rencana analisis interim bila diperlukan, dan kreteria bila atau dalam kondisi bagaimana akan terjadi penghentian prematur keseluruhan penelitian (*Guideline* 4) (B, S2)

   Terdapat beberapa tahapan dalam proses analisis data hingga dapat menjawab hipotesis yang telah di jelaskan, tahapan tersebut meliputi:

   - Semua analisis statistika yang dilakukan menggunakan PRISM versi 9.
   - Analisis deskriptif untuk menganalisis dan menggambarkan karakteristik subyek penelitian dan hasil pengukuran sebelum dan sesudah intervensi,
   - Uji beda menggunakan Repeated-measures ANOVA. Semua analisis statistika menggunakan taraf signifikan ($p<0.05$).

T. Monitor Keamanan

1. Rencana rencana untuk memonitor keberlangsungan keamanan obat atau intervensi lain yang dilakukan dalam penelitian atau trial, dan, bila diperlukan, pembentukan komite independen untuk data dan *safety monitoring* (*Guideline* 4) (B, S3, S7)

   Tingkat keamanan intervensi akan dipantau setiap minggu pada masa penelitian oleh tim peneliti yang terdiri atas tenaga medis. Setiap efek samping dari intervensi latihan akan ditangani sesuai dengan protokol penanganan jenis efek samping yang bersangkutan.

U. Konflik Kepentingan

1. Pengaturan untuk mengatasi konflik finansial atau yang lainnya yang bisa mempengaruhi keputusan para peneliti atau personil lainya; menginformasikan pada komite lembaga tentang adanya *conflict of interest*; komite mengkomunikasikannya ke komite etik dan kemudian mengkomunikasikan pada para peneliti tentang langkah langkah berikutnya yang harus dilakukan (*Guideline* 25) (p42)

   Tidak ada konflik kepentingan dalam penelitian ini.

V. Manfaat Sosial

1. Untuk riset yang dilakukan pada seting sumberdaya lemah, kontribusi yang dilakukan sponsor untuk *capacity building* untuk review ilmiah dan etika dan untuk riset-riset kesehatan di negara tersebut; dan jaminan bahwa tujuan *capacity building* adalah agar sesuai nilai dan harapan para partisipan dan komunitas tempat penelitian (*Guideline* 8) (p43)

   Tidak relevan

2. Protokol riset atau dokumen yang dikirim ke komite etik harus meliputi deskripsi rencana pelibatan komunitas, dan menunjukkan sumber sumber yang dialokasikan untuk aktivitas aktivitas pelibatan tersebut. Dokumen ini menjelaskan apa yang sudah dan yang akan dilakukan, kapan dan oleh siapa, untuk memastikan bahwa masyarakat dengan jelas terpetakan untuk memudahkan pelibatan mereka selama riset, untuk memastikan bahwa tujuan riset sesuai kebutuhan masyarakat dan diterima oleh

mereka. Bila perlu masyarakat harus dilibatkan dalam penyusunan protokol atau dokumen ini (*Guideline* 7) (p44)

Tidak ada keterlibatan komunitas masyarakat dalam riset ataupun penyusunan protokol penelitian ini.

W. Hak atas Data

1. Terutama bila sponsor adalah industri, kontrak yang menyatakan siapa pemilik hak publiksi hasil riset, dan kewajiban untuk menyiapkan bersama dan diberikan pada para PI draft laporan hasil riset (*Guideline* 24) (B dan H, S1, S7)

   Tidak relevan

X. Publikasi

1. Rencana publikasi hasil pada bidang tertentu (seperti epidemiologi, generik, sosiologi) yang bisa berisiko berlawanan dengan kemaslahatan komunitas, masyarakat, keluarga, etnik tertentu, dan meminimalisir risiko kemudharatan kelompok ini dengan selalu mempertahankan kerahasiaan data selama dan setelah penelitian, dan mempublikasi hasil hasil penelitian sedemikian rupa dengan selalu mempertimbangkan martabat dan kemuliaan mereka (*Guideline* 4) (p47)

   Hasil publikasi penelitian ini berhubungan dengan ilmu kesehatan olahraga yang tidak akan berlawanan dengan kemaslahatan komunitas, masyarakat, keluarga, etnik tertentu, dan meminimalisir resiko kemudharatan kelompok ini dengan selalu mempertahankan kerahasiaan data selama dan setelah penelitian, dan mempublikasi hasil hasil penelitian sedemikian rupa dengan selalu mempertimbangkan martabat dan kemulyaan subjek. Tidak ada perencanaan publikasi yang dapat beresiko dengan kemaslahatan komunitas, masyarakat, keluarga atau etnis tertentu dalam peneltian ini dan seluruh data akan dijamin kerahasiaanya.

2. Bila hasil riset negatif, memastikan bahwa hasilnya tersedia melalui publikasi atau dengan melaporkan ke otoritas pencatatan obat obatan (*Guideline* 24) (p46)

   Tetap menampilkan data sesuai yang terjadi di lapangan

Y. Pendanaan

Sumber dana riset dalam penelitian disponsori oleh Direktorat Jenderal Pendidikan Tinggi, Riset, dan Teknologi Kementerian Pendidikan, Kebudayaan, Riset, dan Teknologi berdasarkan Surat Keputusan Nomor 0536/E5/PG.02.00/2023 dan perjanjian kontrak Nomor 114/E5/PG.02.00.PL/2023; 1187/UN3.LPPM/PT.01.03/2023

Z. Komitmen Etik

1. Pernyataan peneliti utama bahwa prinsip-prinsip yang tertuang dalam pedoman ini akan dipatuhi (p6) Dengan sesungguhnya menyatakan bahwa saya bersedia mematuhi semua prinsip yang tertuang dalam pedoman etik KEMENKES. Seperti yang terlampir dalam pernyataan berikut: Menurut Kemenkes (2017) prinsip-prinsip etik dalam penelitian adalah sebagai berikut:

a. Respect For Pearsons Dalam penelelitian yang menjadikan manusia sebagai subjek manusia harus dihormati. Menghormati hak manusia untuk mengambil keptusan secara mandiri (selfdetermination), tidak dapat hidup sendiri (dependent) atau rentan (vulnerable) perlu diberikan perlindungan terhadap kerugian atau penyalahgunaan (harm and abuse).

b. Beneficence and Non Malefience Dalam penelitian seorang peneliti harus berbuat baik dan tidak merugikan manusia yang dijadikan sebagai objek penelitian. Prinsip tidak merugikan adalah jika tidak dapat melakukan hal yang bermanfaat, maka sebaiknya jangan merugikan orang lain. Prinsip etik berbuat baik, mempersyaratkan bahwa: 1) Risiko penelitian harus wajar (reasonable) dibanding manfaat yang diharapkan, 2) Desain penelitian harus memenuhi persyaratan ilmiah (scientifically sound), 3) Para peneliti mampu melaksanakan penelitian dan sekaligus mampu menjaga kesejahteraan subjek

penelitian, 4) Prinsip do no harm (tidak merugikan) yang menentang segala tindakan dengan sengaja merugikan subjek penelitian.

c. Justice Peneliti harus memperlakukan setiap orang (sebagai pribadi otonom) layak dalam memperoleh haknya. Prinsip etik keadilan terutama menyangkut keadilan yang merata (distributive justice) yang mempersyaratkan pembagian seimbang (equitable).

2. (*Track Record*) Riwayat usulan *review* protokol etik sebelumnya dan hasilnya (isi dengan judul dan tanggal penelitian, dan hasil *review* Komite Etik(p7)

   Peneliti belum pernah di Review untuk mendaptakan kelaikan etik

3. Pernyataan bahwa bila terdapat bukti adanya pemalsuan data akan ditangani sesuai *policy* sponsor untuk mengambil langkah yang diperlukan (p48)

   Apabila dikemudian hari ditemukan bukti adanya pemalsuan data, saya akan bersedia menerima sanksi yang telah ditentukan.

Tanda tangan Peneliti Utama
Surabaya, 1 Februari 2023

(Dani Rahmat Ramadhana)

AA. Daftar Pustaka

Chaturvedi, A., Nayak, G., Nayak, A. G., & Rao, A. (2016). Comparative assessment of the effects of hatha yoga and physical exercise on biochemical functions in perimenopausal women. *Journal of clinical and diagnostic research: JCDR*, *10*(8), KC01.Cutter, W. J., Norbury, R. & Murphy, D. G. 2003. Oestrogen, brain function, and neuropsychiatric disorders. *J Neurol Neurosurg Psychiatry,* 74**,** 837-40.

Erickson, K. I., Prakash, R. S., Voss, M. W., Chaddock, L., Heo, S., McLaren, M., ... & Kramer, A. F. (2010). Brain-derived neurotrophic factor is associated with age-related decline in hippocampal volume. *Journal of Neuroscience*, *30*(15), 5368-5375.Grindler, N. M. & Santoro, N. F. 2015. Menopause and exercise. *Menopause,* 22**,** 1351-8.

Kravitz, H. M., Zhao, X., Bromberger, J. T., Gold, E. B., Hall, M. H., Matthews, K. A., & Sowers, M. R. (2008). Sleep disturbance during the menopausal transition in a multi-ethnic community sample of women. *Sleep*, *31*(7), 979-990.

Mulyani, S. (2013). Menopause akhir siklus menstruasi pada wanita di usia pertengahan. *Yogyakarta: Nuha Medika*, *10*.

Pavone, M. E., & Bulun, S. E. (2012). Aromatase inhibitors for the treatment of endometriosis. *Fertility and sterility*, *98*(6), 1370-1379.

Sasnitiari, S. M. N. N. (2018). Pengaruh Senam Aerobik Low Impac terhadap Pengurangan Keluhan Ibu Premenopause di Wilayah Puskesmas Merdeka Bogor Tahun 2016. *Jurnal Bidan*, *4*(1), 234015..

Sulisetyawati, S. D. (2011). Dampak Menopause Terhadap Konsep Diri Wanita yang Mengalami Menopause di Kelurahan Trengguli Kecamatan Jenawi Kabupaten Karanganyar. *Jurnal Kesehatan Kusuma Husada*.

Thurston, R. C., Sutton-Tyrrell, K., Everson-Rose, S. A., Hess, R., Powell, L. H., & Matthews, K. A. (2011). Hot flashes and carotid intima media thickness among midlife women. *Menopause (New York, NY)*, *18*(4), 352.

Vaughan, S., Wallis, M., Polit, D., Steele, M., Shum, D., & Morris, N. (2014). The effects of multimodal exercise on cognitive and physical functioning and brain-derived neurotrophic factor in older women: a randomised controlled trial. *Age and ageing*, *43*(5), 623-629.Vincent, A., Riggs, B. L., Atkinson, E. J., Oberg, A. L. & Khosla, S. 2003. Effect of estrogen replacement therapy on parathyroid hormone secretion in elderly postmenopausal women. *Menopause,* 10**,** 165-71.

Whitmer, R. A., Quesenberry, C. P., Zhou, J., & Yaffe, K. (2011). Timing of hormone therapy and dementia: the critical window theory revisited. *Annals of neurology*, *69*(1), 163-169.

WHO, World Health Organization 2014. Social Determinants of Health in Menopause.

Wulandari, R. C. L. (2016). Terapi sulih hormon alami untuk menopause. *INVOLUSI Jurnal Ilmu Kebidanan*, *5*(10).

AB.Lampiran

A. INFORMED CONSENT

INFORMED CONSENT
(PERNYATAAN PERSETUJUAN IKUT PENELITIAN)

Yang bertanda tangan dibawah ini :
Nama :
Umur :
Jenis Kelamin :
Pekerjaan :
Alamat :

Telah mendapat keterangan secara terinci dan jelas mengenai :

1. Penelitian yang berjudul “Latihan kombinasi aerobic (jalan cepat), kekuatan dan keseimbangan terhadapa fungsi kognitif dan kesehatan mental pada wanita menopause”.
2. Perlakuan yang akan diterapkan pada subyek
3. Manfaat ikut sebagai subyek penelitian
4. Bahaya yang akan timbul
5. Prosedur Penelitian

dan prosedur penelitian mendapat kesempatan mengajukan pertanyaan mengenai segala sesuatu yang berhubungan dengan penelitian tersebut. Oleh karena itu saya bersedia/tidak bersedia*) secara sukarela untuk menjadi subyek penelitian dengan penuh kesadaran serta tanpa keterpaksaan.
Demikian pernyataan ini saya buat dengan sebenarnya tanpa tekanan dari pihak manapun.

Surabaya,

Peneliti, Responden,

(Dani Rahmat Ramadhana) ……………………………

Saksi,

…………………………………………

*) Coret salah satu

B. PROGRAM LATIHAN

Komponen latihan	Jenis latihan	Contoh latihan	Intensitas	Durasi
**Minggu ke-1**				
Aerobik	Jalan cepat	Berbaris di tempat (dengan latihan lengan), angkat lutut tunggal, langkah satu sisi, ketuk tunggal, langkah silang, langkah maju / mundur, tendangan	5-6/10 RPE	10 menit
Kekuatan	Kelompok otot bsar tubuh dengan menggunakan *loop band*	*Band biceps curl, ekstensi triceps Band, Band chest press, Band shoulder press, Band bridge abs, Band Squat, Band leg press, lateral band walk*	2 set, 6 repetisi	10 menit
Keseimbangan	Keseimbangan statis dengan bantuan dua lengan	Berdiri dengan kaki berdampingan, berjalan dengan ujung jari kaki menyetuh tumit kaki sebelahnya, berdiri satu kaki, jinjit dengan menggunakan ujung kaki dan tumit secara bergantian	N/A	10 menit
Fleksibilitas dan pendinginan	Peregangan statis	Peregangan leher, peregangan lengan, peregangan bahu, peregangan punggung, peregangan pinggul, peregangan kaki, *butterfly stretch*, dan *cat and camel*	2 set, 6 hitungan	10 menit
			Total waktu	40 menit
**Minggu ke-2**				
Aerobik	Jalan cepat	Berbaris di tempat (dengan latihan lengan), angkat lutut tunggal / ganda, langkah sisi tunggal / ganda, ketukan tunggal / ganda, langkah silang, langkah maju / mundur, tendangan	5-6/10 RPE	20 menit
Kekuatan	Kelompok otot bsar tubuh dengan menggunakan *loop band*	*Band biceps curl, ekstensi triceps Band, Band chest press, Band shoulder press, Band bridge abs, Band Squat, Band leg press, lateral band walk*	2 set 8 repetisi	15 menit
Keseimbangan	Keseimbangan statis tanpa dukungan lengan	Berdiri dengan kaki berdampingan, berjalan dengan ujung jari kaki menyetuh tumit kaki sebelahnya, berdiri satu kaki, jinjit dengan menggunakan ujung kaki dan tumit secara bergantian	N/A	10 menit
Fleksibilitas Peregangan dan pendinginan	Peregangan statis	Peregangan leher, peregangan lengan, peregangan bahu, peregangan punggung, peregangan pinggul, peregangan kaki, *butterfly stretch*, dan *cat and camel*	2 set, 8 hitungan	15 menit
			Total waktu	60 menit

** Urutan nomor pada Protokol Asli CIOMS 2016*
