# Supplementary material for: Short-term multicomponent exercise training improves executive function in postmenopausal women
Source: PLoS One. 2024 Aug 14;19(8):e0307812. doi: 10.1371/journal.pone.0307812 (PMC11324113; doi:10.1371/journal.pone.0307812)
Supplement: S2 File — (PDF) [file pone.0307812.s004.pdf]

Health-related Research Ethics Protocol
Involving Humans as Subjects

Fill in the form below with a brief description and tick (X/V) in the box or circle one of the chosen answer that describes the research.<br>P: CIOMS 2016 Protocol Sequence Number – Attachment 1;<br>S: Ethical Compliance Standards (WHO-2011 and KEPPKN Guidelines 2017);<br>C: Check List/Viewlist<br>M: CIOMS 2016 Guideline<br>IC: CIOMS 2016 – Appendix 2

List of contents:

A. Title of the research (p-protocol no 1)*
B. Summary of research proposal (p-protocol no 2)
C. Ethical issues raised by the research
D. Summary of literature review
E. Description of the site(s)
F. Research design
G. Sampling
H. Intervention
I. Results monitoring
J. Termination of research and reasons
K. Adverse Events and Complications (Unexpected Events)
L. Management of complication
M. Benefit
N. Guarantee of continuity of benefits
O. Informed consent
P. Legally authorized representative
Q. Inducements
R. Protecting the confidentiality
S. Plans for statistical analysis
T. Safety monitoring
U. Conflicts of interests
V. Benefits to the population or community
W. Data rights
X. Publication
Y. Funding
Z. Ethical commitment
AA. Bibliography
AB. Appendix
1. Curiculum vitae of principal investigator
2. Sample case report form

Health-related Research Ethics Protocol
Involving Humans as Subjects

Fill in the form below with a brief description and tick (X/V) in the box or circle one of the chosen answer that describes the research.
P: CIOMS 2016 Protocol Sequence Number – Attachment 1;
S: Ethical Compliance Standards (WHO-2011 and KEPPKN Guidelines 2017);
C: Check List/Viewlist
M: CIOMS 2016 Guideline
IC: CIOMS 2016 – Appendix 2

A. Title of the research (p-protocol no 1)*

Effects of combination aerobic exercise (brisk walking), strength and balance on cognitive function and mental health in menopausal women.

1. Research sites:
   Thor Field, Surabaya

2. Planned research time (start – finish)
   February 2023- February 2024

	Yes	No
3. Is this study multi-center		√
4. If it is multi-center, has it received ethical approval from other centers/institutions (attach if so)		√

Identification (p10)

1. Researcher
   (Please attach the CV of Principal Investigator)
   Principal Investigator (PI) : Dani Rahmat Ramadhana., S.Pd
   Institution : Faculty of Medicine, Airlangga University
2. Research Member : Raden Argarini dr., M.Kes., Ph.D
   Institution : Faculty of Medicine, Airlangga University
3. Sponsors (p9)
   Name :-
   Address :-

B. Summary of research proposal (p-protocol no 2)

1. Summary in 200-300 words (written in lay language not for doctors/professionals)

   Menopause is a natural process in the reproductive cycle of women, occurring alongside puberty, pregnancy, and menstruation. This condition is characterized by the absence of menstruation and marks the end of the fertility period. During menopause, women experience signs and symptoms due to decreased estrogen levels, such as vasomotor instability, genital atrophy, and mood disorders. Postmenopausal women also experience cognitive disorders, such as decreased memory or memory deficits, impaired concentration, and changes in mood and behavior. These symptoms lead to a decrease in quality of life and discomfort in performing daily activities. Physical exercise is one way to enhance cognitive function in postmenopausal women. Physical exercise that combines various types of exercises is believed to have the potential to yield greater improvements in cognitive function compared to single exercises because it stimulates different areas of the brain. This study aims to analyze the effect of a short-term combined exercise program on cognitive function in postmenopausal women. Subjects in this study were women aged 50-80 years who were at least 12 months post-menopause and had a minimum education level of junior high school or equivalent. Research subjects will be recruited from the Family Welfare Empowerment (PKK) program groups. Subjects were then allocated into a control group and an exercise intervention group. Subjects in the exercise group will perform a combination of exercises comprised of aerobic, strength, flexibility, and balance at moderate intensity. Exercise will be performed five times a week for two weeks. In the first week, each session lasts 40 minutes and gradually increases to 60 minutes. Cognitive function was assessed using the Stroop test to evaluate executive function and the Mini-Mental State Examination (MMSE) for global cognitive function. In addition, psychological stress will be measured using the Depression Anxiety Stress Scale-21 (DASS-21).

2. Research justification (p3). Write down why this research should be carried out, its significance in development and in meeting the needs of population in which the research is carred out(Country, region, local)- Standard 2/A (Fairness)

   In 2030, the global number of women reaching menopause is projected to reach 1.2 billion (WHO, 2014). As women's life expectancy increases in Indonesia, a growing number of women are projected to spend a significant portion of their lives in the menopausal phase, with an estimated 60 million reaching this stage by 2025 (WHO, 2014). In Indonesia, menopause typically occurs between the ages of 44 and 45, which is earlier than in developed countries (over 47 years). As menopausal women age, there is a simultaneous increase in health issues related to menopause. To the best of our knowledge, there has been a lack of research examining the impact of a combination of aerobic exercise (such as brisk walking), strength training, and balance exercises on cognitive function and mental health in menopausal women. Consequently, this study aims to investigate the effects of physical exercise, including a combination of aerobic exercise (fast walking), muscle-strengthening exercises, and balance exercises, on cognitive function and mental health in post-menopausal women.

C. Ethical issues raised by the research

1. The investigators view of ethical issues and consideration raised in this research and how it is proposed to deal with them (p4) – conform to the 7 points of ethical standards (S) and G.

   This study will be conducted on post-menopausal women participants categorized in the elderly age group. The elderly are more vulnerable to injuries, including musculoskeletal and cardiorespiratory issues. However, this susceptibility can be reduced by implementing the appropriate exercise intensity, under the guidance of a personal trainer and medical supervision. In the event of an injury, the initial treatment follows the Protection, Rest, Ice, Compression, and Elevation (PRICE) method.

D. Summary of literature review

1. A summary of all previous studies according to the research topic, including unpublished studies known to the investigators and sponsors, and information on previously published research, including if there are relevance animal studies. Maximum 1 p (p5)- G 4

Menopause is the natural cessation of menstruation that typically occurs in women between the ages of 45 and 55, but this timing can vary significantly and is influenced by various physiological and psychological factors (Chaturvedi et al., 2016). Menopausal women often experience cognitive disorders, including decreased memory, impaired concentration, and changes in mood and behavior (Whitmer et al., 2011). These symptoms can lead to a decline in the quality of life and discomfort during daily activities (Thurston et al., 2011; Discigil et al., 2006). Estrogen, acting as a neuroprotective agent, plays a crucial role in the function of the ventral hippocampus. A decrease in estrogen levels during menopause can contribute to memory and cognitive disorders in women at this stage (Cutter et al., 2003). Additionally, changes in memory and cognitive function in postmenopausal women are associated with decreased expression of Brain-Derived Neurotrophic Factor (BDNF) in the hippocampus and cerebral cortex (Erickson et al., 2010).

Physical exercise emerges as a potentially effective method for preventing or reducing cognitive impairment in postmenopausal women. A cross-sectional observational study on menopausal women demonstrated that low-impact aerobic exercise had a positive effect in reducing vasomotor complaints, psychological issues, and somatic complaints associated with menopause (Sasnitiari, 2018). Enhanced quality of life is closely linked to improved cognitive function. Therefore, both aerobic and strength training are linked to enhancements in cognitive functions such as executive function, inhibitory control, and episodic memory (Liu & Eden, 2008). The neuroprotective effects of exercise may be facilitated by the exercise-induced increases in neurotrophic factors, such as BDNF, insulin-like growth factor type I (IGF-1), vascular endothelial growth factor (VEGF), and homocysteine (Vincent et al., 2003). These factors cause structural and connectivity changes in brain regions essential for improving memory and cognitive function, including the frontal and temporal lobes and the hippocampus (Erickson et al., 2010). However, currently, there is a lack of research examining the impact of a combination of aerobic exercise (such as brisk walking), strength training, and balance exercises on cognitive function and quality of life in menopausal women.

E. Description of the site(s)

1. Brief description of the site(s) where the research to be conducted (p8) see G-2

   The research will be conducted in Thor Field, Surabaya. This area has outdoor and indoor courts, along with some rooms that can be used to conduct the assessment for the outcomes of this study.

2. Information about the adequacy of the availability of facilities for the safe and appropriate conduct the research,

   The assessment of blood pressure and pulse is conducted in a dedicated room at the training site, along with health questionnaires. The standardized instruments are available for use at the Department of Physiology and Medical Biochemistry, Faculty of Medicine, Airlangga University. The required variables will be measured by trained personnel according to a predetermined schedule, initially before implementing the exercise intervention program and again after two weeks of implementing the program. The research will be conducted in accordance with applicable health protocols.

3. Relevant demographic/epidemiological information about the region concerned.

   With a population of around 3,052,020 people in 2017, the city of Surabaya is evolving into a metropolitan city. The strategic location of Surabaya as the center of community economic activity keeps it in a constant state of dynamism. Surabaya has a tropical climate, similar to that of other major cities in Indonesia. According to the Köppen climate classification, Surabaya is categorized as having a tropical wet and dry climate (Aw) with two distinct seasons: the rainy season and the dry season. The

average annual rainfall in Surabaya is 165.3 mm, with the highest rainfall, exceeding 200 mm, occurring in January to March and November to December. The average air temperature in Surabaya ranges from 23.6°C to 33.8°C. The research will be conducted in the community hall at room temperature.

F. Research design

1. Research objectives, hypotheses, research questions, assumptions and research variables (p11).

   Research Question:
   - Does short-term combined exercise traning improve cognitive function and mental health in postmenopausal women?

   Research Objectives:
   - To demonstrate that short-term combined exercise training can improve cognitive function and mental health in menopausal women.

   Hypotheses:
   - Short-term combined exercise training can improve cognitive function and mental health in postmenopausal women.

   Research Variables:
   Independent Variable
   - Short-term combination exercise training

   Dependent Variables
   - Executive Function
   - Global kognitive function
   - Mental health

   Control Variables
   - Menopausal women who have not had menstruation for the last 12 months
   - adherence to exercise program,

2. Detailed description of the research design (p12).
   The design of this research is a pretest-posttest control group design (Sugiyono, 2017) which can be schematically described as follows:

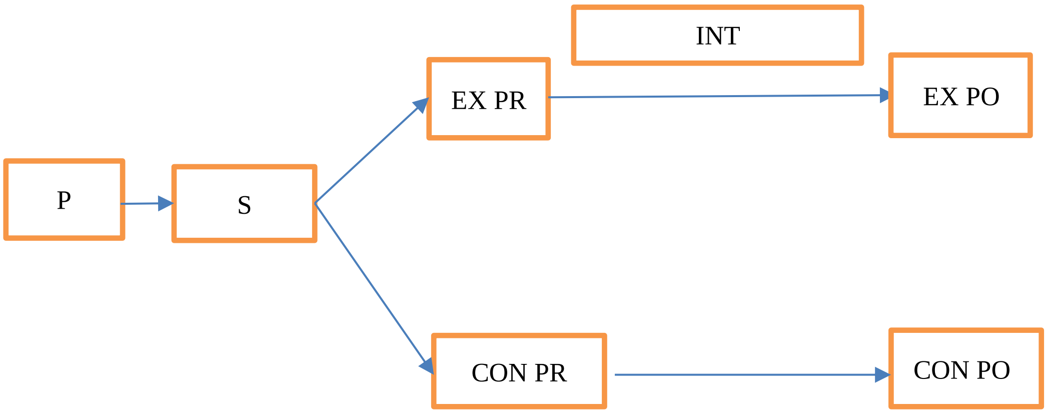

Information:
P :Population.
S : Sample.
EX PR : Exercise Group Pre-Test.
EX PO : Exercise Group Post-Test.

CON PR : Group Pre-TestControl.
CON PO : Group Post-TestControl.
INT :Combination Exercise Intervention 40-60 Minutes, 5x/Week.

3. If it is a clinical trial, the description should include, but not limited to, whether the assignment to treatment groups will be randomized (including the method of randomization), and whether the study will be blinded (single blind, double blind) or open. (If not a clinical trial just write: not relevant) (p12)

   Not relevant

G. Sampling

1. The determination of the sample size per group was performed using G Power software version 3.1.9.7 with α=0.05, ß=0.8 based on the published data of Vaughan *et al.* (2014). This study reported an effect size of 0.77 for the Stroop test (colour naming) as a result of a 16-week multimodal exercise program (including aerobic, strength, balance, flexibility, coordination, and agility exercises) on cognitive performance in older women. The analysis indicated that the required sample size to establish significance is 14 per group.

2. Criteria for participants or subjects and justification for exclude/include. (Guideline 3) (p12)
   The detail of inclusion and exclusion criteria are as follows:
   Inclusion Criteria:
   - Women aged 50-80 years old who are at least 12 months postmenopausal
   - Minimum education level is Junior High School (SMP).

   Exclusion Criteria:
   - Have a history of stroke, recent cardiovascular diseases (such as ischemic heart diseases and heart failure)
   - Had severe hearing or visual impairment that can disturb the assessment in this study.
   - Have a history of or any neuromuskuloskeletal problems, recent surgery
   - Had mental health problems, dementia or moderate to severe cognitive impairment (defined as score of less than 21 on the MMSE

   Dropout Criteria:
   - Subjects who participated in ≤80% of the total training sessions.
   - Subjects unable to continue the process of taking the post-test and pre-test

3. **Sampling of vulnerable persons or groups**: reasons for involving children or adults who are unable to give informed consent, or vulnerable persons or groups, as well as a description of special measures to minimize risks to such persons (Guidelines 15, 16 and 17) (p15)
   There is no involvement of vulnerable groups in this research. The study included post-menopausal women between the ages of 50-80.

H. Intervention

(Research using secondary or qualitative data, please write irrelevant, go to benefits)

1. Description and explanation of all interventions (treatment administration methods, including administration route, dosage, dosage interval, and treatment period of the product used (investigation and comparator (p17).

   Moderate-intensity combination training includes aerobic activities (such as brisk walking), resistance training, and balance exercises. The training frequency is set at 5 sessions per week, with each session lasting up to 60 minutes, conducted over a 2-week period. The determination of exercise intensity is based on the Rated Perceived Exertion (RPE) scale, targeting a range of 5-6 on the Borg scale (1-10). For further information, please refer to the attached document for specific exercise details.

2. Plan and justification for continuing or discontinuing standard therapy during the study (p 4 and 5) (p18)

   The exercise intervention will be discontinued if the research subject meets any of the following criteria:
   - The subject participates in < 80% of the total training program provided.
   - The subject is unable to partake in the pretest and posttest measurement process.

   Furthermore, the research team will regularly monitor the subject's condition and vital signs weekly. This ongoing assessment will help determine whether the intervention can be continued or should be halted.

3. *Treatment*/Other treatments that may be given or permitted, or be contraindicated, during the study (p 6) (p19)

   The participants in this study did not include individuals with a history of specific diseases, and as a result, no other medications were administered or permitted. The use of vitamins, minerals, and certain medications related to the existing condition is allowed.

4. Clinical or laboratory tests or other tests that must be carried out (p20)
   At the beginning of the study, participant will require to complete a health questionnaire for screening. Each participant will have their blood pressure and pulse measured on two occasions.

I. Results monitoring

1. Samples of standardized case report forms, methods of recording therapeutic responses (description and evaluation of methods and frequency of measurement), follow-up procedures, and, where possible, proposed measures to determine the level of compliance of subjects receiving treatment (see appendix) (p17).

   The research will be conducted as a two-week intervention, with a training frequency of five times per week. Data recording will take place at the beginning and end of the intervention, including assessments of cognitive function and mental health, blood pressure measurements, completion of health questionnaires, and evaluations of adherence levels. Throughout the intervention, the research team monitored the participants by regularly checking vital signs and implementing the program to ensure that subjects could benefit from and engage in the exercise.

J. Termination of research and reasons

1. Methods or criteria for when subjects can be dismissed from research or clinical trials, or, in the case of multi-centre studies, when a center/institution is deactivated, and when research can be stopped (no longer continued) (p22)
   The training intervention will be discontinued if the following criteria are met by the research subject: Research subjects will be withdrawn from the study if they experience an adverse event directly related to the research intervention or outside of the intervention.

K. Adverse events and complications (Unexpected events)

1. Methods for recording and reporting adverse events or reactions, and provision for dealig with complications (Guideline 4 and 23) (p.23)
   All adverse events are recorded on a standardized form in the CRF (Case Report Form) for each research partcipant. Researchers provide a contact number, allowing them to be reached at any time if side effects occur. Unexpected events are reported to the research assistant who consistently monitors the condition of the research sample. In the event of an unexpected occurrence, the researcher and the healthcare professional (doctor) promptly provide medical assistance. If additional treatment is necessary, the sample may be referred to a hospital.

2. The known or foreseen risks of adverse reactions, including the risks attached to each proposed intervention, and to any drug, vaccine, or procedure to be tested (Guideline 4) (p24)

This research is experimental and involves exercise intervention. The potential risk that may arise is an excessive fatigue response. To minimize this risk, regular monitoring of the samples is conducted by checking vital signs and assessing the subjects' condition. Another potential risk is the occurrence of injuries among elderly subjects. Consequently, precautions are taken, and procedures are carefully followed. However, this has been mitigated by applying the appropriate intensity and ensuring the presence of a personal trainer and doctor for supervision.

L. Management of the complications (p27)

If an injury occurs during the training intervention, the initial treatment for the injury follows the Protection, Rest, Ice, Compression, and Elevation (PRICE) method. If complications require further medical treatment at the hospital, all related treatment expenses will be covered by the researcher. Full compensation related to disability or death resulting from the intervention will also be provided by the researcher.

M. Benefit

1. The potential individual benefits of research for the participants and for others (Guideline 4) (p25)

   Through this study, the subject can recognize the positive effects of the intervention on enhancing cognitive function, ultimately leading to an improvement in the subject's mental health.

2. The expected benefits of research to the population, including the new knowledge that the research might generate (Guidelines 1 and 4) (p26)

   It is anticipated that the outcomes of this research will provide insights to the public, indicating that combined exercise can improve cognitive function and mental health in menopausal women.

N. Guarantee of continuity of benefits (p28)

There is no guarantee of long-term benefits from this research. Nevertheless, if the continuation of this combined exercise intervention yields significant benefits, it could be recommended, especially for individuals, particularly women entering menopause.

O. Informed consent

1. The means proposed to obtain individual informed consent and the procedures planned to communicate information to prospective participants, including the name and position of the person responsible for obtaining consent for those who cannot provide it. (Guideline 9) (p30)

   Informed consent will be provided in person (face to face) after the prospective subject has completed personal information and expresses willingness to understand the written explanation of research procedures. The details will be further clarified if the prospective subject has any questions or points that they do not comprehend. An example of the informed consent form can be found in Appendix 1.

2. For research on pregnant women: a plan, if appropriate, for monitor the outcome of the pregnancy with regards to both health of the woman and and the short-term and long-term health of the child (Guideline 19) (p29)

   This research do not involve pregnant women as research subjects.

P. Legally authorized representattive (p31)

1. When the prospective paricipants is not capable of informed consent, satisfactory assurance that permission will be obtained from a duly authorized person. (Guidelines 16 and 17)

   A guardian or other authorized person is required to provide informed consent for this research, as the subjects are legally recognized as adults and are capable of making their own decisions.

2. In the case of a child who is sufficiently mature to understand the implication of informed consent but has not reach the legal age for consent, that knowing agreement, or assent, will be obtained , as well as permission from parent, or legal guardian or other duly authorized representatives (Guidelines 16 and 17)

   This research does not include children as research subjects.

Q. Inducements

1. Description of inducements or incentives for prospective participants, such as money, prizes, free services, or others (p32)

   Each participant will receive compensation at the end of the study, including a monetary payment and a free health check-up. Subjects will not incur any fees for physical and health examinations, ensuring that results and consultations regarding the subjects' conditions are provided free of charge.

2. Plans and procedures, and the person responsible for informing participants of harm or benefit, or about other research on the same topic, which may affect the subject's continued involvement in research (Guideline 9) (p33)

   All information regarding research procedures, interventions provided, potential risks or benefits, and any other research on the same topic that may impact the subject's ongoing participation will be communicated to the subject through a personally delivered informed consent form.

3. Plans to inform research results to subjects or participants (p34)

   All information or results obtained during the research process will be personally communicated to the participants after the completion of data analysis.

R. Protecting the confidentiality

1. Recruitment process (for example through advertising), as well as measures to ensure repects for the privacy of participants and confidentiality during recruitment (Guideline 3) (p16)

   The recruitment process was conducted through family welfare comprising numerous menopausal women who underwent screening according to the research inclusion criteria. All data in this research will be treated confidentially and used exclusively for research purposes.

2. Measures to protect the confidentiality of individual data, and ensure repects for the privacy of participants, including taking care to prevent the leaking of confidential genetic test results to the family unless with permission from the person concerned (Guidelines 4, 11, 12 and 24)(p 35)

   All information in this research is confidential and known only to the researcher and the subject in an anonymous form.

3. Information about how to code; if there is, for the identity of the subject to be created, where it is stored and when, how and by whom it can be opened if an emergency occurs (Guidelines 11 and 12) (p36)

Subject codes consist of letters (based on groups) and numbers (based on the order of subjects in groups). The subject's identity code is only known to the researcher and is stored on the prepared device, to be opened when assigned to one of the research team members.

S. Possibility of further use of personal data or biological material (p37)

There are no plans for further use of research data, but if the data is needed, researchers will ask for participant's approval before use

T. Plans for statistical analysis

1. Description of the plans for statistical analysis of the research, including plans for interim analysis if any, and criteria for prematurely terminating the entire research if necessary (Guideline 4) (B, S2)

   The statistical analyses will be performed using PRISM software version 9. Descriptive analysis will be utilized to examine and describe the characteristics of research subjects and measurement results before and after intervention. The comparison test will employ a Repeated-measures ANOVA. All statistical analyses use a significance level of $p<0.05$.

U. Safety monitoring

1. Plans for monitoring the continuing safety of drugs or other interventions administered for purposes of the study or trial and, if appropriate, the appointment for this purpose of an independent for data and safety monitoring committee (Guideline 4) (B, S3, S7)

   The safety of the intervention will be monitored weekly during the research period by a team of medical professionals. Any side effects resulting from the exercise intervention will be addressed in accordance with the protocol for managing the specific type of side effect.

V. Conflicts of interests

1. Arrangements to resolve financial or other conflicts that could influence the decisions of researchers or other personnel; informing the institutional committee about the existence of a conflict of interest; the committee communicates it to the ethics committee and then communicates to the researchers about the next steps that must be taken (Guideline 25) (p42)

   There is no conflict of interest in this research.

W. Benefits to the population or community

1. For research carried out in low-resource settings, the contribution made by the sponsor should include capacity building for scientific and ethical review and for health research in the country; and assurance that the purpose of capacity building is to match the values and expectations of the participants and the research community (Guideline 8) (p43)

   Irrelevant

2. The research protocol or document sent to the ethics committee should include a description of the community engagement plan, and indicate the resources allocated to the engagement activities. This document explains what has been and will be done, when and by whom, to ensure that communities are clearly mapped out to facilitate their involvement during research, to ensure that research objectives meet community needs and are accepted by them. If necessary, the community must be involved in preparing this protocol or document (Guideline 7) (p44)

There was no community involvement in the research or preparation of this research protocol.

X. Data rights

1. In the case of industrial sponsor, a contract stipulating who posesses the right to publish the results of research, and a mandatory obligation to prepare with, and submit to, the principal investigators the draft of the text reporting the results (Guideline 24) (B and H, S1, S7)

   Irrelevant

Y. Publication

1. Plan for publication of research results in certain fields (for example epidemiology, generics, sociology) that may presents to the interest of risk of being contrary to the benefit of a particular community, society, family, ethnicity, and for minimizing risks to thess group, notably by maintaining data confidentiality during and after the research, and publishing the resulting data in such a way that always respectful of the interest of all concerned (Guideline 4) (p47)

   The results of this research publication are related to sports health science, and they will not conflict with the benefit of certain communities, society, families, or ethnic groups. The research aims to minimize the risk of harm to these groups by always maintaining data confidentiality during and after the research, and by publishing research results in a manner that always takes into account the dignity and nobility of the subject. No publication plan would jeopardize the well-being of any specific community, society, family, or ethnicity in this research, and all data will be kept confidential.

2. In the case of negative outcome, an assurance that the results will be made available as appropriate through publication or by reporting to the drug registration authority (Guideline 24) (p46)

   We will present the data according to the results of the research.

Z. Funding

This research is funded by the Directorate General of Higher Education, Research, and Technology - Ministry of Education, Culture, Research, and Technology based on Decree Number 0536/E5/PG.02.00/2023 and contract agreement Number 114/E5/PG.02.00.PL/2023; 1187/UN3.LPPM/PT.01.03/2023.

AA. Ethical Commitment

1. The statement of principal investigator that the principles set out in these guidelines will be adhered to (p6)
   I truly declare that I am willing to comply with all the principles outlined in the KEMENKES ethical guidelines. As attached in the following statement: According to the Ministry of Health (2017) the ethical principles in research are as follows:

a. Respect for Persons: In research involving human subjects, it is essential to respect their rights and dignity. Respecting individuals' rights to make decisions independently (self-determination), those unable to live alone (dependent), or vulnerable (vulnerable) require protection against harm and abuse.
b. Beneficence and Non-Maleficence: In research, researchers must ensure that they do not cause harm to the human subjects involved and strive to do good. The principle of doing no harm is that if you cannot do something useful, then you should not harm other people. The ethical principle of beneficence requires that: 1) Research risks must be reasonable compared to the expected benefits, 2) Research design must meet scientific requirements (scientifically sound), 3) Researchers are able to carry out research and at the same time maintain the welfare of research subjects, 4) The principle of non-maleficence (do not harm) opposes all actions that intentionally harm research subjects.
c. Justice. Researchers must treat each person as an autonomous individual worthy of obtaining their rights. The ethical principles of justice primarily concern distributive justice, which requires fair and balanced distribution.

2. (Track Record) History of previous ethical protocol review proposals and their results (fill in the title and date of the research, and the results of the Ethics Committee review (p7)

   Researchers have never been evaluated for ethical suitability.

3. A statement that if there is evidence of data falsification it will be handled according to the sponsor's policy to take the necessary steps (p48)

   If evidence of data falsification is found in the future, I will be willing to accept the specified sanctions.

Principal Investigator's signature
Surabaya, February 1 2023

(Dani Rahmat Ramadhana)

BB. Bibliography

Chaturvedi, A., Nayak, G., Nayak, A. G., & Rao, A. (2016). Comparative assessment of the effects of hatha yoga and physical exercise on biochemical functions in perimenopausal women. *Journal of clinical and diagnostic research: JCDR*, *10*(8), KC01.Cutter, W. J., Norbury, R. & Murphy, D. G. 2003. Oestrogen, brain function, and neuropsychiatric disorders. *J Neurol Neurosurg Psychiatry,* 74**,** 837-40.

Erickson, K. I., Prakash, R. S., Voss, M. W., Chaddock, L., Heo, S., McLaren, M., ... & Kramer, A. F. (2010). Brain-derived neurotrophic factor is associated with age-related decline in hippocampal volume. *Journal of Neuroscience*, *30*(15), 5368-5375.Grindler, N. M. & Santoro, N. F. 2015. Menopause and exercise. *Menopause,* 22**,** 1351-8.

Kravitz, H. M., Zhao, X., Bromberger, J. T., Gold, E. B., Hall, M. H., Matthews, K. A., & Sowers, M. R. (2008). Sleep disturbance during the menopausal transition in a multi-ethnic community sample of women. *Sleep*, *31*(7), 979-990.

Mulyani, S. (2013). Menopause akhir siklus menstruasi pada wanita di usia pertengahan. *Yogyakarta: Nuha Medika*, *10*.

Pavone, M. E., & Bulun, S. E. (2012). Aromatase inhibitors for the treatment of endometriosis. *Fertility and sterility*, *98*(6), 1370-1379.

Sasnitiari, S. M. N. N. (2018). Pengaruh Senam Aerobik Low Impac terhadap Pengurangan Keluhan Ibu Premenopause di Wilayah Puskesmas Merdeka Bogor Tahun 2016. *Jurnal Bidan*, *4*(1), 234015..

Sulisetyawati, S. D. (2011). Dampak Menopause Terhadap Konsep Diri Wanita yang Mengalami Menopause di Kelurahan Trengguli Kecamatan Jenawi Kabupaten Karanganyar. *Jurnal Kesehatan Kusuma Husada*.

Thurston, R. C., Sutton-Tyrrell, K., Everson-Rose, S. A., Hess, R., Powell, L. H., & Matthews, K. A. (2011). Hot flashes and carotid intima media thickness among midlife women. *Menopause (New York, NY)*, *18*(4), 352.

Vaughan, S., Wallis, M., Polit, D., Steele, M., Shum, D., & Morris, N. (2014). The effects of multimodal exercise on cognitive and physical functioning and brain-derived neurotrophic factor in older women: a randomised controlled trial. *Age and ageing*, *43*(5), 623-629.Vincent, A., Riggs, B. L.,

Atkinson, E. J., Oberg, A. L. & Khosla, S. 2003. Effect of estrogen replacement therapy on parathyroid hormone secretion in elderly postmenopausal women. *Menopause,* 10**,** 165-71.

Whitmer, R. A., Quesenberry, C. P., Zhou, J., & Yaffe, K. (2011). Timing of hormone therapy and dementia: the critical window theory revisited. *Annals of neurology*, *69*(1), 163-169.

WHO, World Health Organization 2014. Social Determinants of Health in Menopause.

Wulandari, R. C. L. (2016). Terapi sulih hormon alami untuk menopause. *INVOLUSI Jurnal Ilmu Kebidanan*, *5*(10).

AB. Appendix

A. INFORMED CONSENT

INFORMED CONSENT

The undersigned:

Name :

Age :

Gender :

Occupation :

Address :

I have received detailed and clear information regarding:

1. The research entitled " Effects of combination aerobic exercise (brisk walking), strength and balance on cognitive function and mental health in menopausal women".

2. The intervention will be administered to the subjects

3. The benefits of participating as a research subject

4. The potential risks involved

5. The research procedure

and have had the opportunity to ask questions about anything related to the research. Therefore, I voluntarily agree/disagree* to become a research subject with full awareness and without coercion. I hereby make this statement truthfully and without any pressure from any party.

Surabaya, ……..

Principal investigator, Respondent,

(Dani Rahmat Ramadhana) ……………………………

Witnes,

……………………………………………

*) Delete as applicable

B. TRAINING PROGRAM

Component of exercise	Exercises	Examples of exercises	Intensity	Time
**Week 1**				
Aerobics	Brisk walking	Marching on the spot (with arm exercise), single knee lift, single side step, single tap, cross step, step forward/backward, kick	5-6/10 RPE	10 minutes
Strength	All major upper, lower body and core muscle groups (using loop band)	Band biceps curl, Band triceps extension, Band chest press, Band shoulder press, Band bridge abs, Band Squat, Band leg press, lateral band walk	2 sets, 6 reps	10 minutes
Balance	Static balance with two arms support	Stand with feet side by side, tandem walking, one leg stand, heel to toe	N/A	10 minutes
Flexibility and cool down	Static stretch	Neck stretch, arm stretch, shoulder stretch, back stretch, hip stretch, leg stretch, butterfly stretch, cat and camel	2 sets, 6 count	10 minutes
			Total time	40 minutes
**Week 2**				
Aerobics	Brisk walking	Marching on the spot (with arm exercise), single/double knee lift, single/double side step, single/double tap, cross step, step forward/backward, kick	5-6/10 RPE	20 minutes
Strength	All major upper, lower body and core muscle groups (using loop band)	Band biceps curl, Band triceps extension, Band chest press, Band shoulder press, Band bridge abs, Band Squat, Band leg press, lateral band walk	2 sets 8 reps	15 minutes
Balance	Static balance without support	Stand with feet side by side, tandem walking, one leg stand, heel to toe	N/A	10 minutes
Flexibility Stretching and cool down	Static stretch	Neck stretch, arm stretch, shoulder stretch, back stretch, hip stretch, leg stretch, butterfly stretch, cat and camel	2 sets, 8 count	15 minutes
			Total time	60 minutes

**Number sequence in CIOMS 2016 Original Protocol*
