# Supplementary material for: Short-term multicomponent exercise training improves executive function in postmenopausal women
Source: PLoS One. 2024 Aug 14;19(8):e0307812. doi: 10.1371/journal.pone.0307812 (PMC11324113; doi:10.1371/journal.pone.0307812)
Supplement: Attachment — Submitted filename: PONE-D-23-28550_R2_RA.pdf [file pone.0307812.s009.pdf]

PLOS ONE

Short-term multicomponent exercise training improves executive function in postmenopausal women

--Manuscript Draft--

**Manuscript Number:**	PONE-D-23-28550R2
**Article Type:**	Clinical Trial
**Full Title:**	Short-term multicomponent exercise training improves executive function in postmenopausal women
**Short Title:**	Effects of combined exercise training on cognitive function in women
**Corresponding Author:**	Raden Argarini, M.D<br>Universitas Airlangga Fakultas Kedokteran<br>Surabaya, East Java INDONESIA
**Keywords:**	Exercise; Women; post-menopause; cognitive; Executive Function
**Abstract:**	Declined cognitive function is comm[illegible]der women, during the menopausal transition and continues [illegible]rch has suggested that combining different exercises stimulates various areas of the brain, potentially leading to greater improvements in cognitive function. However, evidence of the benefits that accrue with multicomponent exercise training, specifically for postmenopausal women is limited. Therefore, this study aimed to investigate the effects of short-term multicomponent exercise training programs on executive function in postmenopausal women. Thirty women (age, 59.8 ± 5.2 years), who were at least 12 months post menopause were allocated into a control (CON) group and an exercise (EX) training group. The EX group underwent a 2-week (five times/week) of multicomponent exercise program comprising aerobic, strength, flexibility, and balance exercises for 40-60 min. Executive function was assessed using the Stroop test and global cognitive function was assessed using the Mini-Mental State Examination (MMSE) at baseline (pre) and after 2 weeks (post) of exercise. Reduced inhibition reaction time of the Stroop test was observed in the EX group (d = -0.78; p = 0.039). Moreover, reduction of total error was observed in all tasks of the Stroop test following exercise intervention (color naming: d = -0.82, p = 0.032; word reading: d = -0.90, p = 0.020; inhibition: d = -1.02, p = 0.009; and switching: d = -0.96, p = 0.012). Additionally, statistical analysis on MMSE score showed a significant improvement (d = 1.31; p = 0.001). In conclusion, our findings suggest that a short-term multicomponent exercise program improves selective tasks of executive function in postmenopausal women along with global cognitive function.<br>Please consider simplifying the introductory lines
**Order of Authors:**	Dani Rahmat Ramadhana
	Rizki Prayuda Putra
	Michelle Abigail Sibarani
	Sulistiawati Sulistiawati
	Dewi Ratna Sari
	Purwo Sri Rejeki
	Lilik Herawati
	Raden Argarini, M.D
**Opposed Reviewers:**	
**Response to Reviewers:**	20 May 2024<br><br>Dr. Emily Chenette<br>Editor in Chief<br>PLoS ONE<br><br><br>Dear Dr. Emily Chenette,

Powered by Editorial Manager® and ProduXion Manager® from Aries Systems Corporation

	Thank you for reviewing our manuscript and the opportunity to revise our manuscript.<br>We have made changes accordingly.<br>We have submitted our second revision documents including a rebuttal letter, track changes, and a clean version of the manuscript.<br>Should you need other information, please do not hesitate to contact us.<br><br>I look forward to hearing from you in this regard.<br><br>Kind Regards,<br>Argarini
**Additional Information:**	
**Question**	**Response**
**Financial Disclosure**<br><br>Enter a financial disclosure statement that describes the sources of funding for the work included in this submission. Review the submission guidelines for detailed requirements. View published research articles from *PLOS ONE* for specific examples.<br><br>This statement is required for submission and **will appear in the published article** if the submission is accepted. Please make sure it is accurate.<br><br>**Unfunded studies**<br>Enter: *The author(s) received no specific funding for this work.*<br><br>**Funded studies**<br>Enter a statement with the following details:<br>• Initials of the authors who received each award<br>• Grant numbers awarded to each author<br>• The full name of each funder<br>• URL of each funder website<br>• Did the sponsors or funders play any role in the study design, data collection and analysis, decision to publish, or preparation of the manuscript?<br>• **NO** - Include this sentence at the end of your statement: *The funders had no role in study design, data collection and analysis, decision to publish, or preparation of the manuscript.*<br>• **YES** - Specify the role(s) played.<br><br>* typeset	This research is funded by the Directorate General of Higher Education, Research, and Technology - Ministry of Education, Culture, Research, and Technology based on Decree Number 0536/E5/PG.02.00/2023 and contract agreement number 114/E5/PG.02.00.PL/2023; 1187/UN3.LPPM/PT.01.03/2023<br>The Ministry of Research, Technology and Higher Education of the Republic of Indonesia did not have any involvement in this study design, data collection or analysis the results, or writing the report.
**Competing Interests**	The authors have declared that no competing interests exist.

Powered by Editorial Manager® and ProduXion Manager® from Aries Systems Corporation

Use the instructions below to enter a competing interest statement for this submission. On behalf of all authors, disclose any competing interests that could be perceived to bias this work—acknowledging all financial support and any other relevant financial or non-financial competing interests.<br><br>This statement is required for submission and **will appear in the published article** if the submission is accepted. Please make sure it is accurate and that any funding sources listed in your Funding Information later in the submission form are also declared in your Financial Disclosure statement.<br><br>View published research articles from *PLOS ONE* for specific examples.<br><br>**NO authors have competing interests**<br><br>Enter: *The authors have declared that no competing interests exist.*<br><br>**Authors with competing interests**<br><br>Enter competing interest details beginning with this statement:<br><br>*I have read the journal's policy and the authors of this manuscript have the following competing interests: [insert competing interests here]*<br><br>* typeset	
**Ethics Statement**<br><br>Enter an ethics statement for this submission. This statement is required if the study involved:<br><br>• Human participants<br>• Human specimens or tissue<br>• Vertebrate animals or cephalopods<br>• Vertebrate embryos or tissues<br>• Field research	This study has been approved by the Ethics Committee of the Faculty of Medicine, Airlangga University with reference number 44/EC/KEPK/FKUA/2023.<br>All participants were provided with written informed consent before their enrollment into this study.

Powered by Editorial Manager® and ProduXion Manager® from Aries Systems Corporation

Write "N/A" if the submission does not require an ethics statement.

General guidance is provided below. Consult the submission guidelines for detailed instructions. **Make sure that all information entered here is included in the Methods section of the manuscript.**

Format for specific study types

Human Subject Research (involving human participants and/or tissue)

- Give the name of the institutional review board or ethics committee that approved the study
- Include the approval number and/or a statement indicating approval of this research
- Indicate the form of consent obtained (written/oral) or the reason that consent was not obtained (e.g. the data were analyzed anonymously)

Animal Research (involving vertebrate animals, embryos or tissues)

- Provide the name of the Institutional Animal Care and Use Committee (IACUC) or other relevant ethics board that reviewed the study protocol, and indicate whether they approved this research or granted a formal waiver of ethical approval
- Include an approval number if one was obtained
- If the study involved *non-human primates*, add *additional details* about animal welfare and steps taken to ameliorate suffering
- If anesthesia, euthanasia, or any kind of animal sacrifice is part of the study, include briefly which substances and/or methods were applied

Field Research

Include the following details if this study involves the collection of plant, animal, or other materials from a natural setting:

- Field permit number
- Name of the institution or relevant body that granted permission

Powered by Editorial Manager® and ProduXion Manager® from Aries Systems Corporation

**Data Availability**<br><br>Authors are required to make all data underlying the findings described fully available, without restriction, and from the time of publication. PLOS allows rare exceptions to address legal and ethical concerns. See the PLOS Data Policy and FAQ for detailed information.<br><br>A Data Availability Statement describing where the data can be found is required at submission. Your answers to this question constitute the Data Availability Statement and **will be published in the article**, if accepted.<br><br>**Important:** Stating 'data available on request from the author' is not sufficient. If your data are only available upon request, select 'No' for the first question and explain your exceptional situation in the text box.<br><br>Do the authors confirm that all data underlying the findings described in their manuscript are fully available without restriction?	Yes - all data are fully available without restriction
**Describe where the data may be found in full sentences. If you are copying our sample text, replace any instances of XXX with the appropriate details.**<br><br>• If the data are **held or will be held in a public repository**, include URLs, accession numbers or DOIs. If this information will only be available after acceptance, indicate this by ticking the box below. For example: *All XXX files are available from the XXX database (accession number(s) XXX, XXX.*).<br>• If the data are all contained **within the manuscript and/or Supporting Information files**, enter the following: *All relevant data are within the manuscript and its Supporting Information files.*<br>• If neither of these applies but you are able to provide **details of access elsewhere**, with or without limitations, please do so. For example:	All relevant data are within the manuscript and its Supporting Information files.

Data cannot be shared publicly because of [XXX]. Data are available from the XXX Institutional Data Access / Ethics Committee (contact via XXX) for researchers who meet the criteria for access to confidential data.<br><br>*The data underlying the results presented in the study are available from (include the name of the third party and contact information or URL).*<br>• This text is appropriate if the data are owned by a third party and authors do not have permission to share the data.<br><br>* typeset	
Additional data availability information:	

Raden Argarini
Faculty of Medicine - Airlangga University

20 May 2024

Dr. Emily Chenette
Editor in Chief
PLoS ONE

Dear Dr. Emily Chenette**,**

Thank you for reviewing our manuscript and the opportunity to revise our manuscript. We have made changes accordingly.

We have submitted our second revision documents including a rebuttal letter, track changes, and a clean version of the manuscript.

Should you need other information, please do not hesitate to contact us.

I look forward to hearing from you in this regard.

Kind Regards,

Raden Argarini, M.D.,Ph.D

Manuscript Click here to access/download;Manuscript;Manuscript.docx

Clinical trial

Short-term multicomponent exercise training improves executive function in postmenopausal women

Dani Rahmat Ramadhana[1], Rizki Prayuda Putra[1], Michelle Abigail Sibarani[1], Sulistiawati[3], Dewi Ratna Sari[4], Purwo Sri Rejeki [1,2,5], Lilik Herawati [1,2,5], Raden Argarini[1,2,5*]

[1] Master Degree on Sport, Exercise, and Health Sciences, Faculty of Medicine, Airlangga University, Surabaya, Indonesia

[2] Undergraduate Medical Programme, Faculty of Medicine, Airlangga University, Surabaya, Indonesia

[3] Department of Public Health, Faculty of Medicine, Airlangga University, Surabaya, Indonesia

[4] Department of Anatomy, Histology and Pharmacology, Faculty of Medicine, Airlangga University, Surabaya, Indonesia

[5] Department of Medical Physiology and Biochemistry, Faculty of Medicine, Airlangga University, Surabaya, Indonesia

***Corresponding Author:**

Email: raden-a@fk.unair.ac.id (RA)

Trial Registration: ISRCTN13086152

Abstract

Declined cognitive function is commonly complained among older women, during the menopausal transition and continues afterward. Recent research has suggested that combining different exercises stimulates various areas of the brain, potentially leading to greater improvements in cognitive function. However, evidence of the benefits that accrue with multicomponent exercise training, specifically for postmenopausal women is limited. Therefore, this study aimed to investigate the effects of short-term multicomponent exercise training programs on executive function in postmenopausal women. Thirty women (age, 59.8 ± 5.2 years), who were at least 12 months post menopause were allocated into a control (CON) group and an exercise (EX) training group. The EX group underwent a 2-week (five times/week) of multicomponent exercise program comprising aerobic, strength, flexibility, and balance exercises for 40-60 min. Executive function was assessed using the Stroop test and global cognitive function was assessed using the Mini-Mental State Examination (MMSE) at baseline (pre) and after 2 weeks (post) of exercise. Reduced inhibition reaction time of the Stroop test was observed in the EX group ($d = -0.78$; $p = 0.039$). Moreover, reduction of total error was observed in all tasks of the Stroop test following exercise intervention (color naming: $d = -0.82$, $p = 0.032$; word reading: $d = -0.90$, $p = 0.019$; inhibition: $d = -1.02$, $p = 0.009$; and switching: $d = -0.96$, $p = 0.012$). Additionally, statistical analysis on MMSE score showed a significant improvement ($d = 1.31$; $p = 0.001$). In conclusion, our findings suggest that a short-term multicomponent exercise program improves selective tasks of executive function in postmenopausal women along with global cognitive function.

Introduction

In older women, declined cognitive function and mood changes are frequent complaints before, during, and after the menopausal transition. A recent review has identified that cognitive impairment such as lack of concentration, memory issues, planning difficulties, and brain fog, is commonly seen in 62%–67% of women during the menopausal transition [1]. This impairment continues to decline afterwards [2, 3]. Although the evidence of long-term changes in cognitive function during perimenopause and the progression remain unclear, postmenopausal women are more susceptible to a decline in cognitive function and neurodegerative diseases. For example, women have a higher risk of Alzheimer's disease than men [4]. Furthermore, certain cognitive tests including the verbal memory test are gender sensitive [2].

The impact of menopause on the central nervous systems is mostly caused by rapid decline in the production of ovarian hormones, particularly estrogen. Estradiol, the most biologically active type of estrogen, has neuroprotective effects and elicit it effects through genomic and non-genomic signalling pathway to increase brain derived neurotrophic factor (BDNF) [5]. BNDF is responsible for the promotion of neuronal growth, maturation and maintenance as well as synaptic plasticity and memory consolidation [5]. Moreover, estrogen plays a significant role to protect against oxidative stress-induced cell death in hippocampal neurons and interact with the neurotransmitter system, thereby preventing neurodegeneration [6, 7]. Furthermore, it interacts with insulin-like growth factor-1 (IGF-1), which stimulates neuronal cell survival and proliferation [7].

Although estrogen seems to contribute to the cognitive decline during menopausal transition and afterward, the impact of estrogen replacement therapy to cognitive function in postmenopausal women remains inconclusive [8]. It is believed that the complex signalling

pathways and molecular mechanisms of estrogen provide neuroprotective effects. The reduction in BDNF that accompanies the loss of estrogen may play a significant role in cognitive decline in postmenopausal women [5]. Therefore, strategies aimed to increase BDNF levels in postmenopausal woman may be beneficial for maintaining cognitive function in postmenopausal women. Exercise has been proposed as one of the modalities for preventing a rapid decline in cognitive function in older adults. Several systematic reviews with meta-analysis have shown that exercise training in older adults, regardless of their cognitive status, has positive effects on cognitive function [9-12] in at least one cognitive domain. The mechanisms of exercise-induced improvement in cognitive function involve several pathways, including increased brain blood flow and growth of blood vessels, alteration of inflammatory cytokines (reduction in TNF-α and IL-6 concentrations), and increased levels of neurotrophic factors (BDNF) [13], independent of the estrogen-linked BDNF pathway [5]. Advanced research in humans, utilizing functional magnetic resonance imaging [14] reported that alterations in cognitive performance are mediated by changes in neural activation in different areas of the brain. Different types of physical activities are associated with the activation of distinct areas of the brain and cognitive functions [14].

The recent meta-analysis had already established the efficacy of exercise training for enhancing cognitive vitality in older adults; however, some moderator variables may interfere with the positive effect of exercise training on cognition [11, 12, 15]. Some studies [16, 17] and meta-analysis [11, 15] demonstrated that women gain more cognitive benefits from exercise training than men. Additionally, the benefits of exercise on cognition vary depending on the doses of exercise intervention (type, intensity, and duration), the characteristics of the participants at the beginning of the study (gender, general health, and fitness level), and the tasks used for measuring the aspects of cognition [11, 15]. Executive function is a cognitive domain that is mostly affected

by aging. However, this function less declined overtime in women compared to men [18]. Therefore, compared with men, the neural circuitry underlying this domain may remain more intact and may be more sensitive to targeted exercise intervention in women.

A recent study suggested that combining different types of exercises including cardiovascular, resistance, balance, and flexibility exercises can achieve greater gains in cognitive function [13]. Moreover, the World Health Organization strongly suggested older adults to engage in multicomponent exercises to enhance wide range of physical function, prevent falls, manage weight, and increase bone health and prevent osteoporosis [19]. However, evidence of the benefits that accrue with this type of exercise specifically to postmenopausal women is lacking. Furthermore, evidence of the short-term benefits of exercise program to cognitive function is limited. Therefore, this study aimed to investigate the short-term effects of a multicomponent exercise training program comprising aerobic (brisk walking), resistance, flexibility, and balance exercises on executive function together with global cognitive function in postmenopausal women. According to the latest fitness trend, aerobic and resistance training in the form of functional fitness as included in the exercise intervention in this study remain popular worldwide [20]. Several cognitive tasks have been used in studies evaluating the effects of exercise training on cognition, and the Stroop test and Mini-Mental State Examination (MMSE) were particularly selected as neuropsychological assessments to measure executive function and global cognitive function, respectively [21, 22]. The Stroop test is sensitive to exercise training and consists of tasks that allow for the assessment of basic information processing and higher-order aspects of cognition associated with frontal lobes activity [21]. The MMSE is a global clinical, psychological, neuropsychological indicator usually used for screening and evaluating the cognitive status of patients. It can comprehensively, accurately, and quickly reflect the intellectual quality and

cognitive impairment of patients. We hypothesized that we could observe an improvement in executive function in addition to global cognitive function following a short-term multicomponent exercise intervention.

Materials and Methods

Ethics statement and trial registration

This study has been approved by the Ethics Committee of the Faculty of Medicine, Airlangga University, Indonesia (reference number 44/EC/KEPK/FKUA/2023). After a thorough explanation of the testing protocol, the possible risks involved, and the right to terminate participation at anytime, all participants provided informed consent before their enrolment into this study. Participant recruitment started on February 27, 2023 and ended on April 13, 2023. This study was registered in International Standard Randomised Controlled Trial Number (registration number: ISRCTN3086152). This study was prospectively registered as the type intervention (exercise) allows us to register the trial as ongoing/completed study after the first participant enrolment. The authors confirm that all ongoing and related trials for this intervention are registered.

Participants' characteristic

The participants in this study were recruited from women's organizations that participated in the Family Welfare Empowerment Program in Surabaya, East Java, Indonesia. Those who met the initial eligibility requirements (women aged 50-80 years old who are at least 12 months postmenopausal and had a minimum educational level of junior high school) were invited to undergo the next stage of screening. Subsequently, the participants were screened to ensure that

they did not have a history of stroke or any neurological problems, recent surgery, severe hearing or visual impairment, recent cardiovascular diseases (e.g., ischemic heart diseases and heart failure), mental health problems, dementia, or moderate-to-severe cognitive impairment [defined as a score of <21 on the MMSE) [23]. Moreover, to ensure that they were not experiencing moderate-severe stress, psychological stress was assessed using the Depression Anxiety Stress Scale-21 (DASS-21).

Study design

The Consolidated Standards of Reporting Trials (CONSORT) diagram outlining participants' flow form the first contact to the study completion is depicted in Fig 1. Participants who met the inclusion criteria were allocated to the exercise training group (EX, n = 15). Age-matched participants were recruited from the similar women organization and allocated to the control group (CON, n = 15). Participants in the EX group underwent a 2-week exercise program that included a combination of aerobic, strength, balance, and flexibility exercises. Conversely, those in the CON group were asked to continue with their usual activities. The short-duration exercise program was developed to be feasible in the community setting [24]. Additionally, cognitive improvement (verbal fluency test) were observed after short program (14 days) of home-based exercise in combination with diet and relaxation in middle-aged participants (age, 35-69 years) [25]. The outcomes were assessed at baseline and 3 days following the final exercise session. At the initial meeting, all participants were asked to complete a form to collect their baseline information, including educational level, marital status, medical history, and medication. All the measurements were performed in a controlled-temperature room (25°C) at the same time

(06:00 – 08:00 AM). The data analyzer was blinded to the participant allocation. Blinding of participants was not possible in this study.

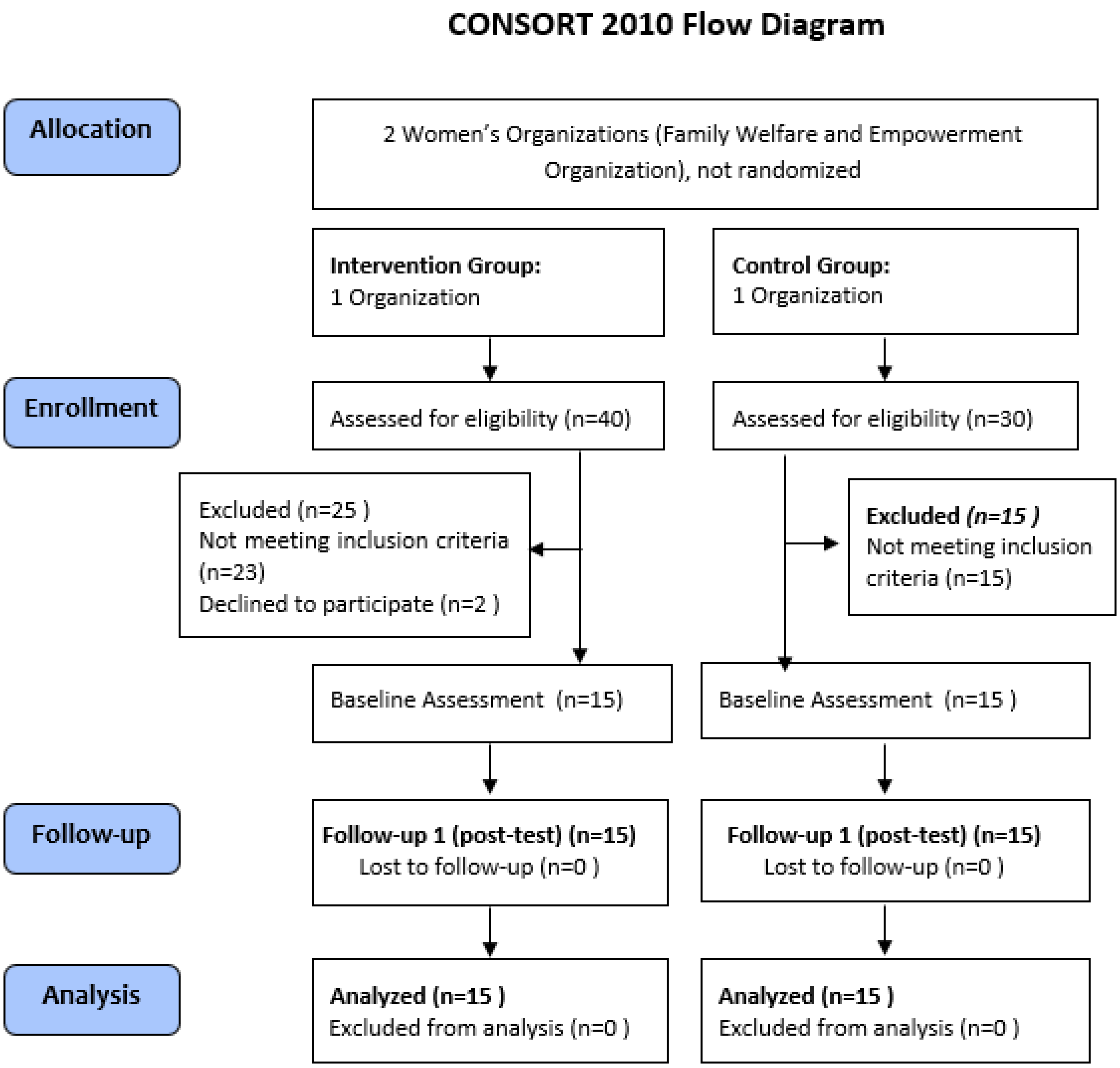

Fig 1. Diagram of participants flow (modified for non-randomized study)

Following enrolment, a pretest was conducted 3 days before the first exercise session, and a posttest was conducted 3 days after the last exercise session. Before outcome measurements, all

participants were asked to fast for 10 h (last meal at 8 PM on the previous day) and refrain from smoking, consuming alcohol or carbonated drinks, and engaging in vigorous exercise. Upon participants' arrival, their blood glucose levels were measured, and they were allowed a light breakfast (2-3 pieces of biscuits, provided by the research team). Resting heart rate and blood pressure were measured two times using an automatic sphygmomanometer (OMRON Model HEM-7156; Omron Healthcare Co., Ltd, Japan). Global cognitive function and the executive functions were assessed using the MMSE and the Stroop test, respectively.

Exercise training

Participants in the EX group attended exercise training five times/week for 2 weeks, every day on weekdays, in a community-based hall. Each exercise session was performed by groups. Each exercise session comprised a combination of aerobic (brisk walking), strength (using loop bands), balance and flexibility exercises. In the first week, each session lasted for 40 min and progressively increased to 60 min. The overall intensity of exercise was moderate (RPE 5-6 of scale 10). The detail of exercise intervention is provided in the S1 Table. The exercise was led by one instructor and two others who assisted and monitored the movements performed by the

articipants during the exercise. Participants' attendance was recorded in the logbook, and the adherence rate was calculated on the basis of the number of completed training sessions.

Outcomes measurement

Executive function (Stroop Test)

Executive function was assessed using a modified Stroop test [26] that consists of four different components and is adapted in Bahasa [27, 28]. The intraclass correlation for reaction time and total error was 0.91 and 0.62 respectively [28]. Each component was printed on an A4 sheet of paper and consisted of practice (10 stimuli, five items per line) and test (50 stimuli, 10 items per line). The first condition was word reading, wherein the participants should read the words that were printed using black ink (e.g., red, green, blue, yellow). In the second condition (color naming), the participants should name the color of the rectangles. In the third condition (inhibition), the word was printed in different colors with their meaning. For example, the word "green" was printed using red ink. The participants should name the ink color of the printed words. Lastly, compared to the aforementioned tests, the fourth condition (inhibition/switching) was the most complex test. This test is similar to the third condition (inhibition); however, of 50 items printed color words; 20 were surrounded by a rectangle. In this condition, participants should read the printed words instead of naming the color. Before each test, to ensure their understanding of the task, the participants were provided instructions and an opportunity to practice. For all conditions, participants should respond as fast as possible and point to which part they are currently on. They were allowed a maximum of four corrections for each test. All tasks were discontinued if the participants had (1) made four uncorrected errors or (2) reached the time limit for each task.

The time limit for tasks 1 and 2 is 90 seconds, while the time limit for tasks 3 and 4 is 180 seconds. The completion testing time (reaction time, RT) was recorded in seconds. The total number of errors was determined by accumulating the corrected and uncorrected errors [29].

Global cognitive health (MMSE)

To assess global cognitive function, including memory, attention, orientation, language, and visuo-spatial ability, we used the Indonesian version of MMSE. The MMSE score ranges from 0 to 30 points; in the Indonesia version, an MMSE score at or below 24 was classified as cognitive dysfunction or dementia. This cutoff point showed a sensitivity and specificity of 88% and 96%, respectively [30]. The Indonesian version MMSE had already undergone validation and reliability test, with Cronbach alpha of 0.87 [31].

Statistical analysis

The sample size calculation was calculated using G*power software version 3.1.9.7 based on the published data of Vaughan *et al*. (2014) [13] which reported the beneficial effects of a 16-week multimodal exercise program (including aerobic, strength, balance, flexibility, coordination, and agility exercises) on cognitive performance in older women. The study reported an effect size of 0.77 for the Stroop test (color naming). The minimum number of subjects required to establish significance is 14 per group would provide an 80% power (α=0.05) for F-test (ANOVA) repeated measures, within-between interaction.

All statistical analysis was computerized using software Jamovi 2.3.21.0 [32]. We performed the analysis of distribution using a visual inspection of Q-Q plot, skewness and kurtosis,

and Shapiro Wilk test. The independent t-test was used to compare basic characteristics data of participants, whereas the outcome variables were assessed using linear mixed models. These models enabled the analysis of differences in baseline cognitive performance between the two groups, differences between baseline and post-exercise cognitive performance within each group, and the assessment of [illegible] e (pre vs post) and group (contr[illegible] o address the individual variatio[illegible] ompound symmetry, unstructure[illegible] was based on the Akaike's Infor[illegible] e simpler model was chosen. T[illegible] ovariance structure used in the linear mixed model. Based on that model, we investigated the interaction between time and group and the main effects separately (time: pre vs post and group: CON vs EX). This mixed modelling can deal efficiently with missing data, unbalanced time points [33] and robust with the violations in distributional assumptions [34]. This means that the missing data could be included in the analyses, without imputation. This method also compensates for selective dropout, on the condition that d[illegible] there were no missing data in thi[illegible] a bootstrap procedure was performed with 10,000 resamples to obtain reliable standard errors and p-values. The analyses were carried out by the per protocol approach. Only those who attended a minimum of eight exercise sessions (80%) were included in the post-test. Estimated means, standard deviations and 95% confidence intervals, and the estimation of the interaction between time and treatment in the model are presented. Bonferoni test was used for post-hoc test, with significance level of 95%. Effect sizes of the improvements observed according to Cohen's-d

Linear mixed models were used to analyse differences in baseline and post-exercise cognitive performance. Time (pre vs. post) and group (control vs. exercise) were fixed factors, and participants were random factors to account for individual variations. We tested three covariance structures: compound symmetry, unstructured, and autoregressive, selecting compound symmetry based on the corrected Akaike Information Criterion (AIC).

This is more clear and direct.

The authors might want to expand for only AIC.

For non-normal residuals (residuals?), a bootstrap procedure with 10,000 resamples was used to obtain reliable standard errors and p-values

Effects were calculated using [...] where [small] etc, Hedges', in the updated

statistic were computed as the absolute change of the outcome measure to its pooled sd. in the group assessed before and after training. Effect size were considered as trivial (<0.2), small (0.2 to 0.49), moderate (0.50 to 0.79) and large (≥0.80) [35]. Graphs were made using PRISM 9.5.1 (Graph Pad Software, La Jolla, CA, USA).

Results

Participant characteristics

Of the 15 participants enrolled in the EX group, the adherence to exercise program was 98.7%. The baseline characteristics of the participants are presented in Table 1. No significant differences in age, body mass index, resting heart rate, and blood pressure were observed (all $p > 0.05$) (Table 1). Two participants in the EX group were diagnosed with type-2 diabetes mellitus and were treated with oral antidiabetic medication. Three participants (two and one participants in the CON group and EX group, respectively) were diagnosed with hypertension and were treated with an oral antihypertensive medication. Other information on participants characteristics is shown in Table 1.

Table 1. Participant's baseline characteristic [illegible]

	**Cont**[illegible] **(n=1**[illegible]	[illegible]	**P-Value** [illegible]pendent T-test)
Age, year	58.27 ± [illegible]	[illegible]	0.116
Height, cm	154.00 ± [illegible]	[illegible]	0.557
Body Weight, kg	59.20 ± [illegible]	[illegible]	0.919
BMI, kg/m^2	24.90 ± [illegible]	[illegible]	0.629
Resting heart rate, beats/min	79.53 ± 8.46	81.90 ± 9.29	0.472
Blood pressure			
Systolic blood pressure, mmHg	127.40 ± 18.63	132.30 ± 14.40	0.427
Diastolic blood pressure, mmHg	78.87 ± 10.15	83.63 ± 7.92	0.163
Mean arterial pressure, mmHg	95.01 ± 12.22	99.89 ± 9.47	0.232

Check these p-; I've calculated (in sequence):

Age = 0.286
Height = 0.567 (correct)
Weight = correct
BMI = 0.626 (correct)
Resting rate = 0.471 (correct)
Systolic = 0.427 (correct)
Diastolic = 0.163 (correct)
Arterial pressure = 0.232 (correct)
FPG = 0.512
DASS-21 D = 0.556
DASS-21 Anx = 0;334
Stress = 0.644

FPG mg/dL	117.40 ± 32.90	128.70 ± 57[illegible]	[illegible]
Education			
Junior high school	0 (0%)	3(20 %)	
Senior high School	7 (46.6%)	10 (66.6 %)	
Diploma	1 (6.6%)	2 (13.3%)	
Bachelor	7 (46.6%)	0 (0%)	
Medical History			
Diabetes mellitus	0 (0%)	2 (13.3%)	
Hypertension	2 (13.3%)	1 (6.6%)	
History of cardiovascular diseases	0 (0%)	0 (0%)	
History of cerebrovascular diseases	0 (0%)	0 (0%)	
Current or ex-smoker	0 (0%)	0 (0%)	
Medication			
Oral antidiabetic	0 (0%)	2 (13.3%)	
Antihypertensive	2 (13.3%)	1 (6.6%)	
Mental Health (DASS-21 Score)			
Depression	1.33 ± 1.39	1.87 ± 3.22	0.564
Anxiety	2.46 ± 1.95	3.40 ± 3.15	0.341
Stress	3.66 ± 2.89	3.13 ± 3.31	0.646

BMI, body mass index; DASS-21, Depression Anxiety Stress Scale-21; FPG, fasting Plasma Glucose; all data are presented as mean ± standard deviation (SDs). *significantly different from control group at $p < 0.05$.

Comment: I think you can remove the 0, and just place a dash or something

Comment: p = 0.225 / p = 0.462 / p = 1/ / p = 0.006 / Overall = 0.012 (the averaged is 0.424 and it's more reliable)

Comment: p = 0.484

Comment: Averaged p = 0.899

Comment: Constancy in capitalisation, e.g. Like this for the note OR Like This The Note

Effects of exercise on cognitive test

Stroop test performance

The results of the Stroop test on completion time performance (reaction time) and total errors before and after exercise are presented in Table 2 and Figs 2 and 3. In the word reading performance, we did not found a significant interaction ($p = 0.081$) or main effects time (p time = 0.079; p group = ([illegible]0). We detected [illegible] [illegible]aring differences from baseline of the EX group versus CON group (d = -0.66 (95% CI: -1.41 - 0.11), $p = 0.081$).The analysis of the total errors in this task revealed a significant interaction ($p = 0.019$) and a main effect of time ($p = 0.027$). Moreover, we found significant large reducti[illegible] in this task when comparing to the baseline in the EX group versus CON group (d = -0.90 (95% CI: -1.68 [illegible].10), $p = 0.019$).

Comment: There is no p time or p group; just use p and the test, please

Comment: Effect and test with CI

Comment: Effect and test with CIs

Comment: Use absolute values, i.e. positive. CIs is 1.41 insead of 1.43. Check the CIs, if the values crossed zero, then it's not significant at al..

Comment: Quite unusual such effect, and use absoute value please, i.e. positive. Check the CIs, if the values crossed zero, then it's not significant at al..

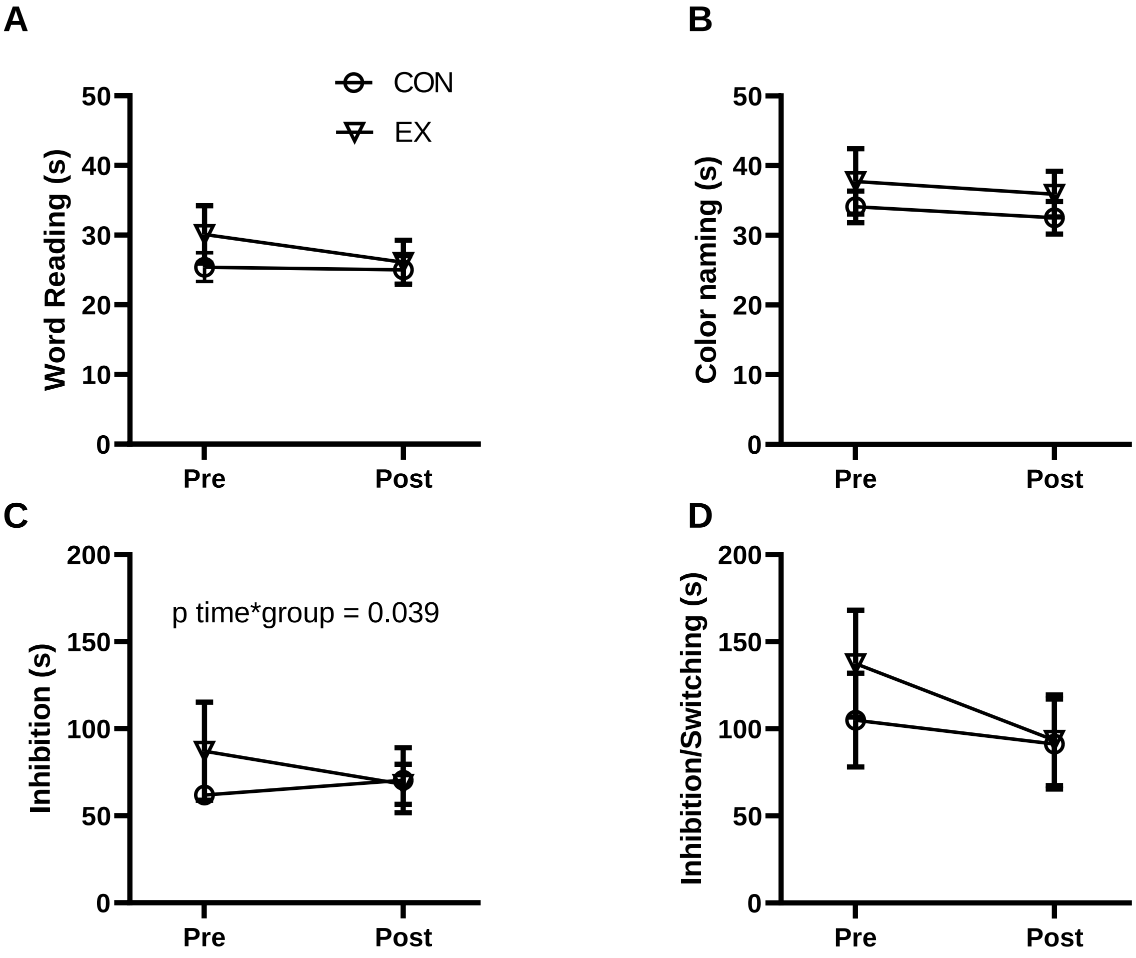

Fig 2. Effects of exercise intervention on reaction time of the Stroop test performance in postmenopausal women. Reaction time to complete the word reading task (top, left panel – A), color naming task (top, right panel –B), inhibition task (bottom, left panel – C), and inhibition/switching task (bottom, right panel – D) before (pre) and after (post) 2-weeks. Data is presented as means ± 95% confidence intervals (CI). Effects are derived from linear mixed models, adjusted for age, years of education, and DASS-21 score.

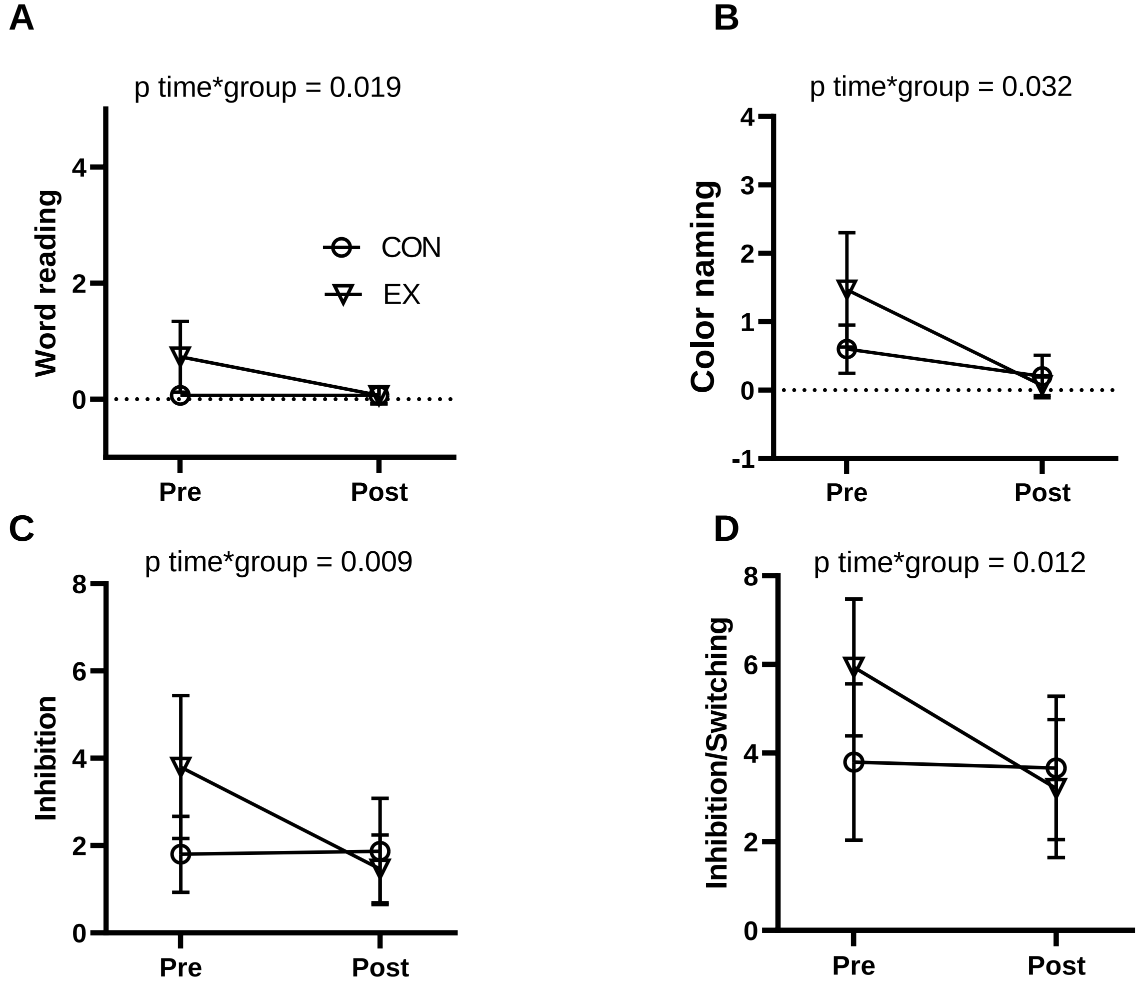

Fig 3. Effects of exercise intervention on total errors of the Stroop test in postmenopausal women. Total errors during the completion of the word reading task (top, left panel – A), color naming task (top, right panel –B), inhibition task (bottom, left panel – C), and inhibition/switching task (bottom, right panel – D) before (pre) and after (post) 2-weeks. Data is presented as means ± 95% confidence intervals (CI). Effects are derived from linear mixed models, adjusted for age, years of education, and DASS-21 score.

Table 2. Stroop test and MMSE assessment at baseline (pre) and we [illegible]

	Control (n=15)		[illegible]		[illegible]		[illegible] difference from [illegible]line (95% CI)		
	pre mean (95%CI)	post mean (95%CI)	pre mean (95%CI)	post mean (95%CI)	[illegible] mean (95% CI)	[illegible]	[illegible]	Exercise Mean (SE)	ES d
**Stroop test - Reaction time, second**									
Word Reading[a]	25.41 (23.35, 27.46)	25.03 (23.09, 26.96)	30.12 (25.99, 34.24)	26.10 (22.95, 29.25)	-3.57 (- 7.44, 0.24)	0.081	-0.08 (1.42)	-3.65 (1.43)	-0.66
Color naming[a]	34.09 (31.82, 36.36)	32.51 (30.18, 34.83)	37.73 (33.05, 42.42)	35.91 (32.65, 39.17)	-0.22 (-4.46, 3.86)	0.916	-1.50 (1.51)	-1.72(1.53)	-0.04
Inhibition[a]	61.87 (57.05, 66.69)	70.44 (51.82, 89.07)	87.09 (58.93, 115.25)	68.06 (56.61, 79.51)	-27.08 (-51.55, 2.63)*	0.039	10.77 (8.96)	-16.31 (9.03)	-0.78
Inhibition/ Switching[a]	104.99 (78.07, 131.90)	91.19 (65.33, 117.04)	137.41 (106.81, 168.02)	93.42 (67.39, 119.45)	-29.69 (-69.86, 11.75)	0.168	-11.72 (15.03)	-41.41 (15.14)	-0.52
**Stroop test - Total Error**									
Word Reading[a]	0.07 (-0.08, 0.21)	0.07 (-0.08, 0.21)	0.73 (0.124 - 1.34)	0.07 (-0.08 - 0.21) †	-0.66 (-1.18 – 0.014)*	0.019	0.01 (0.19)	-0.65 (0.19)*	-0.90
Color naming[a]	0.60 (0.25, 0.95)	0.20 (-0.11, 0.51)	1.47 (0.63 - 2.30)	0.07 (-0.08 - 0.21) †	-0.99 (-1.87 – (-0.15)*	0.032	-0.37 (0.31)	-1.37 (0.31)*	-0.82
Inhibition	1.80 (0.93, 2.67)	1.87 (0.65, 3.08)	3.80 (2.16 - 5.44)	1.47 (0.69 - 2.25) †	-2.38 (-4.07 – (-0.68)*	0.009	0.17 (0.60)	-2.21 (0.61)*	-1.02
Switching	3.80 (2.03, 5.57)	5.93 (2.05, 5.28)	3.67 (4.39 - 7.48)	3.20 (1.64 - 4.76) †	-2.55 (-4.46 – (-0.64)*	0.012	0.09(0.69)	-2.46 (0.69)*	-0.96
**MMSE score (0-30)**	25.33 (24.03, 26.63)	26.47 (25.63 – 27.30)	24.20 (22.82 – 25.58)	28.47 (27.63 – 29.30) †	3.11 (1.37 – 4.85)*	0.001	1.04 (0.62)	4.15 (0.63) *	1.31

Values are from linear mixed effects models adjusted for age, years of education, and DASS-21 score.
*Significant time*treatment effect ($p < 0.05$)
†Significant time effect ($p < 0.05$) compared with baseline (pre).
[a]Bootstrap for non-normal distribution of the residuals
ES: Effect size

Should be recalculated

Mean (SD) or Mean (SD).
Then other column for CI. Then the differences, if necessary. But the differences can be in running text. Also need recalculation.

For the mixed analyses, n = 60?

Seems values are a little bit different. For instance, the M (SE) for the first variable should be - 0.38 (1.43); exercise = -4.02 (2.64).
This was for almost values.

Is this CI correct?
If yes, the significance probable at chance such dispersion

Constancy, or CIs or SE ; or maybe M (SE) and other column with CIs

Values are in Table, no need to repeat here.

Values seemed similar to standard CIs.
Boostrap CIs are a little bit different because of resampling (place BCa)

How similar means have such different CIs?

Mean outside the CI. Recalculate

Recalculate.
Mean outside CI

Please use Hedges, due to the sample

Regarding color naming reaction time, a significant interaction or main effect was not observed (all $p > 0.05$). We found a non-significant trivial reduction when comparing differences from baseline of the EX group versus CON group ($d = -0.04$ (95% CI: -0.76 - 0.67), $p = 0.916$, Table 2 and Fig 2). In the total errors, we found a significant interaction ($p = 0.032$) and a time effect ($p < 0.001$) without group effect ($p = 0. 315$). Additionally, we detected a large significant reduction when comparing differences from baseline of the EX group versus CON group ($d = -0.82$ (95% CI: -1.59 - 0.04), $p = 0.032$).

Effects and CI

In the inhibition task, we found that both reaction time and total errors have a significant interaction ($p = 0.039$, $p = 0.009$). Additionally, we observed significant large reduction when comparing differences from baseline of the EX group versus CON group of the reaction time ($d = -0.78$ (95% CI: -1.54 - 0.00), $p = 0.039$) and total errors ($d = -1.02$ (95% CI: -1.82, - 0.20), $p = 0.009$).

In the inhibition/switching task, the interaction for the reaction time ($p = 0.168$) was not significant although the main effect of time showed significant results ($p = 0.020$). We observed non-significant moderate reduction when comparing differences from baseline of the EX group versus CON group of the reaction time ($d = -0.52$ (95% CI: -1.25 - 0.23), $p = 0.168$). Additionally, the statistical analysis of the total errors of the IN/SW task showed a significant interaction ($p = 0.012$) and a main effect of time ($p = 0.024$). In line with this, we detected significant large reduction when comparing differences from baseline of the EX group [illegible] f the reaction time ($d = -0.96$ (95% CI: -1.74, - 0.15), $p = 0.012$).

A moderate without significance?

The effect of exercise on global cognitive function (Mini Mental State Examinations - MMSE)

Please check covariance and autocorrelation. Seems a little bit of residuals

The analysis of MMSE showed a significant interaction time*group ($p = 0.001$) and a main effect of time ($p < 0.001$) as presented in (Fig 4). Furthermore, we observed significant large improvement of MMSE score in when comparing differences from baseline of the EX group versus CON group ($d = 1.31$(95% CI: 0.43 – 2.15), $p = 0.001$).

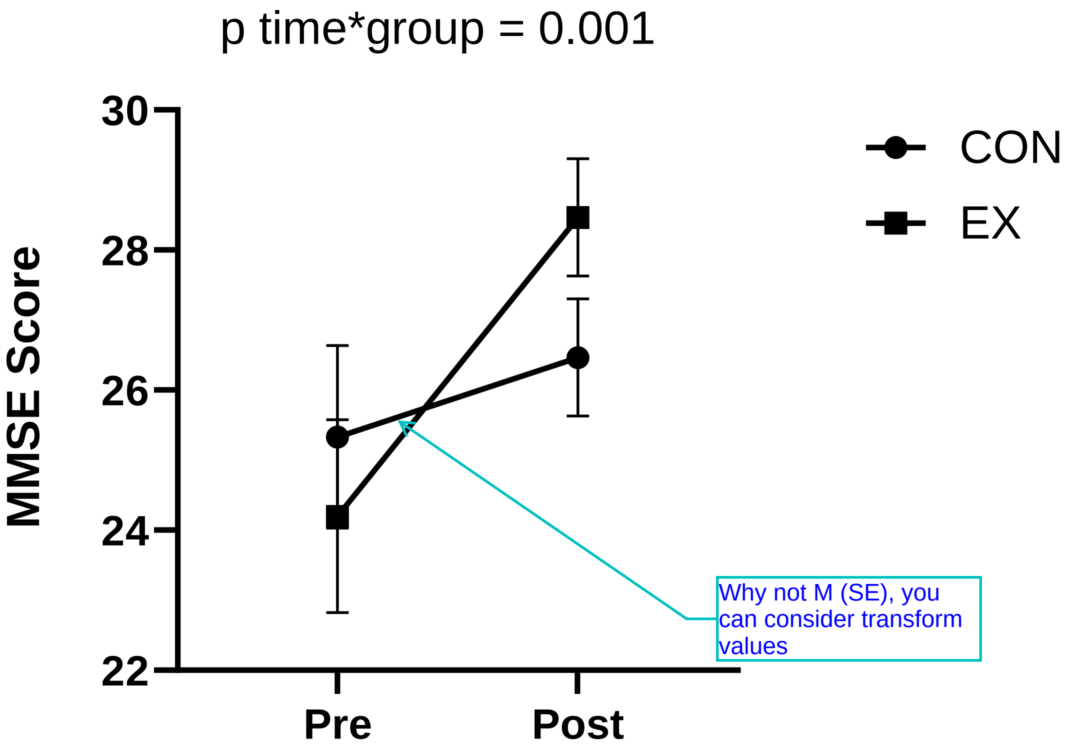

Fig 4. Effects of exercise intervention on MMSE in postmenopausal women. The graph shows MMSE scores before (pre) and after (post) 2-weeks of exercise training in the exercise group and the control group. Data is presented as means ± 95% confidence intervals (CI). Effects are derived from linear mixed models, adjusted for age, years of education and DASS-21 score.

The first para. should be the overall interpretation. You need to branch this into two different, overall interpretation and values and then the link with the other and literature

Discussion

The multicomponent of exercise training programs aimed to improve physical fitness and develop motor learning skills, including balance and flexibility. The main findings of this study include a short-term of multicomponent exercise training [illegible] inhibition task performance of the Stroop test. We noted a positive trend towards improvement of the reaction time of other components of the Stroop task (color naming, word reading, and switching) although did not reach statistical significance. Moreover, we observed the improvement in accuracy to perform the Stroop test in all tasks as shown in the reduction of total errors. Consistent with our results, Vaughan et al. (2014) investigated the effects of a 16-week multicomponent exercise that includes aerobic-strength-motor fitness (balance, coordination, flexibility, and agility) in older women. The results demonstrated moderate-to-large effect size of exercise to improve the reaction time of all tasks of the California Older Stroop Test, including color naming, word reading and inhibition task [13]. In contrast, another study demonstrated no significant effect in all components of the Stroop tasks following a 6-month of multicomponent exercise program in older women [36]. However, this study used a different analysis of the Stroop test by the total number of correct words in 45 s instead of the reaction time and total error [36]. Most studies that investigated the effect of exercise employed aerobic exercises. Predovan et al (2012) evaluated executive functions after a 12-week of aerobic training in older adults (>70% women participants) and noted differences only in the switching condition. Similarly, significant improvement in total error was observed only in the switching component [29]. Another study of aquarobics program in older adults (>70% women participants) showed improvement in the Stroop test performance after 21 weeks but not in shorter-term intervention (10 weeks) [37]. Although generally longer period of exercise intervention provide more benefit in cognitive function, Colcombe and Kramer (2003) in their meta-analysis

Check grammar and the refs.

showed that shorter duration (4-12 weeks) lead to better cognitive improvement than medium duration (12-24 weeks). Additionally, this meta-analysis showed that combined aerobic and resistance exercise demonstrated larger effect compared to aerobic alone [11].

A possible explanation of the discrepancy of our results with those of other studies is the differences in the modality and doses of exercises. The effect of physical exercise on cognitive function has been proven to be task-specific and motor activity specific [38]. Stern (2009) hypothesized that a higher cognitive reserve resulting from physical exercise is because of a neural compensation mechanism that permits complex activities [39]. Complex tasks such as inhibition and inhibition/switching in the Stroop test require multiple executive processes. Chodzko-Zajko et al. (1994) suggested that tasks requiring more control and effortful processing including the Stroop test, should be more sensitive to fitness differences among older adults than tasks that can be processed automatically [40].

In this study, we estimated a moderate effect of exercise on the speed improvement of the inhibition task of the Stroop test and a larger effect on the accuracy of all tasks in the postmenopausal women population. Our positive findings on executive function suggest that exercise has a beneficial effect on controlling functions critical for processes involved in cognitive learning. These functions include information processing speed, attention control, resistance to interference, dual-tasking, and cognitive flexibility [13]. Recent systematic review research has suggested a significant cognitive decline in executive processes during progressive neuropsychological follow-ups, leading to a potential manifestation of mild cognitive impairment (MCI) or dementia [41]. From a clinical perspective, a 15-year prospective study on individuals with Alzheimer's disease revealed that lower cognitive performance at the executive level began to show more pronounced signs 2–3 years before receiving a clinical diagnosis [42]. Thus,

monitoring the performance of executive function tasks and developing interventions to maintain or improve these cognitive functions is necessary to address the epidemic of dementia and other cognitive disorders. However, the generalization of our findings should be approached with caution as our study focused on post-menopausal older women, a group that may be psychologically vulnerable [4] and may potentially experience more benefit on executive processes from exercise than men [15]. In addition, the executive function as the primary outcome of this study is one of cognitive domains that is commonly benefited positively from exercise interventions. Similar intervention might have different effect on other cognitive domains such as verbal fluency.

The second aim of our study was to investigate the global cognitive function using the MMSE. Regarding the MMSE score, our results were consistent with those of Sanchez-Gonzalez et al. (2021). However, this study showed little effect on the MMSE score improvement. This may be because the participants in this group have already high MMSE scores at baseline (mean score > 27) [36]. This test is believed to have a ceiling effect because the highest score is 30. Moreover, our findings are consistent with the results of a meta-analysis study that showed most exercise intervention improve global cognitive function in older individuals [9].

Our study did not investigate the mechanism of cognitive function improvement as a result of exercise intervention. However, several studies have shown that physical exercise improves brain functioning through both structural and functional changes. Studies have reported that exercise training can lead to an increase in gray and white matter volume in the prefrontal cortex and hippocampal regions [43]. The prefrontal area is important to process the executive function and this cognitive task has the most pronounced effect because of exercise training [11]. Moreover, it has been observed to increase the levels of neurotrophic factors, such as BDNF [44], and its

A little bit far from your results - avoid digress

receptor tyrosine kinase trkB [45]. Our findings showed that short-term multicomponent exercise training improves both executive and general cognitive functions. This is probably because the multicomponent exercise training program not only improves physical fitness but also enhances motor learning skills, including balance and flexibility. A cross-sectional study suggested that physical and motor fitness were differentially related to cognitive functioning, while physical fitness was mainly related to the executive control process, motor fitness showed a significant association with both the executive control and perceptual speed tasks. Further brain imaging analysis revealed that physical fitness counteracts the effects of aging in the prefrontal and temporal areas. Conversely, motor activity is more likely to enhance visuo-motor coordination and visuo-spatial integration in the parietal area of the brain [14]. Therefore, maintaining an “enriched exercise program” in older age is necessary. Additionally, the most effective modality to improve health-related outcomes in specific population such as adults with overweight/obese is combining different types of exercises during the same session [46]. Strategy for exercise intervention to improve cognitive performance or ameliorate cognitive decline in older women is related to the modality, dose of the exercise, and social support. Combining at least one different type exercise to the aerobic training has more significant effects to the cognition, such as combination of aerobic and resistance [11, 12] or multicomponent exercise [15] as performed in this present study. Regarding the dose of exercise, moderate bouts of exercise duration (> 30–60 min) [11, 12] and moderate intensity [9, 12] on as many days of the week as possible [12] were suggested. Exercise types including cognitively engaging exercises has the most pronounced effect for ameliorating cognitive decline in older adults with mild cognitive impairment [9]. Furthermore, exercise in a group of similar age will increase adherence to exercise in older individuals [47]. To the best of

our knowledge, our study is the first to report the beneficial effects of short-term multicomponent exercise on the executive function of postmenopausal older women.

Strengths and limitations

Punctuation and grammar in this entire paragraph

The strength of the present study include high adherence levels to the exercise program. Herein, we reported the high compliance level of the participants to the exercise program (adherence, >90%). However, monitoring of daily physical activity in the control or exercise group was not conducted using objective tools such as activity-tracking tools. Instead, an initial consent letter was utilized to discourage participants from engaging in additional physical activities. Moreover, encouragement was provided through text messages to each participant in the both group to help them maintain their daily physical activities. Furthermore, our study also reported that a 2-week of multicomponent exercise program improve cognitive performance in postmenopausal women and demonstrated an average moderate to large effect size.

Despite the positive effects we observed, the present study had some limitations. First, the selection of participants into groups was not randomized, which may have influence the heterogeneity of the baseline values of the outcomes. To minimize the negative effects of the nonrandomized design, we recruited age-matched participants as controls and conducted an initial screening of cognitive function using the MMSE score. Second, our results may be confounded by baseline activity levels, health status, and medication used. Regarding the baseline activity levels, none of the participants meet the criteria for active living as outlined in the physical activity guidelines established by the American College of Sports and Medicine for healthy older adults, i.e. five times a week, 30 minutes of moderate intensity physical activity. In terms of health status, we included participants with risk factors for cognitive decline and dementia, such as type 2

diabetes mellitus (T2DM) and hypertension. All of them were taking med[illegible] respective conditions. The study by Bakouni et al. (2019) demonstrated that adherence to antihypertensive medication was associated with a change global cognitive performance. However, there was no indication of a connection between adherence to oral hypoglycemic agents and cognitive health [48]. Although both diseases have been associated with an increased risk of cognitive decline and dementia, a meta-analysis revealed that exercise training in the T2DM population was beneficial for cognitive performance [49], whilst the available randomized controlled trials (RCTs) regarding the effects of physical exercise (PE) on cognitive performance in individuals with hypertension are limited [50]. The aforementioned studies support that there was no strong evidence that these participants experienced reduced cognitive benefits from the exercise intervention. Therefore, further study may be warranted to address the potential differential effects of multicomponent exercises in individuals with type 2 diabetes mellitus (T2DM) and/or hypertension. Third, this study provides insight into the beneficial effects of exercise on cognitive performanc[illegible] postmenopausal older women, and the findings may not be applicable to other populations. Further studies t[illegible]rder to generalize the resul[illegible]

[Just an example of grammar. 'a change in ....']

[Third, our findings are specific to postmenopausal older women and may not be applicable to other populations.]

[Including male participants.]

[There is too much detail on the non-randomised, the authors can simplify a bit this.]

Conclusions

[I think the authors could reshape to better emphasise their findings. It's lacking the link between your hypotheses and the findings. Also, there is no conclusion about the improvement with the training]

This study contributes to the [illegible]t exercise in ~~promoting cognitive function on postmenopausal~~ women. A significant outcome of this study is the marked improvement of some tasks on executive function after the short-term ~~of~~ exercise training. This is also accompanied by the improvement of global cognitive function.

[Repetitive.]

A repetition of the limitations part

Further studies are required and should be directed for longitudinal follow-up in a larger population. This study can potentially be replicated to older men or other population wherein an improvement of cognitive function is needed.

Repetion about the need of replication

Acknowledgment

We extend our gratitude to the participants and Ms. Uswatun Hasanah and Fath., M.D. for their assistance with this study.

Consistency, please

References

1. Reuben R, Karkaby L, McNamee C, Phillips NA, Einstein G. Menopause and cognitive complaints: are ovarian hormones linked with subjective cognitive decline? Climacteric. 2021;24(4):321-32. doi: http://dx.doi.org/10.1080/13697137.2021.1892627.

2. Hogervorst E, Craig J, O'Donnell E. Cognition and mental health in menopause: A review. Best Pract Res Clin Obstet Gynaecol. 2022;81:69-84. doi: https://doi.org/10.1016/j.bpobgyn.2021.10.009.

3. Karlamangla AS, Lachman ME, Han W, Huang M, Greendale GA. Evidence for cognitive aging in midlife women: study of women's health across the nation. PLoS One. 2017;12(1):e0169008. doi: http://dx.doi.org/10.1371/journal.pone.0169008.

4. Launer L, Andersen K, Dewey M, Letenneur L, Ott A, Amaducci L, et al. Rates and risk factors for dementia and Alzheimer's disease: results from EURODEM pooled analyses. Neurology. 1999;52(1):78-. doi: http://dx.doi.org/10.1212/wnl.52.1.78.

5. Bagit A, Hayward GC, MacPherson REK. Exercise and estrogen: common pathways in Alzheimer's disease pathology. Am J Physiol Endocrinol Metab. 2021;321(1):E164-E8. doi: https://doi.org/10.1152/ajpendo.00008.2021. PubMed PMID: 34056921.

6. Sahab-Negah S, Hajali V, Moradi HR, Gorji A. The impact of estradiol on neurogenesis and cognitive functions in Alzheimer's disease. Cell Mol Neurobiol. 2020;40(3):283-99. doi: https://doi.org/10.1007/s10571-019-00733-0.

7. Khadilkar SV, Patil VA. Sex hormones and cognition: where do we stand? J Obstet Gynaecol India. 2019;69:303-12. doi: https://doi.org/10.1007/s13224-019-01223-5.

8. Cheng Y-J, Lin C-H, Lane H-Y. From Menopause to Neurodegeneration—Molecular Basis and Potential Therapy. Int J Mol Sci. 2021;22(16):8654. doi: https://doi.org/10.3390/ijms22168654.

9. Biazus-Sehn LF, Schuch FB, Firth J, de Souza Stigger F. Effects of physical exercise on cognitive function of older adults with mild cognitive impairment: A systematic review and meta-analysis. Arch Gerontol Geriatr. 2020;89:104048. doi: http://dx.doi.org/10.1016/j.archger.2020.104048.

10. Gates NMA, Fiatarone Singh MAMD, Sachdev PSMD, Valenzuela MPD. The effect of exercise training on cognitive function in older adults with mild cognitive impairment: A meta-analysis of randomized controlled trials. Am J Geriatr Psychiatry. 2013;21(11):1086-97. doi: http://dx.doi.org/10.1016/j.jagp.2013.02.018.

11. Colcombe S, Kramer AF. Fitness effects on the cognitive function of older adults : A meta nalytic study. Physiological Science. 2003;14(2):125-30. doi: http://dx.doi.org/10.1111/1467-9280.t01-1-01430.

12. Joseph Michael N, Nicolas C, Kate Louise P, Disa Jane S, Ben R. Exercise interventions for cognitive function in adults older than 50: a systematic review with meta-analysis. Br J Sports Med. 2018;52(3):154-60. doi: http://dx.doi.org/10.1136/bjsports-2016-096587.

13. Vaughan S, Wallis M, Polit D, Steele M, Shum D, Morris N. The effects of multimodal exercise on cognitive and physical functioning and brain-derived neurotrophic factor in older women: a randomised controlled trial. Age Ageing. 2014;43(5):623-9. doi: http://dx.doi.org/10.1093/ageing/afu010.

14. Voelcker‐Rehage C, Godde B, Staudinger UM. Physical and motor fitness are both related to cognition in old age. Eur J Neurosci. 2010;31(1):167-76. doi: http://dx.doi.org/10.1111/j.1460-9568.2009.07014.x.

15. Barha CK, Davis JC, Falck RS, Nagamatsu LS, Liu-Ambrose T. Sex differences in exercise efficacy to improve cognition: A systematic review and meta-analysis of randomized controlled trials in older humans. Front Neuroendocrinol. 2017;46:71-85. doi: https://doi.org/10.1016/j.yfrne.2017.04.002.

16. Baker LD, Frank LL, Foster-Schubert K, Green PS, Wilkinson CW, McTiernan A, et al. Effects of aerobic exercise on mild cognitive impairment: a controlled trial. Arch Neurol. 2010;67(1):71-9. doi: http://dx.doi.org/10.1001/archneurol.2009.307.

17. Middleton L, Kirkland S, Rockwood K. Prevention of CIND by physical activity: different impact on VCI-ND compared with MCI. J Neurol Sci. 2008;269(1-2):80-4. doi: http://dx.doi.org/10.1016/j.jns.2007.04.054.

18. McCarrey AC, An Y, Kitner-Triolo MH, Ferrucci L, Resnick SM. Sex differences in cognitive trajectories in clinically normal older adults. Psychol Aging. 2016;31(2):166-75. doi: https://doi.org/10.1037/pag0000070.

19. Bull FC, Al-Ansari SS, Biddle S, Borodulin K, Buman MP, Cardon G, et al. World Health Organization 2020 guidelines on physical activity and sedentary behaviour. Br J Sports Med. 2020;54(24):1451-62. doi: https://doi.org/10.1136/bjsports-2020-102955. PubMed PMID: 33239350; PubMed Central PMCID: PMCPMC7719906.

20. Kercher VM, Kercher K, Bennion T, Levy P, Alexander C, Amaral PC, et al. 2022 Fitness Trends from Around the Globe.
ACSMs Health Fit J. 2022;26(1):21-37. doi: https://doi.org/10.1249/fit.0000000000000737. PubMed PMID: 00135124-202201000-00007.

21. Etnier JL, Chang Y-K. The effect of physical activity on executive function: a brief commentary on definitions, measurement issues, and the current state of the literature. J Sport Exerc Psychol. 2009;31(4):469-83. doi: https://doi.org/10.1123/jsep.31.4.469.

22. Xu L, Gu H, Cai X, Zhang Y, Hou X, Yu J, et al. The effects of exercise for cognitive function in older adults: A systematic review and meta-analysis of randomized controlled trials. Int J Environ Res Public Health. 2023;20(2). Epub 20230107. doi: https://doi.org/10.3390/ijerph20021088. PubMed PMID: 36673844; PubMed Central PMCID: PMCPMC9858649.

23. Folstein M, Folstein S, McHugh P, Fanjiang G. Mini-Mental State Examination. User's Guide. Odessa, Florida: Psychological Assessment Resources. Inc; 2001.

24. Santos A, Stork MJ, Locke SR, Jung ME. Psychological responses to HIIT and MICT over a 2-week progressive randomized trial among individuals at risk of type 2 diabetes. J Sports Sci. 2021;39(2):170-82. doi: https://doi.org/10.1080/02640414.2020.1809975.

25. Small GW, Silverman DHS, Siddarth P, Ercoli LM, Miller KJ, Lavretsky H, et al. Effects of a 14-day healthy longevity lifestyle program on cognition and brain function. Am J Geriatr Psychiatry. 2006;14(6):538-45. doi: https://doi.org/10.1097/01.JGP.0000219279.72210.ca.

26. Bohnen N, Twijnstra A, Jolles J. Performance in the Stroop color word test in relationship to the persistence of symptoms following mild head injury. Acta Neurol Scand. 1992;85(2):116-21. doi: http://dx.doi.org/10.1111/j.1600-0404.1992.tb04009.x.

27. Sanchia N, Halim MS. Terapi stimulasi kognitif untuk lansia dengan mild cognitive impairment: Studi eksperimental di panti wreda. Neurona. 2019;36(4):258-64. doi: https://doi.org/10.52386/neurona.v36i4.83.

28. Wahyuningrum SE, Sulastri A, Hendriks MPH, Consortium IN, Luijtelaar Gv. The Indonesian neuropsychological test battery (INTB): Psychometric properties, preliminary normative scores, the underlying cognitive construct, and the effects of age and education. Acta Neuropsychol. 2022;20(4):445-70. Epub 2022-11-13. doi: https://doi.org/10.5604/01.3001.0016.1339.

29. Predovan D, Fraser SA, Renaud M, Bherer L. The effect of three months of aerobic training on stroop performance in older adults. J Aging Res. 2012;2012. doi: http://dx.doi.org/10.1155/2012/269815.

30. Ismail R, Mansyur FM, Rahardjo T. Module detection cognitive status. 1st, editor. Jakarta: Center for Ageing Studies University of Indonesia; 2010.

31. Fidiansjah. Efektifitas model penilaian status kognitif warga usia lanjut dan kualitas hidupnya di pelayanan kesehatan primer wilayah pedesaan [Dissertation]. Jakarta: Universitas Indonesia; 2010.

Avoid this type of citation, please, unless it's really essential

32. Project TJ. Jamovi Version 2.3.21 (Computer Software) 2023 [cited 2023 10 March]. Available from: https://www.jamovi.org.

33. Little RJ, Rubin DB. Statistical analysis with missing data: John Wiley & Sons; 2019.

34. Schielzeth H, Dingemanse NJ, Nakagawa S, Westneat DF, Allegue H, Teplitsky C, et al. Robustness of linear mixed-effects models to violations of distributional assumptions. Methods Ecol Evol. 2020;11(9):1141-52. doi: https://doi.org/10.1111/2041-210X.13434.

35. Cohen J. Statistical power analysis for the behavioural sciences. 2nd ed. Hillsdale, New Jersey: Lawrence Erlbaum Associates; 1988. 102 p.

36. Sánchez-González JL, Sánchez-Rodríguez JL, Martín-Vallejo J, Martel-Martel A, González-Sarmiento R. Effects of physical exercise on cognition and telomere length in healthy older women. Brain Sci. 2021;11(11):1417. doi: https://doi.org/10.3390/brainsci11111417.

37. Albinet CT, Abou-Dest A, André N, Audiffren M. Executive functions improvement following a 5-month aquaerobics program in older adults: Role of cardiac vagal control in inhibition performance. Biol Psychol. 2016;115:69-77. doi: https://doi.org/10.1016/j.biopsycho.2016.01.010.

38. Coubard OA, Duretz S, Lefebvre V, Lapalus P, Ferrufino L. Practice of contemporary dance improves cognitive flexibility in aging. Front Aging Neurosci. 2011;3. doi: http://dx.doi.org/10.3389/fnagi.2011.00013.

39. Stern Y. Cognitive reserve. Neuropsychologia. 2009;47(10):2015-28. doi: http://dx.doi.org/10.1016/j.neuropsychologia.2009.03.004.

40. Chodzko-Zajko WJ, Moore KA. Physical fitness and cognitive functioning in aging. Exerc Sport Sci Rev. 1994;22(1):195-220. doi: https://doi.org/10.1249/00003677-199401000-00009.

41. Webster-Cordero F, Giménez-Llort L. The challenge of subjective cognitive complaints and executive functions in middle-aged adults as a preclinical stage of dementia: A systematic review. Geriatrics. 2022;7(2):30. doi: https://doi.org/10.1016/j.exger.2019.01.013.

42. Grober E, Hall CB, Lipton RB, Zonderman AB, Resnick SM, Kawas C. Memory impairment, executive dysfunction, and intellectual decline in preclinical Alzheimer's disease. Journal of the International Neuropsychological Society. 2008;14(2):266-78. doi: https://doi.org/10.1017/S1355617708080302.

43. Erickson KI, Voss MW, Prakash RS, Basak C, Szabo A, Chaddock L, et al. Exercise training increases size of hippocampus and improves memory. Proceedings of the national academy of sciences. 2011;108(7):3017-22. doi: https://doi.org/10.1073/pnas.1015950108.

44. Dinoff A, Herrmann N, Swardfager W, Liu CS, Sherman C, Chan S, et al. The effect of exercise training on resting concentrations of peripheral brain-derived neurotrophic factor (BDNF): A meta-analysis. PloS One. 2016;11(9):e0163037. doi: http://dx.doi.org/10.1016/j.biopsych.2017.02.723.

45. de Melo Coelho FG, Gobbi S, Andreatto CAA, Corazza DI, Pedroso RV, Santos-Galduróz RF. Physical exercise modulates peripheral levels of brain-derived neurotrophic factor (BDNF): a systematic review of experimental studies in the elderly. Arch Gerontol Geriatr. 2013;56(1):10-5. doi: https://doi.org/10.1016/j.archger.2012.06.003.

46. Batrakoulis A, Jamurtas AZ, Metsios GS, Perivoliotis K, Liguori G, Feito Y, et al. Comparative Efficacy of 5 Exercise Types on Cardiometabolic Health in Overweight and Obese Adults: A Systematic Review and Network Meta-Analysis of 81 Randomized Controlled Trials. Circ Cardiovasc Qual Outcomes. 2022;15(6):e008243. doi: https://doi.org/doi:10.1161/CIRCOUTCOMES.121.008243.

47. Beauchamp MR, Ruissen GR, Dunlop WL, Estabrooks PA, Harden SM, Wolf SA, et al. Group-based physical activity for older adults (GOAL) randomized controlled trial: Exercise adherence outcomes. Health Psychology. 2018;37(5):451-61. doi: http://dx.doi.org/10.1037/hea0000615.

48. Bakouni H, Gentil L, Vasiliadis HM. Cognition and drug adherence to oral hypoglycemic and antihypertensive agents in older adults. Patient Prefer Adherence. 2019;13:891-9. Epub 20190530. doi: https://doi.org/10.2147/ppa.S195756. PubMed PMID: 31239647; PubMed Central PMCID: PMCPMC6551588.

49. Cai YH, Wang Z, Feng LY, Ni GX. Effect of Exercise on the Cognitive Function of Older Patients With Type 2 Diabetes Mellitus: A Systematic Review and Meta-Analysis. Front Hum Neurosci. 2022;16:876935. Epub 20220428. doi: https://doi.org/10.3389/fnhum.2022.876935. PubMed PMID: 35572003; PubMed Central PMCID: PMCPMC9096085.

50. Rêgo ML, Cabral DA, Costa EC, Fontes EB. Physical exercise for individuals with hypertension: it is time to emphasize its benefits on the brain and cognition. Clinical Medicine Insights: Cardiology. 2019;13:1179546819839411. doi: https://doi.org/10.1177/1179546819839411.

Supporting information

S1 Table. Exercise intervention.

S1 Table

Click here to access/download
Supporting Information
Suplement 1 Exercise Intervention.docx

Click here to access/download
Supporting Information
Protokol indonesia.pdf

Click here to access/download
Supporting Information
Protocol english.pdf

Click here to access/download

Supporting Information

trendstatement_TREND_Checklist .pdf

Click here to access/download
Other
44 Ethics approval Dani Rahmat Ramadhana.pdf

Clinical trial

Short-term multicomponent exercise training improves executive function in postmenopausal women

Dani Rahmat Ramadhana[1], Rizki Prayuda Putra[1], Michelle Abigail Sibarani[1], Sulistiawati[3], Dewi Ratna Sari[4], Purwo Sri Rejeki [1,2,5], Lilik Herawati [1,2,5], Raden Argarini[1,2,5*]

[1] Master Degree on Sport, Exercise, and Health Sciences, Faculty of Medicine, Airlangga University, Surabaya, Indonesia

[2] Undergraduate Medical Programme, Faculty of Medicine, Airlangga University, Surabaya, Indonesia

[3] Department of Public Health, Faculty of Medicine, Airlangga University, Surabaya, Indonesia

[4] Department of Anatomy, Histology and Pharmacology, Faculty of Medicine, Airlangga University, Surabaya, Indonesia

[5] Department of Medical Physiology and Biochemistry, Faculty of Medicine, Airlangga University, Surabaya, Indonesia

***Corresponding Author:**

Email: raden-a@fk.unair.ac.id (RA)

Trial Registration: ISRCTN13086152

Abstract

Declined cognitive function is commonly complained among older women, during the menopausal transition and continues afterward. Recent research has suggested that combining different exercises stimulates various areas of the brain, potentially leading to greater improvements in cognitive function. However, evidence of the benefits that accrue with multicomponent exercise training, specifically for postmenopausal women is limited. Therefore, this study aimed to investigate the effects of short-term multicomponent exercise training programs on executive function in postmenopausal women. Thirty women (age, 59.8 ± 5.2 years), who were at least 12 months post menopause were allocated into a control (CON) group and an exercise (EX) training group. The EX group underwent a 2-week (five times/week) of multicomponent exercise program comprising aerobic, strength, flexibility, and balance exercises for 40-60 min. Executive function was assessed using the Stroop test and global cognitive function was assessed using the Mini-Mental State Examination (MMSE) at baseline (pre) and after 2 weeks (post) of exercise. Reduced inhibition reaction time of the Stroop test was observed in the EX group (d = -0.78; p = 0.03941). Moreover, reduction of total error was observed in all tasks of the Stroop test following exercise intervention (color naming: d = -0.82, p = 0.032; word reading: d = -0.90, p = 0.01920; inhibition: d = -1.02, p = 0.009; and switching: d = -0.96, p = 0.0124). Additionally, statistical analysis on MMSE score showed a significant improvement (d = 1.31; p = 0.001). In conclusion, our findings suggest that a short-term multicomponent exercise program improves selective tasks of executive function in postmenopausal women along with global cognitive function.

Introduction

In older women, declined cognitive function and mood changes are frequent complaints before, during, and after the menopausal transition. A recent review has identified that cognitive impairment such as lack of concentration, memory issues, planning difficulties, and brain fog, is commonly seen in 62%–67% of women during the menopausal transition [1]. This impairment continues to decline afterwards [2, 3]. Although the evidence of long-term changes in cognitive function during perimenopause and the progression remain unclear, postmenopausal women are more susceptible to a decline in cognitive function and neurodegerative diseases. For example, women have a higher risk of Alzheimer's disease than men [4]. Furthermore, certain cognitive tests including the verbal memory test are gender sensitive [2].

The impact of menopause on the central nervous systems is mostly caused by rapid decline in the production of ovarian hormones, particularly estrogen. Estradiol, the most biologically active type of estrogen, has neuroprotective effects and elicit it effects through genomic and non-genomic signalling pathway to increase brain derived neurotrophic factor (BDNF) [5]. BNDF is responsible for the promotion of neuronal growth, maturation and maintenance as well as synaptic plasticity and memory consolidation [5]. Moreover, estrogen plays a significant role to protect against oxidative stress-induced cell death in hippocampal neurons and interact with the neurotransmitter system, thereby preventing neurodegeneration [6, 7]. Furthermore, it interacts with insulin-like growth factor-1 (IGF-1), which stimulates neuronal cell survival and proliferation [7].

Although estrogen seems to contribute to the cognitive decline during menopausal transition and afterward, the impact of estrogen replacement therapy to cognitive function in postmenopausal women remains inconclusive [8]. It is believed that the complex signalling

pathways and molecular mechanisms of estrogen provide neuroprotective effects. The reduction in BDNF that accompanies the loss of estrogen may play a significant role in cognitive decline in postmenopausal women [5]. Therefore, strategies aimed to increase BDNF levels in postmenopausal woman may be beneficial for maintaining cognitive function in postmenopausal women. Exercise has been proposed as one of the modalities for preventing a rapid decline in cognitive function in older adults. Several systematic reviews with meta-analysis have shown that exercise training in older adults, regardless of their cognitive status, has positive effects on cognitive function [9-12] in at least one cognitive domain. The mechanisms of exercise-induced improvement in cognitive function involve several pathways, including increased brain blood flow and growth of blood vessels, alteration of inflammatory cytokines (reduction in TNF-α and IL-6 concentrations), and increased levels of neurotrophic factors (BDNF) [13], independent of the estrogen-linked BDNF pathway [5]. Advanced research in humans, utilizing functional magnetic resonance imaging [14] reported that alterations in cognitive performance are mediated by changes in neural activation in different areas of the brain. Different types of physical activities are associated with the activation of distinct areas of the brain and cognitive functions [14].

The recent meta-analysis had already established the efficacy of exercise training for enhancing cognitive vitality in older adults; however, some moderator variables may interfere with the positive effect of exercise training on cognition [11, 12, 15]. Some studies [16, 17] and meta-analysis [11, 15] demonstrated that women gain more cognitive benefits from exercise training than men. Additionally, the benefits of exercise on cognition vary depending on the doses of exercise intervention (type, intensity, and duration), the characteristics of the participants at the beginning of the study (gender, general health, and fitness level), and the tasks used for measuring the aspects of cognition [11, 15]. Executive function is a cognitive domain that is mostly affected

by aging. However, this function less declined overtime in women compared to men [18]. Therefore, compared with men, the neural circuitry underlying this domain may remain more intact and may be more sensitive to targeted exercise intervention in women.

A recent study suggested that combining different types of exercises including cardiovascular, resistance, balance, and flexibility exercises can achieve greater gains in cognitive function [13]. Moreover, the World Health Organization strongly suggested older adults to engage in multicomponent exercises to enhance wide range of physical function, prevent falls, manage weight, and increase bone health and prevent osteoporosis [19]. However, evidence of the benefits that accrue with this type of exercise specifically to postmenopausal women is lacking. Furthermore, evidence of the short-term benefits of exercise program to cognitive function is limited. Therefore, this study aimed to investigate the short-term effects of a multicomponent exercise training program comprising aerobic (brisk walking), resistance, flexibility, and balance exercises on executive function together with global cognitive function in postmenopausal women. According to the latest fitness trend, aerobic and resistance training in the form of functional fitness as included in the exercise intervention in this study remain popular worldwide [20]. Several cognitive tasks have been used in studies evaluating the effects of exercise training on cognition, and the Stroop test and Mini-Mental State Examination (MMSE) were particularly selected as neuropsychological assessments to measure executive function and global cognitive function, respectively [21, 22]. The Stroop test is sensitive to exercise training and consists of tasks that allow for the assessment of basic information processing and higher-order aspects of cognition associated with frontal lobes activity [21]. The MMSE is a global clinical, psychological, neuropsychological indicator usually used for screening and evaluating the cognitive status of patients. It can comprehensively, accurately, and quickly reflect the intellectual quality and

cognitive impairment of patients. We hypothesized that we could observe an improvement in executive function in addition to global cognitive function following a short-term multicomponent exercise intervention.

Materials and Methods

Ethics statement and trial registration

This study has been approved by the Ethics Committee of the Faculty of Medicine, Airlangga University, Indonesia (reference number 44/EC/KEPK/FKUA/2023). After a thorough explanation of the testing protocol, the possible risks involved, and the right to terminate participation at anytime, all participants provided informed consent before their enrolment into this study. Participant recruitment started on February 27, 2023 and ended on April 13, 2023. This study was registered in International Standard Randomised Controlled Trial Number (registration number: ISRCTN3086152). This study was prospectively registered as the type intervention (exercise) allows us to register the trial as ongoing/completed study after the first participant enrolment. The authors confirm that all ongoing and related trials for this intervention are registered.

Participants' characteristic

The participants in this study were recruited from women's organizations that participated in the Family Welfare Empowerment Program in Surabaya, East Java, Indonesia. Those who met the initial eligibility requirements (women aged 50-80 years old who are at least 12 months postmenopausal and had a minimum educational level of junior high school) were invited to undergo the next stage of screening. Subsequently, the participants were screened to ensure that

they did not have a history of stroke or any neurological problems, recent surgery, severe hearing or visual impairment, recent cardiovascular diseases (e.g., ischemic heart diseases and heart failure), mental health problems, dementia, or moderate-to-severe cognitive impairment [defined as a score of <21 on the MMSE) [23]. Moreover, to ensure that they were not experiencing moderate-severe stress, psychological stress was assessed using the Depression Anxiety Stress Scale-21 (DASS-21).

Study design

The Consolidated Standards of Reporting Trials (CONSORT) diagram outlining participants' flow form the first contact to the study completion is depicted in Fig 1. Participants who met the inclusion criteria were allocated to the exercise training group (EX, n = 15). Age-matched participants were recruited from the similar women organization and allocated to the control group (CON, n = 15). Participants in the EX group underwent a 2-week exercise program that included a combination of aerobic, strength, balance, and flexibility exercises. Conversely, those in the CON group were asked to continue with their usual activities. The short-duration exercise program was developed to be feasible in the community setting [24]. Additionally, cognitive improvement (verbal fluency test) were observed after short program (14 days) of home-based exercise in combination with diet and relaxation in middle-aged participants (age, 35-69 years) [25]. The outcomes were assessed at baseline and 3 days following the final exercise session. At the initial meeting, all participants were asked to complete a form to collect their baseline information, including educational level, marital status, medical history, and medication. All the measurements were performed in a controlled-temperature room (25℃) at the same time

(06:00 – 08:00 AM). The data analyzer was blinded to the participant allocation. Blinding of participants was not possible in this study.

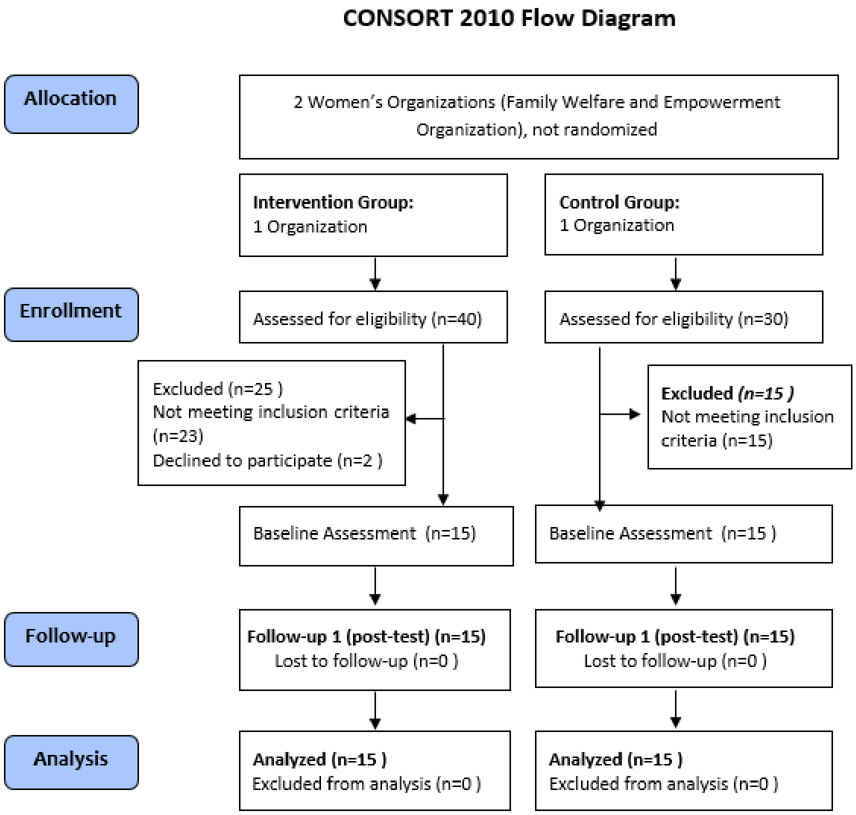

Fig 1. Diagram of participants flow (modified for non-randomized study)

Following enrolment, a pretest was conducted 3 days before the first exercise session, and a posttest was conducted 3 days after the last exercise session. Before outcome measurements, all

participants were asked to fast for 10 h (last meal at 8 PM on the previous day) and refrain from smoking, consuming alcohol or carbonated drinks, and engaging in vigorous exercise. Upon participants' arrival, their blood glucose levels were measured, and they were allowed a light breakfast (2-3 pieces of biscuits, provided by the research team). Resting heart rate and blood pressure were measured two times using an automatic sphygmomanometer (OMRON Model HEM-7156; Omron Healthcare Co., Ltd, Japan). Global cognitive function and the executive functions were assessed using the MMSE and the Stroop test, respectively.

Exercise training

Participants in the EX group attended exercise training five times/week for 2 weeks, every day on weekdays, in a community-based hall. Each exercise session was performed by groups. Each exercise session comprised a combination of aerobic (brisk walking), strength (using loop bands), balance and flexibility exercises. In the first week, each session lasted for 40 min and progressively increased to 60 min. The overall intensity of exercise was moderate (RPE 5-6 of scale 10). The detail of exercise intervention is provided in the S1 Table. The exercise was led by one instructor and two others who assisted and monitored the movements performed by the

participants during the exercise. Participants' attendance was recorded in the logbook, and the adherence rate was calculated on the basis of the number of completed training sessions.

Outcomes measurement

Executive function (Stroop Test)

Executive function was assessed using a modified Stroop test [26] that consists of four different components and is adapted in Bahasa [27, 28]. The intraclass correlation for reaction time and total error was 0.91 and 0.62 respectively [28]. Each component was printed on an A4 sheet of paper and consisted of practice (10 stimuli, five items per line) and test (50 stimuli, 10 items per line). The first condition was word reading, wherein the participants should read the words that were printed using black ink (e.g., red, green, blue, yellow). In the second condition (color naming), the participants should name the color of the rectangles. In the third condition (inhibition), the word was printed in different colors with their meaning. For example, the word "green" was printed using red ink. The participants should name the ink color of the printed words. Lastly, compared to the aforementioned tests, the fourth condition (inhibition/switching) was the most complex test. This test is similar to the third condition (inhibition); however, of 50 items printed color words; 20 were surrounded by a rectangle. In this condition, participants should read the printed words instead of naming the color. Before each test, to ensure their understanding of the task, the participants were provided instructions and an opportunity to practice.,~~,~~ For all conditions, participants should respond as fast as possible and point to which part they are currently on. They were allowed a maximum of four corrections for each test. All tasks were discontinued if the participants had (1) made four uncorrected errors or (2) reached the time limit for each task.

The time limit for tasks 1 and 2 is 90 seconds, while the time limit for tasks 3 and 4 is 180 seconds. The completion testing time (reaction time, ~~-~~RT) was recorded in seconds. ~~and~~ T~~t~~he total number of ~~total~~ errors ~~were noted~~was determined ~~, either~~ by accumulating the ~~amount of~~ corrected and~~or~~ uncorrected errors ~~, were noted and analyzed~~ [29].

Global cognitive health (MMSE)

To assess global cognitive function, including memory, attention, orientation, language, and visuo-spatial ability, we used the Indonesian version of MMSE. The MMSE score ranges from 0 to 30 points; in the Indonesia version, an MMSE score at or below 24 was classified as cognitive dysfunction or dementia. This cutoff point showed a sensitivity and specificity of 88% and 96%, respectively [30]. The Indonesian version MMSE had already undergone validation and reliability test, with Cronbach alpha of 0.87 [31].

Statistical analysis

The sample size calculation was calculated using G*power software version 3.1.9.7 based on the published data of Vaughan *et al*. (2014) [13] which reported the beneficial effects of a 16-week multimodal exercise program (including aerobic, strength, balance, flexibility, coordination, and agility exercises) on cognitive performance in older women. The study reported an effect size of 0.77 for the Stroop test (color naming). The minimum number of subjects required to establish significance is 14 per group would provide an 80% power (α=0.05) for F-test (ANOVA) repeated measures, within-between interaction.

All statistical analysis was computerized using software Jamovi 2.3.21.0 [32]. We performed the analysis of distribution using a visual inspection of Q-Q plot, skewness and kurtosis, and Shapiro Wilk test. The independent t-test was used to compare basic characteristics data of participants, whereas ~~-~~the outcome variables were assessed using linear mixed models. These models enabled the analysis of differences in baseline cognitive performance between the two groups, differences between baseline and post-exercise cognitive performance within each group, and the assessment of whether these differences varied between groups. ~~were used to analyse the relationship between cognitive performance and the exercise training.~~ We defined time (pre vs post) and group (control vs exercise) as fixed factors and participants as random factors to address the individual variations. We tried the linear mixed model within~~in~~ 3 covariance structures: compound symmetry, unstructured, and autoregressive. The smaller the criterion statistics value was~~is~~ based on the Akaike's Information Criterion - Corrected (AICC). Given the close results, the simpler model was~~is~~ chosen~~preferred~~. Therefore, we selected compound symmetry as the underlying covariance structure used in the linear mixed model. Based on that model, we investigated the interaction between time and group and the main effects separately (time: pre vs post and group: CON vs EX). This mixed modelling can deal efficiently with missing data, unbalanced time points [33] and robust with the violations in distributional assumptions [34]. This means that the~~,~~ missing data could be included in the analyses, without imputation. This method also compensates for selective dropout, on the condition that dropout is related to variables included in the models. However, there were no ~~no~~ missing data in this study. In the case of a non-normal distribution of the residuals, a bootstrap procedure was performed with 10,~~.~~000 resamples to obtain reliable standard errors and p-values. The analyses were carried out by the per protocol approach. Only those who attended a minimum of eight exercise sessions (80%) were

included in the post-test. Estimated means, standard deviations and 95% confidence intervals, and the estimation of the interaction between time and treatment in the model are presented. Bonferoni test was used for post-hoc test, with significance level of 95%. Effect sizes of the improvements observed according to Cohen's-d statistic were computed as the absolute change of the outcome measure to its pooled sd. in the group assessed before and after training. Effect size were considered as trivial (<0.2), small (0.2 to 0.49), moderate (0.50 to 0.79) and large (≥0.80) [35]. Graphs were made using PRISM 9.5.1 (Graph Pad Software, La Jolla, CA, USA).

Results

Participant characteristics

Of the 15 participants enrolled in the EX group, the adherence to exercise program was 98.7%. The baseline characteristics of the participants are presented in Table 1. No significant differences in age, body mass index, resting heart rate, and blood pressure were observed (all p > 0.05) (Table 1). Two participants in the EX group were diagnosed with type-2 diabetes mellitus and were treated with oral antidiabetic medication. Three participants (two and one participants in the CON group and EX group, respectively) were diagnosed with hypertension and were treated with an oral antihypertensive medication. Other information on participants characteristics is shown in Table 1.

Table 1. Participant's baseline characteristic

	**Control (n=15)**	**Exercise (n=15)**	**P-Value** (Independent T-test)
Age, year	58.27 ± 5.44	60.27 ± 4.63	0.116
Height, cm	154.00 ± 3.43	152.90 ± 6.50	0.557
Body Weight, kg	59.20 ± 7.36	59.53 ± 9.95	0.919

BMI, kg/m^2	24.90 ± 2.49	25.53 ± 4.28	0.629
Resting heart rate, beats/min	79.53 ± 8.46	81.90 ± 9.29	0.472
Blood pressure			
Systolic blood pressure, mmHg	127.40 ± 18.63	132.30 ± 14.40	0.427
Diastolic blood pressure, mmHg	78.87 ± 10.15	83.63 ± 7.92	0.163
Mean arterial pressure, mmHg	95.01 ± 12.22	99.89 ± 9.47	0.232
FPG mg/dL	117.40 ± 32.90	128.70 ± 57.09	0.515
Education			
Junior high school	0 (0%)	3(20 %)	
Senior high School	7 (46.6%)	10 (66.6 %)	
Diploma	1 (6.6%)	2 (13.3%)	
Bachelor	7 (46.6%)	0 (0%)	
Medical History			
Diabetes mellitus	0 (0%)	2 (13.3%)	
Hypertension	2 (13.3%)	1 (6.6%)	
History of cardiovascular diseases	0 (0%)	0 (0%)	
History of cerebrovascular diseases	0 (0%)	0 (0%)	
Current or ex-smoker	0 (0%)	0 (0%)	
Medication			
Oral antidiabetic	0 (0%)	2 (13.3%)	
Antihypertensive	2 (13.3%)	1 (6.6%)	
Mental Health (DASS-21 Score)			
Depression	1.33 ± 1.39	1.87 ± 3.22	0.564
Anxiety	2.46 ± 1.95	3.40 ± 3.15	0.341
Stress	3.66 ± 2.89	3.13 ± 3.31	0.646

BMI, body mass index; DASS-21, Depression Anxiety Stress Scale-21; FPG, fasting Plasma Glucose; all data are presented as mean ± standard deviation (SDs). *significantly different from control group at $p < 0.05$.

Effects of exercise on cognitive test

Stroop test performance

The results of the Stroop test on completion time performance (reaction time) and total errors before and after exercise are presented in Table 2 and Figs 2 and 3. In the word reading performance, we did not found a significant interaction (p = 0.081) ~~although we found a significant~~or main effects ~~of~~ time (p time = 0.079; p group = 0.410~~37~~). We detected non-significant moderate reduction when comparing differences from baseline of the EX group versus CON group (d = -0.66 (95% CI: -1.41 - 0.11), p = 0.081).The analysis of the total errors in this task revealed a significant interaction (p = 0.019~~20~~) and a main effect of time (p = 0.027). Moreover, we found

significant large reduction of total error in this task when comparing to the baseline in the EX group versus CON group (d = -0.90 (95% CI: -1.68 - 0.10), p = 0.01920).

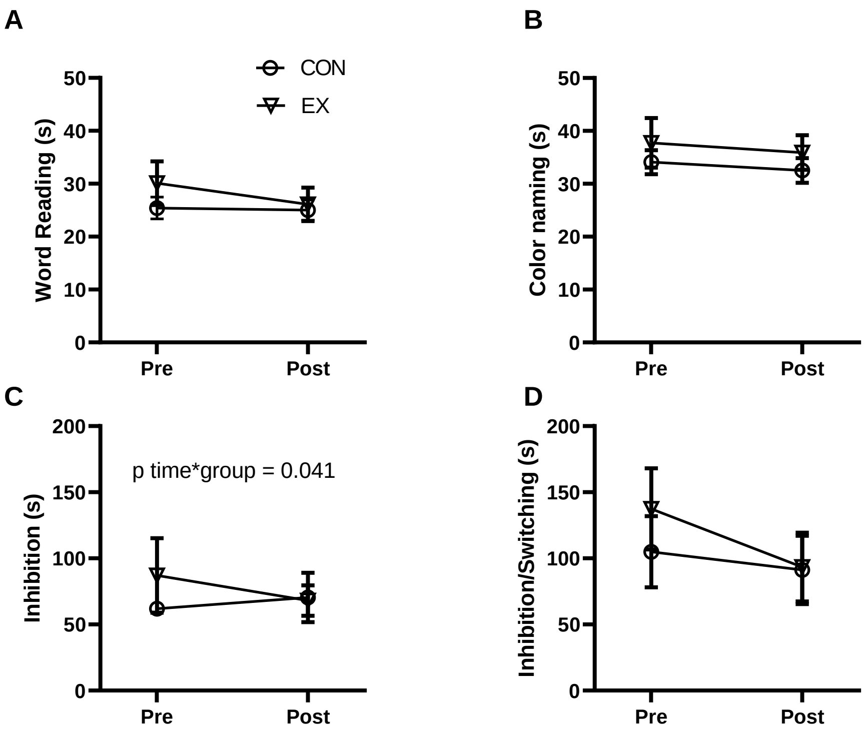
A
B
C
D
CON
EX
Word Reading (s)
Color naming (s)
Inhibition (s)
Inhibition/Switching (s)
p time*group = 0.041
0
10
20
30
40
50
50
100
150
200
Pre
Post

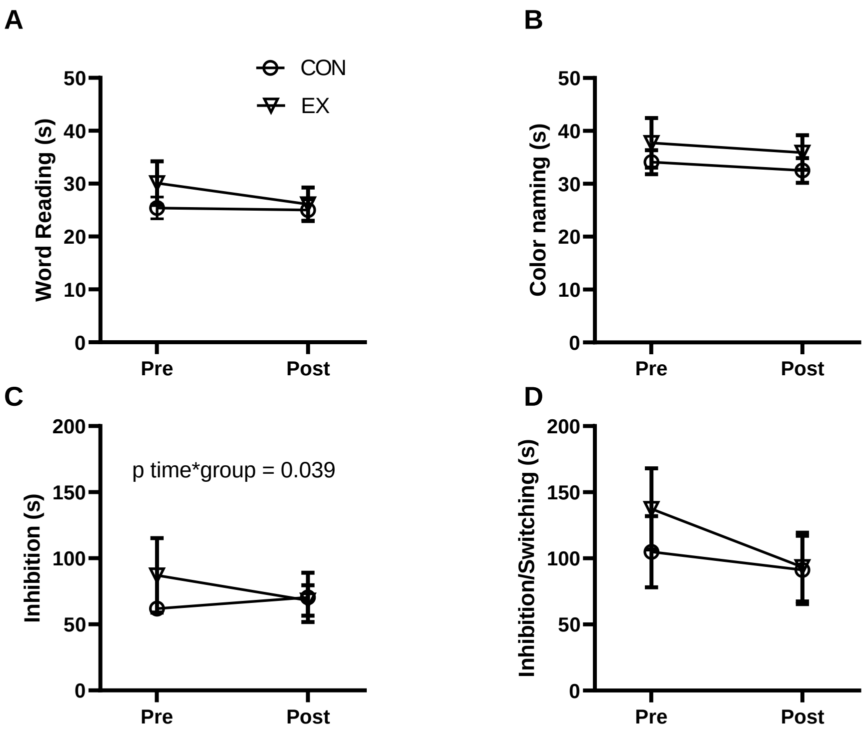

Fig 2~~1~~. Effects of exercise intervention on reaction time of the Stroop test performance in postmenopausal women. Reaction time to complete the word reading task (top, left panel – A), color naming task (top, right panel –B), inhibition task (bottom, left panel – C), and inhibition/switching task (bottom, right panel – D) before (pre) and after (post) 2-weeks. Data is presented as means ± 95% confidence intervals (CI). Effects are derived from linear mixed models, adjusted for age, ~~and~~ years of education, and DASS-21 score.

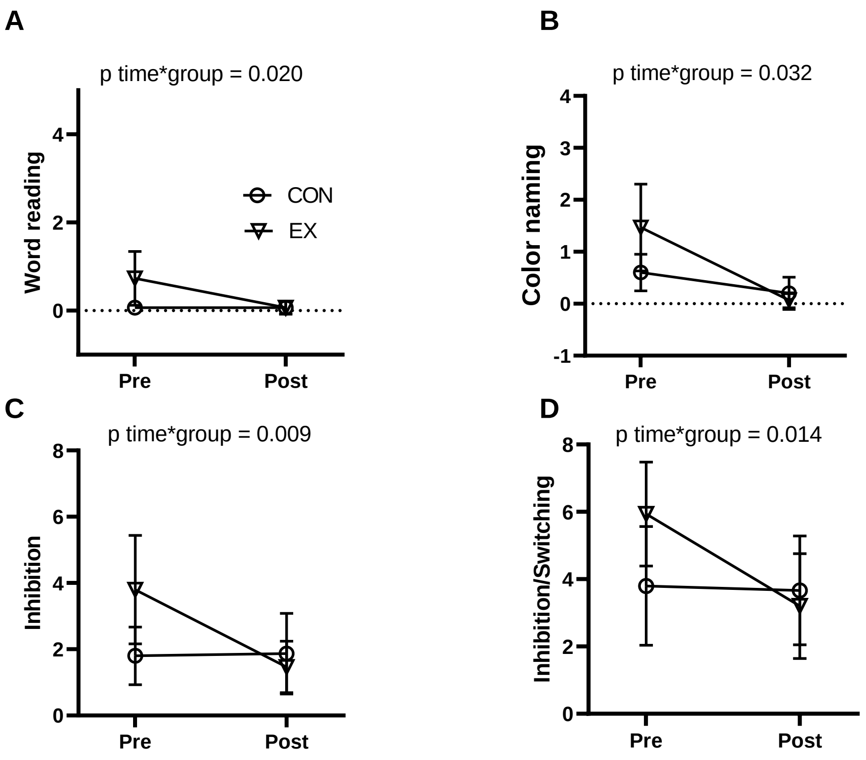
A
p time*group = 0.020
Word reading
CON
EX
Pre
Post
B
p time*group = 0.032
Color naming
Pre
Post
C
p time*group = 0.009
Inhibition
Pre
Post
D
p time*group = 0.014
Inhibition/Switching
Pre
Post

291

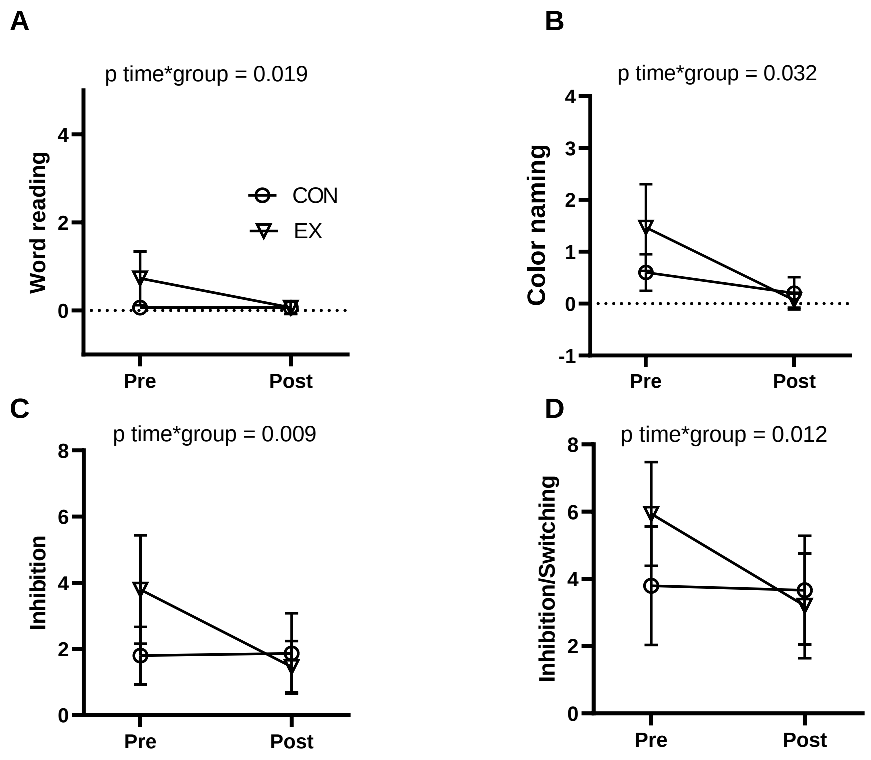

Fig 3~~2~~. Effects of exercise intervention on total errors of the Stroop test in postmenopausal women. Total errors during the completion of the word reading task (top, left panel – A), color naming task (top, right panel –B), inhibition task (bottom, left panel – C), and inhibition/switching task (bottom, right panel – D) before (pre) and after (post) 2-weeks. Data is presented as means ± 95% confidence intervals (CI). Effects are derived from linear mixed models, adjusted for age~~, and~~ years of education, and DASS-21 score.~~.~~

Table 2. Stroop test and MMSE assessment at baseline (pre) and week 2 (post)

	Control (n=15)		Exercise (n=15)		Time*Group		Mean difference from baseline (95% CI)		
	pre mean (95%CI)	post mean (95%CI)	pre mean (95%CI)	post mean (95%CI)	mean (95% CI)	*p*	Control Mean (95%CISE)	Exercise Mean (95%CISE)	ES d
**Stroop test - Reaction time, second**									
Word Reading[a]	25.41 (23.35, 27.46)	25.03 (23.09, 26.96)	30.12 (25.99, 34.24)	26.10 (22.95, 29.25)	-3.5764 (- 7.44520761, 0.5243222)	0.081	-0.038 (1.42)	-3.654.02 (1.432)	-0.660.38
Color naming[a]	34.09 (31.82, 36.36)	32.51 (30.18, 34.83)	37.73 (33.05, 42.42)	35.91 (32.65, 39.17)	-0.224 (-4.463.80964.347, 34.8671343.9656)	0.91607	-1.508 (1.5147)	-1.7283(1.5347)	-0.0435
Inhibition[a]	61.87 (57.05, 66.69)	70.44 (51.82, 89.07)	87.09 (58.93, 115.25)	68.06 (56.61, 79.51)	-27.0861 (-5142.55491872, 2-03.6380181.47)-*	0.03941	8.5710.77 (8.969.12)	-169.3103 (9.0312)	-0.780.37
Inhibition/ Switching[a]	104.99 (78.07, 131.90)	91.19 (65.33, 117.04)	137.41 (106.81, 168.02)	93.42 (67.39, 119.45)	-2930.619 (-69.86752.671.5445, 11.75149.345.49)	0.1686	-113.728 (15.031)	-413.4199 (15.1401)	-0.520.35
**Stroop test - Total Error**									
Word Reading[a]	0.07 (-0.08, 0.21)	0.07 (-0.08, 0.21)	0.73 (0.124 - 1.34)	0.07 (-0.08 - 0.21) †	-0.669967 (-1.186852219 – 0.0141981)-*	0.01920	0.013700 (0.19)	-0.657 (0.19) *	-0.9044
Color naming[a]	0.60 (0.25, 0.95)	0.20 (-0.11, 0.51)	1.47 (0.63 - 2.30)	0.07 (-0.08 - 0.21) †	-0.991 (-1.8759184 – (-0.1590413)-*	0.032	-0.374 (0.31)	-1.374 (0.31) *	-0.820.56
Inhibition	1.80 (0.93, 2.67)	1.87 (0.65, 3.08)	3.80 (2.16 - 5.44)	1.47 (0.69 - 2.25) †	-2.384 (-4.0738 – (-0.6872)-*	0.009	0.170.67 (0.601)	-2.213 (0.61) *	-10.0239
Switching	3.80 (2.03, 5.57)	5.93 (2.05, 5.28)	3.67 (4.39 - 7.48)	3.20 (1.64 - 4.76) †	-2.556 (-4.46154 – (-0.6486)-*	0.0124	-0.0913(0.6970)	-2.4673 (0.6970)-*	-0.9637
**MMSE score (0-30)**	25.33 (24.03, 26.63)	26.47 (25.63 – 27.30)	24.20 (22.82 – 25.58)	28.47 (27.63 – 29.30) †	3.113 (1.37412 – 4.8515)-*	0.001	1.0413 (0.62)	4.1527 (0.632) *	1.310.62

Values are from linear mixed effects models adjusted for age, ~~and~~ years of education, and DASS-21 score.
*Significant time*treatment effect ($p < 0.05$)
[†]Significant time effect ($p < 0.05$) compared with baseline (pre).
[a]Bootstrap for non-normal distribution of the residuals
ES: Effect size

Regarding color naming reaction time, a significant interaction or main effect was not observed (all p > 0.05). We found a non-significant trivial reduction when comparing differences from baseline of the EX group versus CON group (d = -0.04 (95% CI: -0.76 - 0.67), p = 0.916~~07~~, Table 2 and Fig 2). In the total errors, we found a significant interaction (p = 0.032) and a time effect (p < 0.001) without group effect (p = 0. 315~~281~~). Additionally, we detected a large significant reduction when comparing differences from baseline of the EX group versus CON group (d = -0.82 (95% CI: -1.59 - 0.04), p = 0.032~~0~~).

In the inhibition task, we found that both reaction time and total errors have a significant interaction (p = 0.039~~41~~, p = 0.009). Additionally, we observed significant large reduction when comparing differences from baseline of the EX group versus CON group of the reaction time (d = -0.78 (95% CI: -1.54 - 0.00), p = 0.039~~41~~) and total errors (d = -1.02 (95% CI: -1.82, - 0.20), p = 0.009).

In the inhibition/switching task, the interaction for the reaction time (p = 0.168~~6~~) was not significant although the main effect of time showed significant results (p = 0.020~~11~~). We observed non-significant moderate reduction when comparing differences from baseline of the EX group versus CON group of the reaction time (d = -0.52 (95% CI: -1.25 - 0.23), p = 0.168~~6~~). Additionally, the statistical analysis of the total errors of the IN/SW task showed a significant interaction (p = 0.012~~1~~2~~4~~) and a main effect of time (p = 0.024~~07~~). In line with this, we detected significant large reduction when comparing differences from baseline of the EX group versus CON group of the reaction time (d = -0.96 (95% CI: -1.74, - 0.15), p = 0.012~~4~~).

The effect of exercise on global cognitive function (Mini Mental State Examinations - MMSE)

The analysis of MMSE showed a significant interaction time*group (p = 0.001) and a main effect of time (p < 0.001) as presented in (Fig 43). Furthermore, we observed significant large improvement of MMSE score in when comparing differences from baseline of the EX group versus CON group (d = 1.31(95% CI: 0.43 – 2.15), p = 0.001).

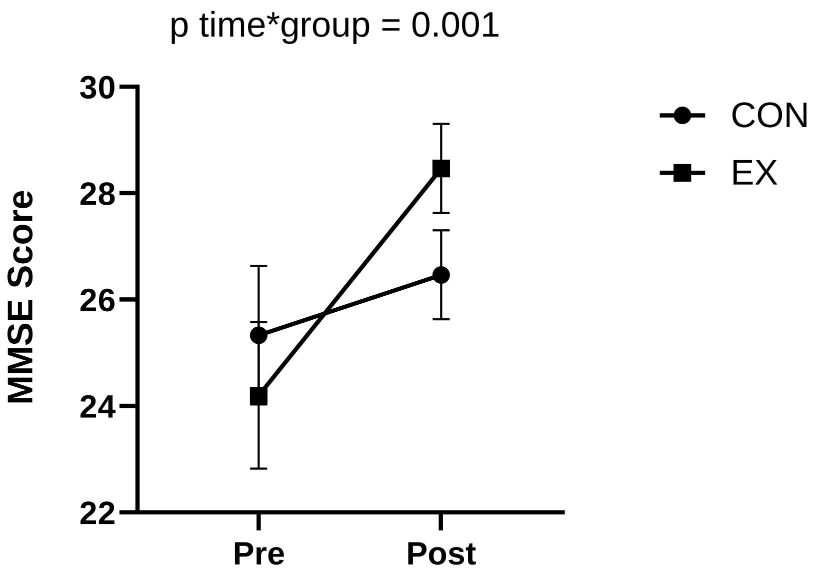

Fig 43. Effects of exercise intervention on MMSE in postmenopausal women. The graph shows MMSE scores before (pre) and after (post) 2-weeks of exercise training in the exercise group and the control group. Data is presented as means ± 95% confidence intervals (CI). Effects are derived from linear mixed models, adjusted for age, and years of education and DASS-21 score.

Discussion

The multicomponent of exercise training programs aimed to improve physical fitness and develop motor learning skills, including balance and flexibility. The main findings of this study include a short-term of multicomponent exercise training program improved inhibition task performance of the Stroop test. We noted a positive trend towards improvement of the reaction time of other components of the Stroop task (color naming, word reading, and switching) although did not reach statistical significance. Moreover, we observed the improvement in accuracy to perform the Stroop test in all tasks as shown in the reduction of total errors. Consistent with our results, Vaughan et al. (2014) investigated the effects of a 16-week multicomponent exercise that includes aerobic-strength-motor fitness (balance, coordination, flexibility, and agility) in older women. The results demonstrated moderate-to-large effect size of exercise to improve the reaction time of all tasks of the California Older Stroop Test, including color naming, word reading and inhibition task [13]. In contrast, another study demonstrated no significant effect in all components of the Stroop tasks following a 6-month of multicomponent exercise program in older women [36]. However, this study used a different analysis of the Stroop test by the total number of correct words in 45 s instead of the reaction time and total error [36]. Most studies that investigated the effect of exercise employed aerobic exercises. Predovan et al (2012) evaluated executive functions after a 12-week of aerobic training in older adults (>70% women participants) and noted differences only in the switching condition. Similarly, significant improvement in total error was observed only in the switching component [29]. Another study of aquarobics program in older adults (>70% women participants) showed improvement in the Stroop test performance after 21 weeks but not in shorter-term intervention (10 weeks) [37]. Although generally longer period of exercise intervention provide more benefit in cognitive function, Colcombe and Kramer (2003) in their meta-analysis

showed that shorter duration (4-12 weeks) lead to better cognitive improvement than medium duration (12-24 weeks). Additionally, this meta-analysis showed that combined aerobic and resistance exercise demonstrated larger effect compared to aerobic alone [11].

A possible explanation of the discrepancy of our results with those of other studies is the differences in the modality and doses of exercises. The effect of physical exercise on cognitive function has been proven to be task-specific and motor activity specific [38]. Stern (2009) hypothesized that a higher cognitive reserve resulting from physical exercise is because of a neural compensation mechanism that permits complex activities [39]. Complex tasks such as inhibition and inhibition/switching in the Stroop test require multiple executive processes. Chodzko-Zajko et al. (1994) suggested that tasks requiring more control and effortful processing including the Stroop test, should be more sensitive to fitness differences among older adults than tasks that can be processed automatically [40].

In this study, we estimated a moderate effect of exercise on the speed improvement of the inhibition task of the Stroop test and a larger effect on the accuracy of all tasks in the postmenopausal women population. Our positive findings on executive function suggest that exercise has a beneficial effect on controlling functions critical for processes involved in cognitive learning. These functions include information processing speed, attention control, resistance to interference, dual-tasking, and cognitive flexibility [13]. From a clinical perspective, a 15-year prospective study on individuals with Alzheimer's disease revealed that lower cognitive performance at the executive level began to show more pronounced signs 2–3 years before receiving a clinical diagnosis [41]. In addition, rRecent systematic review research has suggested a significant cognitive decline in executive processes during progressive neuropsychological follow-ups, leading to a potential manifestation of mild cognitive impairment (MCI) or dementia

[41]. From a clinical perspective, a 15-year prospective study on individuals with Alzheimer's disease revealed that lower cognitive performance at the executive level began to show more pronounced signs 2–3 years before receiving a clinical diagnosis [42]. Thus, monitoring the performance of executive function tasks and developing interventions to maintain or improve these cognitive functions is necessary to address the epidemic of dementia and other cognitive disorders. However, the generalization of our findings should be approached with caution as our study~~y~~ focused on post-menopausal older women, a group that may be psychologically vulnerable [4] and ~~could~~may potentially experience more benefit on executive processes from exercise than men [15]~~cognitive benefits from exercise in specific cognitive domains such as motor function, cognitive speed, and auditory and visual attention~~ . In addition, the executive function as the primary outcome of this study is one of cognitive domains that is commonly benefited positively from exercise interventions. Similar intervention might have different effect on other cognitive domains such as ~~attention~~verbal fluency.

The second aim of our study was to investigate the global cognitive function using the MMSE. Regarding the MMSE score, our results were consistent with those of Sanchez-Gonzalez et al. (2021). However, this study showed little effect on the MMSE score improvement. This may be because the participants in this group have already high MMSE scores at baseline (mean score > 27) [36]. This test is believed to have a ceiling effect because the highest score is 30. Moreover, our findings are consistent with the results of a meta-analysis study that showed most exercise intervention improve global cognitive function in older individuals [9].

Our study did not investigate the mechanism of cognitive function improvement as a result of exercise intervention. However, several studies have shown that physical exercise improves

brain functioning through both structural and functional changes. Studies have reported that exercise training can lead to an increase in gray and white matter volume in the prefrontal cortex and hippocampal regions [43]. The prefrontal area is important to process the executive function and this cognitive task has the most pronounced effect because of exercise training [11]. Moreover, it has been observed to increase the levels of neurotrophic factors, such as BDNF [44], and its receptor tyrosine kinase trkB [45]. Our findings showed that short-term multicomponent exercise training improves both executive and general cognitive functions. This is probably because the multicomponent exercise training program not only improves physical fitness but also enhances motor learning skills, including balance and flexibility. A cross-sectional study suggested that physical and motor fitness were differentially related to cognitive functioning, while physical fitness was mainly related to the executive control process, motor fitness showed a significant association with both the executive control and perceptual speed tasks. Further brain imaging analysis revealed that physical fitness counteracts the effects of aging in the prefrontal and temporal areas. Conversely, motor activity is more likely to enhance visuo-motor coordination and visuo-spatial integration in the parietal area of the brain [14]. Therefore, maintaining an "enriched exercise program" in older age is necessary. Additionally, the most effective modality to improve health-related outcomes in specific population such as adults with overweight/obese is combining different types of exercises during the same session [46]. Strategy for exercise intervention to improve cognitive performance or ameliorate cognitive decline in older women is related to the modality, dose of the exercise, and social support. Combining at least one different type exercise to the aerobic training has more significant effects to the cognition, such as combination of aerobic and resistance [11, 12] or multicomponent exercise [15] as performed in this present study. Regarding the dose of exercise, moderate bouts of exercise duration (> 30–60 min) [11, 12] and

moderate intensity [9, 12] on as many days of the week as possible [12] were suggested. Exercise types including cognitively engaging exercises has the most pronounced effect for ameliorating cognitive decline in older adults with mild cognitive impairment [9]. Furthermore, exercise in a group of similar age will increase adherence to exercise in older individuals [47]. To the best of our knowledge, our study is the first to report the beneficial effects of short-term multicomponent exercise on the executive function of postmenopausal older women.

Strengths and limitations

The strength of the present study include high adherence levels to the exercise program. Herein, we reported the high compliance level of the participants to the exercise program (adherence, >90%). However, monitoring of daily physical activity in the control or exercise group was not conducted using objective tools such as activity-tracking tools. Instead, an initial consent letter was utilized to discourage participants from engaging in additional physical activities. Moreover, encouragement was provided through text messages to each participant in the both group to help them maintain their daily physical activities. Furthermore, our study also reported that a 2-week of multicomponent exercise program improve cognitive performance in postmenopausal women and demonstrated an average moderate to large effect size.

Despite the positive effects we observed, the present study had some limitations. First, t~~T~~he selection of participants into groups was not randomized, which may have influence the heterogeneity of the baseline values of the outcomes. To minimize the negative effects of the nonrandomized design, we recruited age-matched participants as controls and conducted an initial screening of cognitive function using the MMSE score. ~~Additionally~~ Second, our results may be confounded by baseline activity levels, health status, and medication used. Regarding the baseline

activity levels, nNHowever, none of theme participants meet the criteria for active living as outlined in the physical activity guidelines established by the American College of Sports and Medicine for healthy older adults, i.e. five times a week, 30 minutes of moderate intensity physical activity. In additionIn terms of health status, we included participants with risk factors for cognitive decline and dementia, such as type 2 diabetes mellitus (T2DM) and hypertension. All of them were taking medications for their respective conditions. The study by Bakouni et al. (2019) demonstrated that adherence to antihypertensive medication was associated with a change global cognitive performance{Bakouni, 2019 #1289}. However, there was no indication of a connection between adherence to oral hypoglycemic agents and cognitive health [48]. , such as antihypertensive and oral hypoglycemic drugs. Although both diseases have been associated with an increased risk of cognitive decline and dementia, a meta-analysis revealed that exercise training in the T2DM population was beneficial for cognitive performance [49], whilst the available randomized controlled trials (RCTs) regarding the effects of physical exercise (PE) on cognitive performance in individuals with hypertension are limited [50]. {Fujii, 2020 #933}. The study by Bakouni et al. (2019) demonstrated that adherence to antihypertensive medication was associated with a change in MMSE scores. However, there was no indication of a connection between adherence to oral hypoglycemic agents and cognitive health. Although The aforementioned studies support that there wasis no strong evidence that these participants experienced reduced cognitive benefits from the exercise intervention,. Therefore, ffurther study may be warranted to address the potential differential effects of multicomponent exercises in individuals with type 2 diabetes mellitus (T2DM) and/or hypertension. Third, this study provides insight into the beneficial effects of short-term multicomponent exercise on cognitive performance in postmenopausal older women,

and the findings may not be applicable to other populations. Further studies that include male participants are needed in order to generalize the results to the overall aging population.

Third, the results of this study can only be generalized to postmenopausal older women, and the application to other populations may not haveyield similar results.

Second, monitoring of daily physical activity in the control or exercise group was not conducted using logbooks or activity-tracking tools. Instead, an initial consent letter was utilized to discourage participants from engaging in additional physical activities. Moreover, encouragement was provided through text messages to each participant in the controlboth group to help them maintain their daily physical activities. Additionally, monitoring of physical activity in the control group was not performed although we suggested to the participant to maintain their daily physical activities. LastlyThird, the results of this study can only be generalized to postmenopausal older women, and the application to other population may not have similar results. . Forth, other potential confounder such as baseline activity levels and medication use. However, none of them meet the active criteria according to the physical activity guidelines set by the American College of Sports and Medicine for healthy older adults, i.e. five times a week, 30 minutes of moderate intensity physical activity. In addition, .

Formatted: English (United Kingdom)

Conclusions

This study contributes to the understanding of the beneficial role of multicomponent exercise in promoting cognitive function on postmenopausal women. A significant outcome of this study is the marked improvement of some tasks on executive function after the short-term of

exercise training. This is also accompanied by the improvement of global cognitive function. Further studies are required and should be directed for longitudinal follow-up in a larger population. This study can potentially also be replicated to older men or other population wherein an improvement of cognitive function is needed.

Acknowledgment

We extend our gratitude to the participants and Ms. Uswatun Hasanah and Fath., M.D. for their assistance with this study.

References

1. Reuben R, Karkaby L, McNamee C, Phillips NA, Einstein G. Menopause and cognitive complaints: are ovarian hormones linked with subjective cognitive decline? Climacteric. 2021;24(4):321-32. doi: http://dx.doi.org/10.1080/13697137.2021.1892627.

2. Hogervorst E, Craig J, O'Donnell E. Cognition and mental health in menopause: A review. Best Pract Res Clin Obstet Gynaecol. 2022;81:69-84. doi: https://doi.org/10.1016/j.bpobgyn.2021.10.009.

3. Karlamangla AS, Lachman ME, Han W, Huang M, Greendale GA. Evidence for cognitive aging in midlife women: study of women's health across the nation. PLoS One. 2017;12(1):e0169008. doi: http://dx.doi.org/10.1371/journal.pone.0169008.

4. Launer L, Andersen K, Dewey M, Letenneur L, Ott A, Amaducci L, et al. Rates and risk factors for dementia and Alzheimer's disease: results from EURODEM pooled analyses. Neurology. 1999;52(1):78-. doi: http://dx.doi.org/10.1212/wnl.52.1.78.

5. Bagit A, Hayward GC, MacPherson REK. Exercise and estrogen: common pathways in Alzheimer's disease pathology. Am J Physiol Endocrinol Metab. 2021;321(1):E164-E8. doi: https://doi.org/10.1152/ajpendo.00008.2021. PubMed PMID: 34056921.

6. Sahab-Negah S, Hajali V, Moradi HR, Gorji A. The impact of estradiol on neurogenesis and cognitive functions in Alzheimer's disease. Cell Mol Neurobiol. 2020;40(3):283-99. doi: https://doi.org/10.1007/s10571-019-00733-0.

7. Khadilkar SV, Patil VA. Sex hormones and cognition: where do we stand? J Obstet Gynaecol India. 2019;69:303-12. doi: https://doi.org/10.1007/s13224-019-01223-5.

8. Cheng Y-J, Lin C-H, Lane H-Y. From Menopause to Neurodegeneration—Molecular Basis and Potential Therapy. Int J Mol Sci. 2021;22(16):8654. doi: https://doi.org/10.3390/ijms22168654.

9. Biazus-Sehn LF, Schuch FB, Firth J, de Souza Stigger F. Effects of physical exercise on cognitive function of older adults with mild cognitive impairment: A systematic review and meta-analysis. Arch Gerontol Geriatr. 2020;89:104048. doi: http://dx.doi.org/10.1016/j.archger.2020.104048.

10. Gates NMA, Fiatarone Singh MAMD, Sachdev PSMD, Valenzuela MPD. The effect of exercise training on cognitive function in older adults with mild cognitive impairment: A meta-analysis of randomized controlled trials. Am J Geriatr Psychiatry. 2013;21(11):1086-97. doi: http://dx.doi.org/10.1016/j.jagp.2013.02.018.

11. Colcombe S, Kramer AF. Fitness effects on the cognitive function of older adults : A meta nalytic study. Physiological Science. 2003;14(2):125-30. doi: http://dx.doi.org/10.1111/1467-9280.t01-1-01430.

12. Joseph Michael N, Nicolas C, Kate Louise P, Disa Jane S, Ben R. Exercise interventions for cognitive function in adults older than 50: a systematic review with meta-analysis. Br J Sports Med. 2018;52(3):154-60. doi: http://dx.doi.org/10.1136/bjsports-2016-096587.

13. Vaughan S, Wallis M, Polit D, Steele M, Shum D, Morris N. The effects of multimodal exercise on cognitive and physical functioning and brain-derived neurotrophic factor in older women: a randomised controlled trial. Age Ageing. 2014;43(5):623-9. doi: http://dx.doi.org/10.1093/ageing/afu010.

14. Voelcker‐Rehage C, Godde B, Staudinger UM. Physical and motor fitness are both related to cognition in old age. Eur J Neurosci. 2010;31(1):167-76. doi: http://dx.doi.org/10.1111/j.1460-9568.2009.07014.x.

15. Barha CK, Davis JC, Falck RS, Nagamatsu LS, Liu-Ambrose T. Sex differences in exercise efficacy to improve cognition: A systematic review and meta-analysis of randomized controlled trials in older humans. Front Neuroendocrinol. 2017;46:71-85. doi: https://doi.org/10.1016/j.yfrne.2017.04.002.

16. Baker LD, Frank LL, Foster-Schubert K, Green PS, Wilkinson CW, McTiernan A, et al. Effects of aerobic exercise on mild cognitive impairment: a controlled trial. Arch Neurol. 2010;67(1):71-9. doi: http://dx.doi.org/10.1001/archneurol.2009.307.

17. Middleton L, Kirkland S, Rockwood K. Prevention of CIND by physical activity: different impact on VCI-ND compared with MCI. J Neurol Sci. 2008;269(1-2):80-4. doi: http://dx.doi.org/10.1016/j.jns.2007.04.054.

18. McCarrey AC, An Y, Kitner-Triolo MH, Ferrucci L, Resnick SM. Sex differences in cognitive trajectories in clinically normal older adults. Psychol Aging. 2016;31(2):166-75. doi: https://doi.org/10.1037/pag0000070.

19. Bull FC, Al-Ansari SS, Biddle S, Borodulin K, Buman MP, Cardon G, et al. World Health Organization 2020 guidelines on physical activity and sedentary behaviour. Br J Sports Med. 2020;54(24):1451-62. doi: https://doi.org/10.1136/bjsports-2020-102955. PubMed PMID: 33239350; PubMed Central PMCID: PMCPMC7719906.

20. Kercher VM, Kercher K, Bennion T, Levy P, Alexander C, Amaral PC, et al. 2022 Fitness Trends from Around the Globe. ACSMs Health Fit J. 2022;26(1):21-37. doi: https://doi.org/10.1249/fit.0000000000000737. PubMed PMID: 00135124-202201000-00007.

21. Etnier JL, Chang Y-K. The effect of physical activity on executive function: a brief commentary on definitions, measurement issues, and the current state of the literature. J Sport Exerc Psychol. 2009;31(4):469-83. doi: https://doi.org/10.1123/jsep.31.4.469.

22. Xu L, Gu H, Cai X, Zhang Y, Hou X, Yu J, et al. The effects of exercise for cognitive function in older adults: A systematic review and meta-analysis of randomized controlled trials. Int J Environ Res Public Health. 2023;20(2). Epub 20230107. doi: https://doi.org/10.3390/ijerph20021088. PubMed PMID: 36673844; PubMed Central PMCID: PMCPMC9858649.

23. Folstein M, Folstein S, McHugh P, Fanjiang G. Mini-Mental State Examination. User's Guide. Odessa, Florida: Psychological Assessment Resources. Inc; 2001.

24. Santos A, Stork MJ, Locke SR, Jung ME. Psychological responses to HIIT and MICT over a 2-week progressive randomized trial among individuals at risk of type 2 diabetes. J Sports Sci. 2021;39(2):170-82. doi: https://doi.org/10.1080/02640414.2020.1809975.

25. Small GW, Silverman DHS, Siddarth P, Ercoli LM, Miller KJ, Lavretsky H, et al. Effects of a 14-day healthy longevity lifestyle program on cognition and brain function. Am J Geriatr Psychiatry. 2006;14(6):538-45. doi: https://doi.org/10.1097/01.JGP.0000219279.72210.ca.

26. Bohnen N, Twijnstra A, Jolles J. Performance in the Stroop color word test in relationship to the persistence of symptoms following mild head injury. Acta Neurol Scand. 1992;85(2):116-21. doi: http://dx.doi.org/10.1111/j.1600-0404.1992.tb04009.x.

27. Sanchia N, Halim MS. Terapi stimulasi kognitif untuk lansia dengan mild cognitive impairment: Studi eksperimental di panti wreda. Neurona. 2019;36(4):258-64. doi: https://doi.org/10.52386/neurona.v36i4.83.

28. Wahyuningrum SE, Sulastri A, Hendriks MPH, Consortium IN, Luijtelaar Gv. The Indonesian neuropsychological test battery (INTB): Psychometric properties, preliminary normative scores, the underlying cognitive construct, and the effects of age and education. Acta Neuropsychol. 2022;20(4):445-70. Epub 2022-11-13. doi: https://doi.org/10.5604/01.3001.0016.1339.

29. Predovan D, Fraser SA, Renaud M, Bherer L. The effect of three months of aerobic training on stroop performance in older adults. J Aging Res. 2012;2012. doi: http://dx.doi.org/10.1155/2012/269815.

30. Ismail R, Mansyur FM, Rahardjo T. Module detection cognitive status. 1st, editor. Jakarta: Center for Ageing Studies University of Indonesia; 2010.

31. Fidiansjah. Efektifitas model penilaian status kognitif warga usia lanjut dan kualitas hidupnya di pelayanan kesehatan primer wilayah pedesaan [Dissertation]. Jakarta: Universitas Indonesia; 2010.

32. Project TJ. Jamovi Version 2.3.21 (Computer Software) 2023 [cited 2023 10 March]. Available from: https://www.jamovi.org.

33. Little RJ, Rubin DB. Statistical analysis with missing data: John Wiley & Sons; 2019.

34. Schielzeth H, Dingemanse NJ, Nakagawa S, Westneat DF, Allegue H, Teplitsky C, et al. Robustness of linear mixed-effects models to violations of distributional assumptions. Methods Ecol Evol. 2020;11(9):1141-52. doi: https://doi.org/10.1111/2041-210X.13434.

35. Cohen J. Statistical power analysis for the behavioural sciences. 2nd ed. Hillsdale, New Jersey: Lawrence Erlbaum Associates; 1988. 102 p.

36. Sánchez-González JL, Sánchez-Rodríguez JL, Martín-Vallejo J, Martel-Martel A, González-Sarmiento R. Effects of physical exercise on cognition and telomere length in healthy older women. Brain Sci. 2021;11(11):1417. doi: https://doi.org/10.3390/brainsci11111417.

37. Albinet CT, Abou-Dest A, André N, Audiffren M. Executive functions improvement following a 5-month aquaerobics program in older adults: Role of cardiac vagal control in inhibition performance. Biol Psychol. 2016;115:69-77. doi: https://doi.org/10.1016/j.biopsycho.2016.01.010.

38. Coubard OA, Duretz S, Lefebvre V, Lapalus P, Ferrufino L. Practice of contemporary dance improves cognitive flexibility in aging. Front Aging Neurosci. 2011;3. doi: http://dx.doi.org/10.3389/fnagi.2011.00013.

39. Stern Y. Cognitive reserve. Neuropsychologia. 2009;47(10):2015-28. doi: http://dx.doi.org/10.1016/j.neuropsychologia.2009.03.004.

40. Chodzko-Zajko WJ, Moore KA. Physical fitness and cognitive functioning in aging. Exerc Sport Sci Rev. 1994;22(1):195-220. doi: https://doi.org/10.1249/00003677-199401000-00009.

41. Webster-Cordero F, Giménez-Llort L. The challenge of subjective cognitive complaints and executive functions in middle-aged adults as a preclinical stage of dementia: A systematic review. Geriatrics. 2022;7(2):30. doi: https://doi.org/10.1016/j.exger.2019.01.013.

42. Grober E, Hall CB, Lipton RB, Zonderman AB, Resnick SM, Kawas C. Memory impairment, executive dysfunction, and intellectual decline in preclinical Alzheimer's disease. Journal of the International Neuropsychological Society. 2008;14(2):266-78. doi: https://doi.org/10.1017/S1355617708080302.

43. Erickson KI, Voss MW, Prakash RS, Basak C, Szabo A, Chaddock L, et al. Exercise training increases size of hippocampus and improves memory. Proceedings of the national academy of sciences. 2011;108(7):3017-22. doi: https://doi.org/10.1073/pnas.1015950108.

44. Dinoff A, Herrmann N, Swardfager W, Liu CS, Sherman C, Chan S, et al. The effect of exercise training on resting concentrations of peripheral brain-derived neurotrophic factor (BDNF): A meta-analysis. PloS One. 2016;11(9):e0163037. doi: http://dx.doi.org/10.1016/j.biopsych.2017.02.723.

45. de Melo Coelho FG, Gobbi S, Andreatto CAA, Corazza DI, Pedroso RV, Santos-Galduróz RF. Physical exercise modulates peripheral levels of brain-derived neurotrophic factor (BDNF): a systematic review of experimental studies in the elderly. Arch Gerontol Geriatr. 2013;56(1):10-5. doi: https://doi.org/10.1016/j.archger.2012.06.003.

46. Batrakoulis A, Jamurtas AZ, Metsios GS, Perivoliotis K, Liguori G, Feito Y, et al. Comparative Efficacy of 5 Exercise Types on Cardiometabolic Health in Overweight and Obese Adults: A Systematic Review and Network Meta-Analysis of 81 Randomized Controlled Trials. Circ Cardiovasc Qual Outcomes. 2022;15(6):e008243. doi: https://doi.org/doi:10.1161/CIRCOUTCOMES.121.008243.

47. Beauchamp MR, Ruissen GR, Dunlop WL, Estabrooks PA, Harden SM, Wolf SA, et al. Group-based physical activity for older adults (GOAL) randomized controlled trial: Exercise adherence outcomes. Health Psychology. 2018;37(5):451-61. doi: http://dx.doi.org/10.1037/hea0000615.

48. Bakouni H, Gentil L, Vasiliadis HM. Cognition and drug adherence to oral hypoglycemic and antihypertensive agents in older adults. Patient Prefer Adherence. 2019;13:891-9. Epub 20190530. doi: https://doi.org/10.2147/ppa.S195756. PubMed PMID: 31239647; PubMed Central PMCID: PMCPMC6551588.

49. Cai YH, Wang Z, Feng LY, Ni GX. Effect of Exercise on the Cognitive Function of Older Patients With Type 2 Diabetes Mellitus: A Systematic Review and Meta-Analysis. Front Hum Neurosci. 2022;16:876935. Epub 20220428. doi: https://doi.org/10.3389/fnhum.2022.876935. PubMed PMID: 35572003; PubMed Central PMCID: PMCPMC9096085.

50. Rêgo ML, Cabral DA, Costa EC, Fontes EB. Physical exercise for individuals with hypertension: it is time to emphasize its benefits on the brain and cognition. Clinical Medicine Insights: Cardiology. 2019;13:1179546819839411. doi: https://doi.org/10.1177/1179546819839411.

Supporting information

S1 Table. Exercise intervention.

PLOS ONE

PONE-D-23-28550

Revision and Rebuttal #2

Short-term combined exercise training improves cognitive function in post-menopausal women

Dani Rahmat Ramadhana[1], Rizki Prayuda Putra[1], Michelle Abigail Sibarani[1], Sulistiawati[3], Dewi Ratna Sari[4], Purwo Sri Rejeki [1,2,5], Lilik Herawati [1,2,5], Raden Argarini[1,2,5*]

[1] Master Degree on Sport, Exercise, and Health Sciences, Faculty of Medicine, Airlangga University, Surabaya, Indonesia
[2] Undergraduate Medical Programme, Faculty of Medicine, Airlangga University, Surabaya, Indonesia
[3] Department of Public Health, Faculty of Medicine, Airlangga University, Surabaya, Indonesia
[4] Department of Anatomy, Histology and Pharmacology, Faculty of Medicine, Airlangga University, Surabaya, Indonesia
[5] Department of Medical Physiology and Biochemistry, Faculty of Medicine, Airlangga University, Surabaya, Indonesia

***Corresponding Author:**

Email: raden-a@fk.unair.ac.id (RA)

<table>
<tr><th colspan="2">Editor</th></tr>
<tr><td>Editor's comments:</td><td>Response</td></tr>
<tr><td>Thank you for submitting your manuscript to PLOS ONE. After careful consideration, we feel that it has merit but does not fully meet PLOS ONE’s publication criteria as it currently stands. Therefore, we invite you to submit a revised version of the manuscript that addresses the points raised during the review process.

Please submit your revised manuscript by May 20 2024 11:59 PM. If you will need more time than this to complete your revisions, please reply to this message or contact the journal office at plosone@plos.org. When you're ready to submit your revision, log on to https://www.editorialmanager.com/pone/ and select the 'Submissions Needing Revision' folder to locate your manuscript file.

Please include the following items when submitting your revised manuscript:
• A rebuttal letter that responds to each point raised by the academic editor and reviewer(s). You should upload this letter as a separate file labeled 'Response to Reviewers'.
• A marked-up copy of your manuscript that highlights changes made to the original version. You should upload this as a separate file labeled 'Revised Manuscript with Track Changes'.
• An unmarked version of your revised paper without tracked changes. You should upload this as a separate file labeled 'Manuscript'.

If you would like to make changes to your financial disclosure, please include your updated statement in your cover letter. Guidelines for resubmitting your figure files are available below the reviewer comments at the end of this letter.

If applicable, we recommend that you deposit your laboratory protocols in protocols.io to enhance the reproducibility of your results. Protocols.io assigns your protocol its own identifier (DOI) so that it can be cited independently in the future. For instructions see: https://journals.plos.org/plosone/s/submission-guidelines#loc-laboratory-protocols.

Additionally, PLOS ONE offers an option for publishing peer-reviewed Lab Protocol articles, which describe protocols hosted on protocols.io. Read more information on sharing protocols at https://plos.org/protocols?utm_medium=editorial-</td><td>Thank you for the opportunity to revise our manuscript. We have made changes accordingly.

We have submitted the main documents, including a rebuttal letter, track changes, and a clean version of the manuscript, which are required for the revision.

Thank you for your suggestion. However, the protocol has been registered on a clinical trial website. Therefore, we think that other protocols will not be necessary.</td></tr>
</table>

email&utm_source=authorletters&utm_campaign=protocols.<br><br>We look forward to receiving your revised manuscript.	
Journal Requirements:<br><br>Please review your reference list to ensure that it is complete and correct. If you have cited papers that have been retracted, please include the rationale for doing so in the manuscript text, or remove these references and replace them with relevant current references. Any changes to the reference list should be mentioned in the rebuttal letter that accompanies your revised manuscript. If you need to cite a retracted article, indicate the article's retracted status in the References list and also include a citation and full reference for the retraction notice.	We have thoroughly reviewed our references on http://retractiondatabase.org and found no retracted papers among our cited references.
**Additional Editor Comments:**<br><br>Thank you for submitting your valuable work.<br><br>Along with the remaining and important concerns to be addressed, please consider adapting a few parts:	
**Methods**	
1. It would be beneficial for the study to include a detailed explanation of how test scoring was conducted. Specifically, clarifying whether errors were calculated and describing the methods used to measure and analyse the outcomes would enhance understanding and replicability;	We have included a more comprehensive explanation of the test scoring.<br><br>See Pg. 10 Ln. 199
2. The adaptation of the Stroop test for Bahasa use is an interesting aspect of this study. However, lacks details on the standardisation procedures and the validation process of the adapted version. Providing this information is crucial for ensuring the test's reliability and validity, thereby strengthening the study's findings;<br><br>3. While it's commendable that intraclass correlation coefficients for reaction time and total error were included, the absence of information on inter-rater reliability for scoring the Stroop test is noted. Inter-rater reliability is important for confirming that scoring consistency is maintained across different evaluators, and its inclusion would solidify confidence in the scoring methodology;	Thank you for your suggestion. Unfortunately, the previous researcher did not report the inter-rater reliability value for this test.
4. The choice to utilise LMMs for assessing outcome variables is not explicitly justified. Elaborating on the reasons for choosing this modeling approach over others, such as ANOVA or regression models, would provide insight into its advantages for your analysis;	We have added the rationale for using linear mixed models in our analysis (Page 12, paragraph 1). Together with our previous statement (page 12, paragraph 1), we believe that the rationale for choosing

	this particular statistical analysis is clear. [This mixed modelling can deal efficiently with missing data, unbalanced time points [33] and robust with the violations in distributional assumptions [34]].
5. The ms briefly mentions employing a bootstrap procedure but didn't explain the specifics of its implementation or its influence on the reliability of SE and p-values. A more detailed description would aid in evaluating the statistical inference's robustness, enhancing the credibility of the findings;	We used a bootstrap confidence interval with 10,000 iterations to resample the mean difference. This resampling distribution of the difference in means approaches a normal distribution.<br><br>See Pg. 12 Ln. 243
6. Providing comprehensive details on scoring methodologies, standardisation, and validation processes will not only clarify the study's approach but also enable replication and verification by other researchers;	We have included a more comprehensive explanation of the test scoring.<br><br>See Pg. 10 Ln. 199
7. The study commendably reports high adherence to the exercise program, yet the lack of objective measures to monitor physical activity levels in the control group may undermine the validity of the intervention-control comparisons. Incorporating objective activity tracking, such as wearable devices, could strengthen the study's findings by ensuring accurate activity monitoring across all participants;	We acknowledge that our study did not include objective measurements to monitor daily physical activities in the control and exercise groups, such as using activity-tracking tools. Instead, an initial consent letter was used to dissuade participants from engaging in additional physical activities. Moreover, encouragement was provided through text messages to each participant in both groups to help them maintain their daily physical activities.<br><br>See Pg. 24 Ln. 132
8. While the focus on postmenopausal women provides valuable insights, the specific tailoring of the intervention to this group limits the broader applicability of the findings. Elaborate on how other could explore similar interventions in diverse demographic groups to enhance generalisation;	We have elaborated a statement to enhance generalization in the older population.<br><br>See Pg. 25 Ln. 461
9. The study's methodology could be enhanced by more thoroughly accounting for potential confounding factors such as education, socioeconomic status, and health conditions. Adjusting for these variables in the analysis would provide a clearer understanding of the intervention's effect;	We have reanalyzed the outcomes by adjusting for additional potential confounding factors, such as education and levels of anxiety, depression, and stress (measured by DASS-21 scores), which could impact the risk of cognitive decline. (See the table and figures). Other potential confounding factors, such as income, were not considered as we did not collect the data. Furthermore, all participants were unemployed and did not have any personal income.<br>See Pg. 24 Ln. 443

10. While the study reports on effect sizes, expanding the discussion to include the clinical relevance of these effects would offer deeper insights into their practical implications for cognitive function in postmenopausal women;	We have expanded the clinical relevance of these effects in the discussion section.<br><br>See Pg. 21 Ln. 370
11. While acknowledging the study's non-randomised design is important, a deeper exploration of other potential confounders, including baseline activity levels and medication use, would strengthen the study's conclusions by clarifying influences on the observed outcomes;	We have incorporated the impact of potential confounders on the study outcomes in the limitations section.<br><br>See Pg. 24 Ln. 443

<table>
<tr><th colspan="2">All Reviewer</th></tr>
<tr><td colspan="2">Thank you for reviewing our manuscript. Your comments were all accepted and changes have therefore been made to the sections you indicated. Thanks for helping us improve our submission to PLOS ONE.</td></tr>
<tr><td>Reviewer’s comments:</td><td>Response</td></tr>
<tr><td>1- Line 219: Indicate the statistical testing method which achieve 80% power. For example statistical testing methods are t-tests or chi-square tests, etc.
2- Indicate the underlying covariance structure used in the linear mixed model and the criteria for selecting it.
3- Indicate the number of iterations used in the bootstrap procedure.</td><td>Thank you for your positive and helpful responses.
1. We have included the statistical testing methods that we utilized in the sample calculation. (See page 11, line 222)
2. We have incorporated the underlying covariance structure (refer to page 12, line 232).
3. The number of iterations has been added on page 12, line 143.</td></tr>
<tr><td>Thank you! After reading the whole article, the author has answered all my questions and answered them well! There are a few questions

Point 1: “Fig 1. Diagram of participants flow (modified for non-randomized study)”and“Fig 1. Effects of exercise intervention on reaction time of the Stroop test performance in 270 postmenopausal women.” ! All are marked as Figure 1, please modify in order.</td><td>Thank you for your comment. We have made changes to the number of figures.</td></tr>
</table>
