# Supplementary material for: Short-term multicomponent exercise training improves executive function in postmenopausal women
Source: PLoS One. 2024 Aug 14;19(8):e0307812. doi: 10.1371/journal.pone.0307812 (PMC11324113; doi:10.1371/journal.pone.0307812)
Supplement: Attachment — Submitted filename: Response to Reviewers.docx [file pone.0307812.s005.docx]

**PLOS ONE**

**PONE-D-23-28550**

**Revision and Rebuttal**

**Short-term combined exercise training improves cognitive function in post-menopausal women**

**Dani Rahmat Ramadhana^1^, Rizki Prayuda Putra^1^, Michelle Abigail Sibarani^1^, Sulistiawati^3^, Dewi Ratna Sari^4^, Purwo Sri Rejeki ^1,2,5^, Lilik Herawati ^1,2,5^, Raden Argarini^1,2,5*^**

^1^ Master Degree on Sport, Exercise, and Health Sciences, Faculty of Medicine, Airlangga University, Surabaya, Indonesia

^2^ Undergraduate Medical Programme, Faculty of Medicine, Airlangga University, Surabaya, Indonesia

^3^ Department of Public Health, Faculty of Medicine, Airlangga University, Surabaya, Indonesia

^4^ Department of Anatomy, Histology and Pharmacology, Faculty of Medicine, Airlangga University, Surabaya, Indonesia

^5^ Department of Medical Physiology and Biochemistry, Faculty of Medicine, Airlangga University, Surabaya, Indonesia

***Corresponding Author:**

Email: [raden-a@fk.unair.ac.id](mailto:raden-a@fk.unair.ac.id) (RA)

| **Editor** | |
| --- | --- |
| Editor's comments: | Response |
| Thank you for submitting your manuscript to PLOS ONE. After careful consideration, we feel that it has merit but does not fully meet PLOS ONE’s publication criteria as it currently stands. Therefore, we invite you to submit a revised version of the manuscript that addresses the points raised during the review process.  Please submit your revised manuscript by Feb 09 2024 11:59PM. If you will need more time than this to complete your revisions, please reply to this message or contact the journal office at [plosone@plos.org](mailto:plosone@plos.org). When you're ready to submit your revision, log on to <https://www.editorialmanager.com/pone/> and select the 'Submissions Needing Revision' folder to locate your manuscript file.  Please include the following items when submitting your revised manuscript:   - A rebuttal letter that responds to each point raised by the academic editor and reviewer(s). You should upload this letter as a separate file labeled 'Response to Reviewers'. - A marked-up copy of your manuscript that highlights changes made to the original version. You should upload this as a separate file labeled 'Revised Manuscript with Track Changes'. - An unmarked version of your revised paper without tracked changes. You should upload this as a separate file labeled 'Manuscript'.   If you would like to make changes to your financial disclosure, please include your updated statement in your cover letter. Guidelines for resubmitting your figure files are available below the reviewer comments at the end of this letter.  Journal Requirements:  When submitting your revision, we need you to address these additional requirements.  1. Please ensure that your manuscript meets PLOS ONE's style requirements, including those for file naming. The PLOS ONE style templates can be found at <https://journals.plos.org/plosone/s/file?id=wjVg/PLOSOne_formatting_sample_main_body.pdf> and  <https://journals.plos.org/plosone/s/file?id=ba62/PLOSOne_formatting_sample_title_authors_affiliations.pdf> | Thank you for the opportunity to revise our manuscript. We have made changes accordingly.  We have submitted the main documents, including a rebuttal letter, track changes, and a clean version of the manuscript, along with supplementary materials such as the protocol and trial registration, which are required for the revision.  We have also made revisions to the financial disclosure as detailed below  This research is funded by the Directorate General of Higher Education, Research, and Technology - Ministry of Education, Culture, Research, and Technology based on Decree Number 0536/E5/PG.02.00/2023 and contract agreement Number 114/E5/PG.02.00.PL/2023; 1187/UN3.LPPM/PT.01.03/2023 |
| 2. The in-house editorial staff feels that your study meets the World Health Organization definition of a clinical trial because it is a prospective study in which participants were assigned to an exercise intervention to investigate the effects on cognitive function. Please change your manuscript’s article type to ‘Clinical Trial’ when you resubmit your manuscript. | We have changed the article type from “original research” to “clinical trial” |
| In addition, please provide the following important pieces of information/documentation.  1) Please note that you must upload a completed CONSORT flowchart as figure 1 of your manuscript and the CONSORT checklist as a supporting information file. Blank copies of these documents and information regarding CONSORT can be found via the following link: <http://www.consort-statement.org/>. If your clinical trial uses a non-randomized design, you may wish to submit a TREND checklist (<http://www.cdc.gov/trendstatement>), in place of the CONSORT checklist; a flowchart is still required.” | Thank you for your suggestion.   1) We have added a modified CONSORT flowchart for non-randomized trials as Figure 1. Additionally, a TREND checklist has been submitted as non-review material. |
| 2) Please upload a copy of your trial study protocol as a supporting information file. By the study protocol, we mean the complete and detailed plan for the conduct and analysis of the trial that the ethics committee approved before the trial began. Please send this in the original language. If this is in a language other than English, please also provide a translation. Please detail any deviations from this study protocol in the Methods section of your manuscript. Your study protocol will be made available to the editors and reviewers, and will be published as supporting information with your manuscript if accepted for publication. (If you do not agree to this, we will not be able to publish your manuscript). If you have formally published a study protocol for your trial in a journal then you should cite this in your manuscript, but you still need to send us the original document. | 2) We have uploaded a copy of our study protocol and an English translation of the protocol. |
| 3) PLOS ONE requires that all clinical trials are registered in an appropriate registry (the WHO list of approved registries is at <http://www.who.int/ictrp/network/primary/en/index.html> and more information on trial registration is at <http://www.icmje.org/about-icmje/faqs/clinical-trials-registration/>). Please state the name of the registry and the registration number (e.g. ISRCTN or ClinicalTrials.gov) in the submission data and on the title page of your manuscript.  If you have not yet registered your trial in an appropriate registry, we now require you to do so and will need confirmation of the trial registry number. Please include in the Methods section of your paper your reasons for not registering this study before enrolment of participants started. Please confirm that all related trials are registered by stating: “The authors confirm that all ongoing and related trials for this drug/intervention are registered”. Please see <http://journals.plos.org/plosone/s/submission-guidelines#loc-clinical-trials> for our policies on clinical trials. | 3) We have registered the trial. In ISRCTN with reference number ISRCTN13086152. The details of the registry can be found at the following link: <https://www.isrctn.com/ISRCTN13086152>   We have also included statements in the methods section explaining the reason why this study was registered prospectively.  See Pg. 6 Ln. 124 |
| 3. We note that the grant information you provided in the ‘Funding Information’ and ‘Financial Disclosure’ sections do not match.  When you resubmit, please ensure that you provide the correct grant numbers for the awards you received for your study in the ‘Funding Information’ section. | Thank you for your suggestion. We have made changes to the grant information.  This research is funded by the Directorate General of Higher Education, Research, and Technology - Ministry of Education, Culture, Research, and Technology based on Decree Number 0536/E5/PG.02.00/2023 and contract agreement number 114/E5/PG.02.00.PL/2023; 1187/UN3.LPPM/PT.01.03/2023 |
| Additional Editor Comments:  Thank you for submitting your valuable work.  The reviews, which are insightful and interesting, pointed to some unexplained aspects. The authors will notice the reviewers found merits in your study, but also raised important concerns. Nevertheless, authors also will notice that this may sound lengthy, but the main intention is to work more closely with the suggestions (while also ensuring the high standard of scientific communication).  I was reluctant to provide inputs in this round, but upon re-reading the comments, I think the authors address them in due time. | |
| **TITLE**  The Title would benefit from increased specificity. For instance, specifying the cognitive functions and types of exercises would enhance clarity. As I understand that this can be the authors' perspective to make it more 'punchy', I don't think there's this need to change a lot.  Consider:  a. the Title effectively convey the study's main idea and objectives? and  b. From a broader audience perspective outside the field, is there any options to improve understanding? | We have revised the title and changed the cognitive function to executive function as the main outcome of our study. |
| **ABSTRACT** | |
| The Abstract needs improvement by providing exercise descriptions, addressing strengths and limitations, and refining sentences for readability. | We have added detailed exercise descriptions and refined the sentences. |
| Consider placing mean age after "50-80 years" and use an abbreviation other than the one provided for exercise to avoid confusion; | We have replaced "50-80 years" with the mean age |
| Ensure decimal consistency | We used 2 decimal points for the overall results and 3 decimal points for p value. |
| For the aged groups, maybe the MoCA, instead of the MMSE, would be a good option. As this might cause misinterpretation by researchers and readers outside the field, consider tempering language re. "global function." | Thank you for recommending the use of MoCA instead of MMSE for the cognitive test. We understand that MoCA is superior in terms of sensitivity and specificity for detecting mild cognitive impairment. However, the MMSE is the most widely used cognitive measurement tool, and we are more familiar with this tool instead of the MoCA. One study on the aging population in our country, Indonesia, revealed a strong correlation between MoCA and MMSE scores (https://www.ncbi.nlm.nih.gov/pmc/articles/PMC5771294/). Furthermore, the MMSE is brief and therefore more convenient to use in our study. We will consider using MoCA in our future study. |
| Avoid capitalising "pre" and "post" and refine terms for clarity; | We have replaced and refined the words as per your suggestion. |
| Emphasise effect sizes over p-values | We have specified the effect size. |
| **INTRODUCTION** | |
| 3. Provide additional context on how menopause influences cognitive function. The text lacks background and rationale, crucial for understanding authors' exercise choices and their neural links; | Thank you for your suggestion. We have added a paragraph to explain how menopause influences cognitive function.  See Pg. 3, ln. 54 |
| **METHODS** | |
| 4. Please provide more details on the sample, especially demographics. Provide a detailed overview of eligibility, recruitment, and workflow; | We have provided the details of participant flow in the CONSORT diagram (see Figure 1, Pg. 8 Ln. 160). |
| 5. Enhance transparency by detailing data recording and adherence to best practices making data available; | We have added the details about adherence in the result section (see Pg. 11, ln. 244) |
| 6. Consider including more details on tests, such as reliability coefficients, Cronbach's alpha. Please consider the use of illustration to explain the procedures for the tests. Also present the extracted variables; | Thank you for your suggestion. We have added a reliability coefficient for the Stroop test (Pg. 10, ln. 188) and MMSE (Pg.11, ln. 211) |
| 7. Please refine the stats section with information on effect sizes, confidence intervals, and rationale for tests used for analyses. Why use a risky post-hoc? Any corrections to? Consider that your model can be inflated and your analyses would be better interpreted if you could simplify the models;  8. I'd highly suggest checking again your parameters, skewness, kurtosis and the chosen tests. Seems like the model without considering interaction within- is worrying. I genuinely think that LMM is more appropriate. Also, please consider that PRISM not always provides the reliable details - you can consider other handy software that are quick and won't take that much time, like Jamovi or JAPS; | Thank you for your suggestion. We have reanalyzed the statistical data using a linear mixed model in JAMOVI. The data is presented in Table 2, Fig. 2, and Fig. 3. |
| 9. Clearly explain how missing data were handled if observed; | We used linear mixed model analysis, which can efficiently handle missing data without the need for imputation. Moreover, there was no missing data in this study.  See Pg. 12, ln. 228 |
| 10. Please, if possible, utilise dispersion data in graphs, configuring options for boxes and scatter, for example; | Thank you for your suggestion. We have utilized dispersion data for the graph.  (Fig 2-4) |
| 11. Consider that Discussion needs a little bit working, tightening up to your findings and what previous studies observed; | Thank you for your suggestion, we have made changes to discussion part accordingly. |
| 12. I think the authors could consider to refine Discussion presenting the strengths and limitations; | Thank you for your suggestion. We have added a separate paragraph presenting the strengths and limitations of our study.  Pg 23 ln 395 |
| 13. Carefully check the refs. list for adherence to Journal's standards; | We have revised our references. |
| Overall, the study is, indeed, interesting and conducted in the proper way, but the lack of specifications, details and the stats presentation are the main hindrances of this version. Apart from it, theoretical contributions could be given for readers and researchers, so they can understand the generalisation and the relevance of your study - even if they are outside the field.  Hope the authors find all (or most) of the suggestions useful and hope this helps somehow.  Remember to adhere to the standards of the journal and make, if possible, your data available.  The authors are highly encouraged to enlist a native English speaker to make some edits.  Wishing you success with the study. If you find yourself approaching the due date, feel free to request a bit more time.  Please do not be concerned about time constraints; simply demonstrate the same level of dedication you exhibited during the study. | Thank you for your positive comments. We have proofread this manuscript and made revisions according to your suggestions. |

| **Reviewer #1** | |
| --- | --- |
| *Thank you for reviewing our manuscript. Your comments were all accepted and changes have therefore been made to the sections you indicated. Thanks for helping us improve our submission to PLOS ONE.* | |
| Reviewer’s comments: | Response |
| In this research authors investigated the effects of a multi-component exercise program on cognitive function of post-menopausal women. The study deals with an interesting topic. However it has some important limitations: very short training program; lack of randomization; lack of a proper control group | Thank you for your positive and helpful responses.  The type of exercise was chosen based on previous studies that suggest that adding at least one type of exercise to aerobic exercise or multicomponent exercise for older individuals is more beneficial for improving cognitive function. Regarding the short intervention, we have added some sentences to justify our chosen timing.  We realize that our study has some limitations; however, I believe that our findings may be valuable contributions to the scientific community. |
| **Introduction:** |  |
| Authors should specify which is the first study endpoint (according to what is stated in the statistical paragraph it should be changes (post versus pre exercise) in Stroop test. | Thank you for your suggestion. We have made some changes based on the main outcome of this study. |
| **Methodology** | |
| The choice of a short exercise training program in the present study (only two weeks) need to be justified and supported with previous researches. In my opinion some important aspects of the training program (such as intensity of different exercises) are missing. Authors should provide these details in the method section  It is not clear to me why the authors gave up on randomization. | Thank you for your suggestion. We developed a 2-week multicomponent exercise program to be more feasible in the community setting. We included some references to support our exercise intervention. (Pg. 7 ln. 152).  The population characteristics included in this study are older women. In our country, most residents are unable to travel far from their housing complexes. Therefore, we abandoned randomization and instead selected participants who lived near the community hall, recruiting them through the Family Welfare Empowerment Program.  Moreover, the participation rate in physical exercise and research is low in our country. Adherence to the exercise protocol is crucial for achieving the desired effects on cognitive function, and emphasis should be placed on the structure of the short program. Therefore, we have developed a protocol to reduce the burden on both participants and researchers. As shown in our results, we can maintain a high adherence rate to the exercise program. |
| **Discussion** |  |
| In my opinion this paragraph is more like a second introduction. Therefore it should be re-organized. Results obtained in his research should be compared with previous similar researches and differences should be underlined. | Thank you for your suggestions. We have made changes to the discussion accordingly. |

| **Reviewer #2** | |  |
| --- | --- | --- |
| Reviewer and Comment | Response | |
| *Thank you for reviewing our manuscript. Your comments were all accepted and changes have therefore been made to the sections you indicated. Thanks for helping us improve our submission to PLOS ONE.* | |  |
| The authors have clearly stated that the purpose of the study was to evaluate investigate the effects of short-term combined exercise training programs on cognitive function in post- menopausal women. The paper is well-written, easy to follow and adds merit to the vital role of exercise training in the cognitive function. Given this approach, this work can enhance future attempts in similar research area. However, I have highlighted a few suggestions and concerns in my specific comments section (below) that need to be addressed before considering whether this work should be published or not. In summary, this is an innovative and work that might be considered for publication after revising the initial manuscript as needed. | Thank you for your positive and helpful responses |  |
| **ABSTRACT** - Replace mean values ± SD with Δ% and effect sizes per outcome measure in results. | We have reanalyzed the data using linear mixed models and included the mean difference and effect sizes. | |
| **INTRODUCTION**  - Add a sentence about the beneficial role of combined training in health among adults according to the latest guidelines by the World Health Organization (1). - Add a statement about the popularity of both aerobic and resistance training in the health and fitness industry worldwide according to the latest report published by the American College of Sports Medicine (2).  Suggested References: 1. <https://pubmed.ncbi.nlm.nih.gov/33239350/> 2. <https://journals.lww.com/acsm-healthfitness/pages/articleviewer.aspx?year=2022&issue=01000&article=00007&type=Fulltext> | Thank you for your suggestion; we have added sentences regarding  - The beneficial role of combined training in adult health  - The popularity of both aerobic and resistance training in the health and fitness industry. (Pg. 5 ln. 103) |  |
| **Discussion:** |  | |
| 1. Discuss the results observed for combined training with respect to various populations (3) as a critical piece of overall health improvements. Suggested reference: 3. <https://pubmed.ncbi.nlm.nih.gov/35477256/> | Thank you for your suggestion. We have incorporated the benefits of combined exercise training into health-related outcomes. (Pg 22, ln 380) |  |
| 2. Strengths and limitations should be presented in a separate paragraph at the end of the discussion section. A heading is also needed. | We have added a separate paragraph to present the strength and limitation of our study. (Pg 23 ln. 395) |  |
| **CONCLUSIONS** | |  |
| In conclusions, you should underline the main findings and suggest future research attempts in this area while highlighting potential practical implications. This paragraph needs a significant improvement. | Thank you for your suggestion, we have made changes in discussion according to your suggestion | |

| **Reviewer 3** | |
| --- | --- |
| Reviewer and Comment | Response |
| *Thank you for reviewing our manuscript. Your comments were all accepted and changes have therefore been made to the sections you indicated. Thanks for helping us improve our submission to PLOS ONE.* | |
| The research design of this study is relatively reasonable, the experimental control is relatively appropriate, and the data statistics are relatively rigorous. The result data can support the conclusion. The logical thinking and language expression of the article are relatively clear. However, the author did not seem to indicate whether the experiment concealed the experimental purpose from the participants (blind design). Will this cause some bias in the research results? In addition, the author does not seem to clearly indicate why "Stroop Test" and "Mini Mental State Examination" were chosen to evaluate the cognitive function of the participants. If a more detailed explanation of these two assessment methods (the use of previous scholars) is added in the introduction part, will it be better? | Thank you for your positive and helpful responses.  The type of intervention in this study may not possible to conceal from participants. Only the data analyst who was blinded to the participant allocation (Page 8, ln. 160)  We have added a brief explanation regarding the cognitive task chosen for this study. (Page 5, Ln.104) |

| **Reviewer 4** | |
| --- | --- |
| Reviewer and Comment | Response |
| *Thank you for reviewing our manuscript. Your comments were all accepted and changes have therefore been made to the sections you indicated. Thanks for helping us improve our submission to PLOS ONE.* | |
| It is an interesting topic, Let me understand the effect of short-term comprehensive training on the cognitive function of postmenopausal women. But I think some parts of the article need further revision to ensure the quality of the article. | Thank you for your positive and helpful responses |
| *Summary* The overall structure of the article is still a little confused, should focus on modification.  Point 1: The manuscript should be checked by a native speaker before publication. | Point 1: Thank you for your suggestion. We have proofread this article. |
| Point 2: There are too many old references that are more than 10 years old; please replace all possible references, with articles within the last 5 years. | Point 2: Thank you for your suggestion; we have made changes to the references where possible. |
| **INTRODUCTION**  Point 3:Page1 Line54-56. “Furthermore,.....sensitive”.This paragraph says that women are more likely to get Alzheimer's disease, but how is it directly relevant to this article, or to clarify its relationship to cognitive function in postmenopausal women.  Point 4:The second paragraph focuses on the fact that exercise can improve cognitive function in the elderly. But there was no mention of whether exercise improved cognitive function in postmenopausal women. It is because there is no similar literature research, or not written in the article, I suggest adding.  Point 5:Page4 Line75-83. This paragraph again talks about the relationship between motor and cognitive function, similar to the content of the second paragraph, it is recommended to merge.  Point 6: The research background is not very detailed, and it is not clear why such research is done, because of the lack of previous studies, or what other reasons. | Point 3: We have rephrased the sentence. This statement exemplifies that postmenopausal women are more susceptible to cognitive decline-related diseases than men. (Pg.3 ln. 52)  Point 4: Thank you for your suggestion. We have included the pathophysiology of cognitive decline in postmenopausal women in the introduction (paragraph 2) and discussed possible mechanisms through which exercise can counteract this issue (paragraph 3).  Point 5: Thank you for your suggestion. This paragraph has been merged with paragraph 2 (now paragraph 3).  Point 6: We have added the reasons in the background of the manuscript. |
| **Materials and methods**  Point 7: Page4 Line23-24. “ They were also screened to ensure that they are not having specific diseases as mentioned earlier in the inclusion and exclusion criteria”. This sentence is repeated with the inclusion criteria of Participants characteristic, so it is recommended to delete it  Point 8: Page4 Line28. “The psychological stress was also assessed using the Depression Anxiety Stress Scale-21 (DASS-21) to ensure that they were not experiencing moderate-severe stress”.It is suggested to put this sentence in the previous paragraph.  Point 9: Page6 Line118-120.Whether the experimental group and the control group were assigned randomly, and by what specific way.If it is not a randomized controlled trial, please indicate what kind of study it is. | Thank you for your suggestion:  Point 7: We have deleted this statement  Point 8. We have rearranged this sentence and placed it in the previous paragraph. (Pg.7 Ln. 141).  Point 9. On page 6, lines 118-120, we did not mention the random assignment. We have added the consort flow diagram to prevent confusion regarding participant allocation into the experimental and control groups. |
| Point 10: Page6 Line124-125. “All the measurements were performed in a controlled-temperature room (25℃) at the same time (06:00 – 08:00 AM)”.It is suggested to put this sentence in the previous paragraph | Point 10: We have rearranged the sentences in the previous paragraph. |
| Point 11: It is recommended to include a picture to show how the subjects were assigned, whether there were drop-outs, and the number of people analyzed at the end. | Point 11. As mentioned in point 9, Figure 1 has been included to provide a detailed explanation of the recruitment process up to the data analysis stage. |
| Point 12: Page7 Line150.  Change the Cognitive test to Outcome measurements. | Point 12. We have change the cognitive test to outcome measurement |
| **Results** Point13:“P＜0.05”should be write as“p＜0.05”. | We have changed the way we write the p-value. |
| **Discussion** The discussion section is well written | Thank you for your positive comment. |
| Point 14: In the discussion section, some specific strategies for exercise intervention in cognitive function of postmenopausal women should be added. | Thank you for your suggestion.  We have included strategies for exercise intervention to improve cognitive function in older women. However, due to the limited evidence from studies with only female participants, we extrapolated this recommendation from a study that included mostly female participants. To the best of our knowledge, no meta-analysis has been conducted on exercise interventions specific to older women regarding the recommended type and dose of exercise. |

| **Reviewer 5** | |
| --- | --- |
| Reviewer and Comment | Response |
| *Thank you for reviewing our manuscript. Your comments were all accepted and changes have therefore been made to the sections you indicated. Thanks for helping us improve our submission to PLOS ONE.* | |
| A two-arm controlled study was conducted which aimed to investigate the effects of short-term combined exercise training programs on cognitive function in post-menopausal women. After a two-week exercise program, the study showed an improvement in global cognitive function of the exercise group. | Thank you for your positive and helpful responses |
| Minor revisions: 1. Line 183: Indicate the statistical testing method which achieves 80% power. 2. Indicate if the distribution of the data was checked for normality prior to applying t-tests or ANOVA. 3. Perform tests of interactions prior to testing main effects. If the interaction effect is significant, provide an interpretation of the results, but do not test main effects because the tests for main effects are uninteresting in light of significant interactions. If interaction effects are non-significant, drop the interaction effects from the model and test the main effects. Determining which results to present when testing interactions is often a multi-step process. 4. Indicate the date range subjects participated in the study. | Thank you for your suggestion; we have made changes accordingly.  1. Power (80%) has been incorporated into the statistical method (Page 11, line 219)  2. The distribution of the data has been checked. In terms of baseline characteristics, all variables are normally distributed. However, some of the outcome variables are not normally distributed for the residuals. We provided a detailed explanation of this in the statistical analysis section.  3. We have reanalyzed our results using a linear mixed model as suggested by the editor. We presented the interaction effect to interpret our results.  4. The date range has been added to the methods of this study (Pg. 6 Ln. 122). |
