# Supplementary material for: Short-term multicomponent exercise training improves executive function in postmenopausal women
Source: PLoS One. 2024 Aug 14;19(8):e0307812. doi: 10.1371/journal.pone.0307812 (PMC11324113; doi:10.1371/journal.pone.0307812)
Supplement: Attachment — Submitted filename: peer review.doc [file pone.0307812.s006.doc]

Thank you! After reading the whole article, the author has answered all my questions and answered them well! There are a few questions

**Point 1: “Fig 1. Diagram of participants flow (modified for non-randomized study)”and“Fig 1. Effects of exercise intervention on reaction time of the Stroop test performance in 270**

**postmenopausal women.”！All are marked as Figure 1, please modify in order.**
