# Supplementary material for: Short-term multicomponent exercise training improves executive function in postmenopausal women
Source: PLoS One. 2024 Aug 14;19(8):e0307812. doi: 10.1371/journal.pone.0307812 (PMC11324113; doi:10.1371/journal.pone.0307812)
Supplement: Attachment — Submitted filename: Response to Reviewers_2.docx [file pone.0307812.s007.docx]

**PLOS ONE**

**PONE-D-23-28550**

**Revision and Rebuttal #2**

**Short-term combined exercise training improves cognitive function in post-menopausal women**

**Dani Rahmat Ramadhana^1^, Rizki Prayuda Putra^1^, Michelle Abigail Sibarani^1^, Sulistiawati^3^, Dewi Ratna Sari^4^, Purwo Sri Rejeki ^1,2,5^, Lilik Herawati ^1,2,5^, Raden Argarini^1,2,5*^**

^1^ Master Degree on Sport, Exercise, and Health Sciences, Faculty of Medicine, Airlangga University, Surabaya, Indonesia

^2^ Undergraduate Medical Programme, Faculty of Medicine, Airlangga University, Surabaya, Indonesia

^3^ Department of Public Health, Faculty of Medicine, Airlangga University, Surabaya, Indonesia

^4^ Department of Anatomy, Histology and Pharmacology, Faculty of Medicine, Airlangga University, Surabaya, Indonesia

^5^ Department of Medical Physiology and Biochemistry, Faculty of Medicine, Airlangga University, Surabaya, Indonesia

***Corresponding Author:**

Email: [raden-a@fk.unair.ac.id](mailto:raden-a@fk.unair.ac.id) (RA)

| **Editor** | |
| --- | --- |
| Editor's comments: | Response |
| Thank you for submitting your manuscript to PLOS ONE. After careful consideration, we feel that it has merit but does not fully meet PLOS ONE’s publication criteria as it currently stands. Therefore, we invite you to submit a revised version of the manuscript that addresses the points raised during the review process.  Please submit your revised manuscript by **May 20 2024 11:59 PM.** If you will need more time than this to complete your revisions, please reply to this message or contact the journal office at [plosone@plos.org](mailto:plosone@plos.org). When you're ready to submit your revision, log on to <https://www.editorialmanager.com/pone/> and select the 'Submissions Needing Revision' folder to locate your manuscript file.  Please include the following items when submitting your revised manuscript:   - A rebuttal letter that responds to each point raised by the academic editor and reviewer(s). You should upload this letter as a separate file labeled 'Response to Reviewers'. - A marked-up copy of your manuscript that highlights changes made to the original version. You should upload this as a separate file labeled 'Revised Manuscript with Track Changes'. - An unmarked version of your revised paper without tracked changes. You should upload this as a separate file labeled 'Manuscript'.   If you would like to make changes to your financial disclosure, please include your updated statement in your cover letter. Guidelines for resubmitting your figure files are available below the reviewer comments at the end of this letter.  If applicable, we recommend that you deposit your laboratory protocols in [protocols.io](http://protocols.io/) to enhance the reproducibility of your results. Protocols.io assigns your protocol its own identifier (DOI) so that it can be cited independently in the future. For instructions see: <https://journals.plos.org/plosone/s/submission-guidelines#loc-laboratory-protocols>.  Additionally, PLOS ONE offers an option for publishing peer-reviewed Lab Protocol articles, which describe protocols hosted on [protocols.io](http://protocols.io/). Read more information on sharing protocols at <https://plos.org/protocols?utm_medium=editorial-email&utm_source=authorletters&utm_campaign=protocols>.  We look forward to receiving your revised manuscript. | Thank you for the opportunity to revise our manuscript. We have made changes accordingly.  We have submitted the main documents, including a rebuttal letter, track changes, and a clean version of the manuscript, which are required for the revision.  Thank you for your suggestion. However, the protocol has been registered on a clinical trial website. Therefore, we think that other protocols will not be necessary. |
| Journal Requirements:  Please review your reference list to ensure that it is complete and correct. If you have cited papers that have been retracted, please include the rationale for doing so in the manuscript text, or remove these references and replace them with relevant current references. Any changes to the reference list should be mentioned in the rebuttal letter that accompanies your revised manuscript. If you need to cite a retracted article, indicate the article’s retracted status in the References list and also include a citation and full reference for the retraction notice. | We have thoroughly reviewed our references on http://retractiondatabase.org and found no retracted papers among our cited references. |
| **Additional Editor Comments:**  Thank you for submitting your valuable work.  Along with the remaining and important concerns to be addressed, please consider adapting a few parts: |  |
| **Methods** |  |
| 1. It would be beneficial for the study to include a detailed explanation of how test scoring was conducted. Specifically, clarifying whether errors were calculated and describing the methods used to measure and analyse the outcomes would enhance understanding and replicability; | We have included a more comprehensive explanation of the test scoring.  See Pg. 10 Ln. 199 |
| 2. The adaptation of the Stroop test for Bahasa use is an interesting aspect of this study. However, lacks details on the standardisation procedures and the validation process of the adapted version. Providing this information is crucial for ensuring the test's reliability and validity, thereby strengthening the study's findings;  3. While it's commendable that intraclass correlation coefficients for reaction time and total error were included, the absence of information on inter-rater reliability for scoring the Stroop test is noted. Inter-rater reliability is important for confirming that scoring consistency is maintained across different evaluators, and its inclusion would solidify confidence in the scoring methodology; | Thank you for your suggestion. Unfortunately, the previous researcher did not report the inter-rater reliability value for this test. |
| 4. The choice to utilise LMMs for assessing outcome variables is not explicitly justified. Elaborating on the reasons for choosing this modeling approach over others, such as ANOVA or regression models, would provide insight into its advantages for your analysis; | We have added the rationale for using linear mixed models in our analysis (Page 12, paragraph 1). Together with our previous statement (page 12, paragraph 1), we believe that the rationale for choosing this particular statistical analysis is clear. [This mixed modelling can deal efficiently with missing data, unbalanced time points [33] and robust with the violations in distributional assumptions [34]]. |
| 5. The ms briefly mentions employing a bootstrap procedure but didn't explain the specifics of its implementation or its influence on the reliability of SE and p-values. A more detailed description would aid in evaluating the statistical inference's robustness, enhancing the credibility of the findings; | We used a bootstrap confidence interval with 10,000 iterations to resample the mean difference. This resampling distribution of the difference in means approaches a normal distribution.  See Pg. 12 Ln. 243 |
| 6. Providing comprehensive details on scoring methodologies, standardisation, and validation processes will not only clarify the study's approach but also enable replication and verification by other researchers; | We have included a more comprehensive explanation of the test scoring.  See Pg. 10 Ln. 199 |
| 7. The study commendably reports high adherence to the exercise program, yet the lack of objective measures to monitor physical activity levels in the control group may undermine the validity of the intervention-control comparisons. Incorporating objective activity tracking, such as wearable devices, could strengthen the study's findings by ensuring accurate activity monitoring across all participants; | We acknowledge that our study did not include objective measurements to monitor daily physical activities in the control and exercise groups, such as using activity-tracking tools. Instead, an initial consent letter was used to dissuade participants from engaging in additional physical activities. Moreover, encouragement was provided through text messages to each participant in both groups to help them maintain their daily physical activities.  See Pg. 24 Ln. 132 |
| 8. While the focus on postmenopausal women provides valuable insights, the specific tailoring of the intervention to this group limits the broader applicability of the findings. Elaborate on how other could explore similar interventions in diverse demographic groups to enhance generalisation; | We have elaborated a statement to enhance generalization in the older population.  See Pg. 25 Ln. 461 |
| 9. The study's methodology could be enhanced by more thoroughly accounting for potential confounding factors such as education, socioeconomic status, and health conditions. Adjusting for these variables in the analysis would provide a clearer understanding of the intervention's effect; | We have reanalyzed the outcomes by adjusting for additional potential confounding factors, such as education and levels of anxiety, depression, and stress (measured by DASS-21 scores), which could impact the risk of cognitive decline. (See the table and figures). Other potential confounding factors, such as income, were not considered as we did not collect the data. Furthermore, all participants were unemployed and did not have any personal income.  See Pg. 24 Ln. 443 |
| 10. While the study reports on effect sizes, expanding the discussion to include the clinical relevance of these effects would offer deeper insights into their practical implications for cognitive function in postmenopausal women; | We have expanded the clinical relevance of these effects in the discussion section.  See Pg. 21 Ln. 370 |
| 11. While acknowledging the study's non-randomised design is important, a deeper exploration of other potential confounders, including baseline activity levels and medication use, would strengthen the study's conclusions by clarifying influences on the observed outcomes; | We have incorporated the impact of potential confounders on the study outcomes in the limitations section.  See Pg. 24 Ln. 443 |

| **All Reviewer** | |
| --- | --- |
| *Thank you for reviewing our manuscript. Your comments were all accepted and changes have therefore been made to the sections you indicated. Thanks for helping us improve our submission to PLOS ONE.* | |
| Reviewer’s comments: | Response |
| 1- Line 219: Indicate the statistical testing method which achieve 80% power. For example statistical testing methods are t-tests or chi-square tests, etc. 2- Indicate the underlying covariance structure used in the linear mixed model and the criteria for selecting it. 3- Indicate the number of iterations used in the bootstrap procedure. | Thank you for your positive and helpful responses.  1. We have included the statistical testing methods that we utilized in the sample calculation. (See page 11, line 222)  2. We have incorporated the underlying covariance structure (refer to page 12, line 232).  3. The number of iterations has been added on page 12, line 143. |
| Thank you! After reading the whole article, the author has answered all my questions and answered them well! There are a few questions    **Point 1: “Fig 1. Diagram of participants flow (modified for non-randomized study)”and“Fig 1. Effects of exercise intervention on reaction time of the Stroop test performance in 270**  **postmenopausal women.”！All are marked as Figure 1, please modify in order.** | Thank you for your comment. We have made changes to the number of figures. |
